# Ultrastrong underwater adhesion on diverse substrates using non-canonical phenolic groups

**DOI:** 10.1038/s41467-022-29427-w

**Published:** 2022-04-13

**Authors:** Bohan Cheng, Jinhong Yu, Toma Arisawa, Koki Hayashi, Joseph J. Richardson, Yasushi Shibuta, Hirotaka Ejima

**Affiliations:** Department of Materials Engineering, School of Engineering, The University of Tokyo, 7-3-1 Hongo, Bunkyo-ku, Tokyo, 113-8656 Japan

**Keywords:** Bioinspired materials, Polymer synthesis, Polymers

## Abstract

Robust underwater adhesion is challenging because a hydration layer impedes the interaction between substrates and adhesives. Phenolic adhesives inspired by marine creatures such as mussels were extensively studied, but these adhesives have not reached the adhesion strength and substrate diversity of Man-made dry adhesives. Here, we report a class of ultrastrong underwater adhesives with molecular phenolic designs extending beyond what nature has produced. These non-canonical phenolic polymers show versatile adhesion on various materials, with adhesion strengths exceeding 10 MPa on metal. Incorporating even just a small amount (<10%) of non-canonical phenolic groups into a polymer is sufficient for dramatically enhancing underwater adhesion, suggesting that this new class of phenolic materials will be incorporated into various industrial polymer systems in the future.

## Abstract (web summary)

Realizing robust underwater adhesion is challenging because a hydration layer impedes the interaction between substrates and adhesives. Here, the authors report a class of ultrastrong underwater adhesives with molecular non-canonical phenolic groups in a polymer to realize strong adhesion on various substrates.

## Introduction

Strong adhesion in wet environments is required in medical and marine industries^1^, however, a hydration layer often hinders interactions between adhesives and surfaces^2,3^. Conventional adhesives including cyanoacrylates, epoxy resins, and polyurethanes work well in dry environments but do not perform as well underwater^4,5^. Alternatively, bioinspired phenolic polymers are emerging as promising alternates^6,7^, as the Byssal threads of mussels can attach to wet surfaces using adhesive proteins capable of penetrating hydration layers on different substrates. These adhesive proteins contain amino acids with catechol groups, namely 3,4-dihydroxyphenylalanine (DOPA)^8,9^. Inspired by this, catechol-functionalized polymers have been extensively studied for underwater adhesion mainly with respect to backbone structure^10^, topology^4,11^, type of co-monomer^12^, molecular weight^12–15^, and coacervation^16^. Despite increasing the adhesion of synthetic polymers, the adhesion strengths of catechol polymers are still significantly weaker than dry adhesives.

The gallol group^17^, which occurs naturally in plants and has one more hydroxy group than the catechol group used by mussels, has recently received attention for designing underwater and medical adhesives^18,19^. For example, our previous reports^17,20^ demonstrated that gallol-based underwater adhesives showed stronger adhesion than catechol-based ones. As simply adding one more hydroxy group led to stronger adhesion, it is expected that non-canonical phenolic groups with 4 or 5 hydroxyl groups per aromatic ring would exhibit even stronger adhesion capability. However, the adhesive polymers containing these moieties do not occur naturally and no reports exist on their synthesis.

Here, we report the synthesis of non-canonical phenolic polymers with four and five hydroxyl groups on one styrene monomer unit that demonstrate ultrastrong underwater adhesion on various substrates. Due to the remarkable adhesion strength of these non-canonical phenolic groups, only a small amount (5–10% by molar ratio) needs to be incorporated into a polystyrene (PS) backbone to yield outstanding underwater adhesion on different substrates (up to >10 MPa). Through a systematic investigation of 13 phenolic group permutations, we demonstrate that not only the number but also the position of the hydroxyl groups determine the underwater adhesion strength. Due to the non*-*covalent nature of these non-canonical phenolic adhesives, they can readily be collected from substrates by rinsing or sonication in common organic solvents and recycled without significant performance deterioration. In addition, the homopolymers with 4 or 5 hydroxyl groups had good water solubility (70 mg/mL for homopolymer with 4 hydroxyl groups and 40 mg/mL for homopolymer with 5 hydroxyl groups), allowing them to potentially find use in biomedical applications.

## Results and discussion

### Synthesis and performance of non-canonical phenolic polymers

**Fig. 1:**
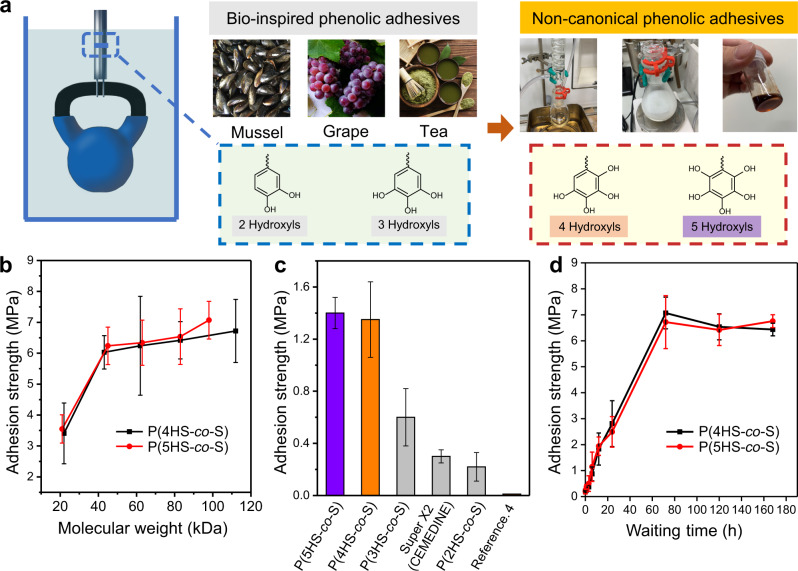
Synthesis and performance of phenolic underwater adhesives. **a** Comparison of bio-inspired (catechol and gallol) phenolic adhesives to non-canonical (4 and 5 hydroxyl residue) phenolic adhesives. **b** *M*_n_ dependence of adhesion for P(4HS*-co*-S) and P(5HS*-co*-S). The results represent the mean ± s.d. (*n* = 5). **c** Adhesion strengths of different adhesives with optimized *M*_n_ on steel after waiting for 10 s. The results represent the mean ± s.d. (*n* = 5). **d** Waiting time dependence of adhesion strengths of P(4HS*-co*-S) and P(5HS*-co*-S). The results represent the mean ± s.d. (*n* = 5).

Styrene-type monomers have simple chemical structures and thus are suitable models for comparing the influence of the number of phenolic groups on adhesion behavior. We synthesized non-canonical monomers, namely 2,3,4,5-tetramethoxystyrene (TMS) and 2,3,4,5,6-pentamethoxystyrene (PMS) (Supplementary Figs. 1–3) for subsequent polymerization. TMS was obtained by radical bromination of 2,3,4,5-tetramethoxytoluene and subsequently modified using a one-pot Wittig reaction^21^. This cost-effective synthesis could be completed in 16 h with a moderate yield. For PMS, additional steps were necessary to get five methoxy groups on a styrene unit (see SI for specific synthesis details). Free radical polymerization of these monomers with styrene (S), followed by deprotection of the methoxy groups, yielded non-canonical phenolic copolymers (Supplementary Figs. 4 and 5). Hereafter, poly(2,3,4,5-tetrahydroxystyrene) and poly(2,3,4,5,6-pentahydroxystyrene) are denoted as P4HS and P5HS, respectively, so that the number of hydroxyl groups is highlighted (Fig. 1a). Similarly, P1HS, P2HS, and P3HS-based copolymers were synthesized (Supplementary Figs. 6–8) as reference compounds^17,22,23^ (see SI for synthetic details).

The first variable we optimized for underwater adhesion was the molecular weight (*M*_n_) of the copolymers (Fig. 1b). After synthesis, the copolymer solutions (300 mg/mL in CHCl_3_/MeOH = 9:1 v/v) were individually deposited onto a metal rod underwater and overlapped with another rod for different waiting time. The adhesion strength when the jointed rods were pulled apart was measured by a load cell (see Methods and SI for adhesion test details). With increasing *M*_n_ from 20 to 40 kDa, the adhesion strength increased from ~3.4 MPa to ~6.0 MPa without significant improvements above 40 kDa for both P(4HS*-co*-S) and P(5HS*-co*-S). This plateau is likely because the chain entanglement contributes to the bond strength when the molecular weight is 20–40 kDa, as the entanglement molecular weight (*M*_e_) of PS is ~17 kDa^24^, and *M*_n_ of ~70–80 kDa was used for further studies (Supplementary Table 1). We used 300 mg/mL for adhesion to compare our non-canonical phenolic polymers with canonical catechol and gallol polymers. The viscosity of 4HS and 5HS polymers was measured at different *M*_n_, and the critical concentration c* for the entanglement of polymer chains was estimated (Supplementary Fig. 9). Even the c* of 4HS and 5HS polymers with low *M*_n_ were ~30 mg/mL, suggesting that our concentration of 300 mg/mL was sufficient for polymer chain-entanglement to improve the cohesive strength of the polymers.

Although the adhesion strengths of both non-canonical phenolic polymers were similar, P(5HS*-co*-S) had a narrower distribution of adhesion strengths than P(4HS*-co*-S) and reached 1.4 MPa after only 10 s, which is 5–100 times stronger than commercial products and the literature^4^ (Fig. 1c). After only 2 min of waiting, a P(5HS*-co*-S) bond could lift a 10 kg weight (Supplementary Fig. 10) and after 72 h, a remarkable bond strength plateau (~7.0 MPa) was reached for both P(4HS*-co*-S) and P(5HS*-co*-S) (Fig. 1d). Importantly, these adhesion strengths were maintained after at least 1 month, which validates the potential for long-term usage.

### Phenolic polymers with different substitution patterns of hydroxyl groups

**Fig. 2:**
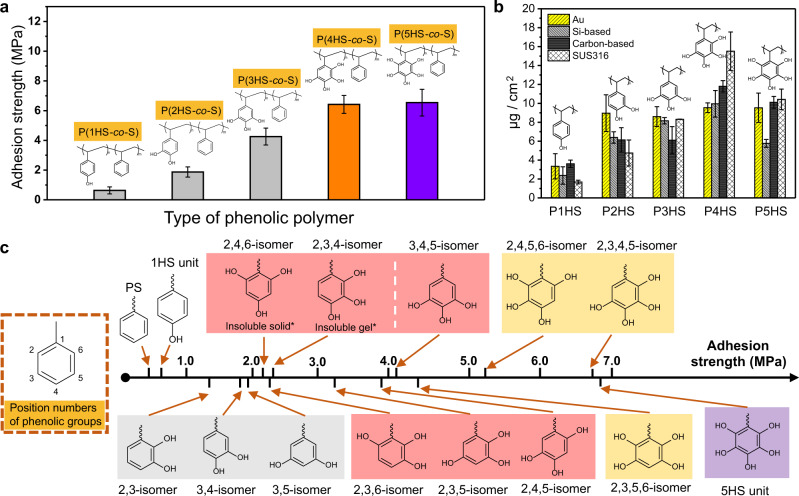
Underwater adhesion of phenolic polymers with various phenol-substitution patterns. **a** Adhesion strengths of P(1HS*-co*-S) to P(5HS*-co*-S) with ~10% of phenolic units at their optimized *M*_n_ (~80 kDa). The results represent the mean ±  s.d. (*n* = 5). **b** Adsorbed quantities of phenolic homopolymers on various QCM chips. The results represent the mean ± s.d. (*n* = 5). **c** Adhesion strength of phenolic copolymers with defined numbers and positions of hydroxyl groups. *For, 2,4,6- and 2,3,4-isomers, the copolymers with 2.5% phenolic content were tested because the copolymers with 10% phenolic content were insoluble.

The remarkable underwater adhesion of P(4HS*-co*-S) and P(5HS*-co*-S) demonstrated that moving beyond the confines of biology, which is limited to 3 hydroxyl (gallol) groups, is crucial for improving the performance of underwater adhesives. A detailed investigation of copolymers with different numbers of phenolic hydroxyl groups demonstrated a fairly linear increase in adhesion strength (i.e., 1HS < 2HS < 3HS < 4HS ≈ 5HS) with ~1–2 MPa increases for each hydroxyl group up to 4 (Fig. 2a). Similar trends were obtained for the adsorption of homopolymers of P1HS to P5HS using a quartz crystal microbalance (QCM) with chips of different compositions (Fig. 2b). The adsorption quantity increased as the number of hydroxyl groups increased, with a plateau between P4HS and P5HS (i.e., 1HS < 2HS < 3HS < 4HS ≈ 5HS). Because of the high affinity of phenolic compounds to Au, the polymer solutions had to be diluted 10 times and incubated for a shorter incubation time (1 min) to see the same trend on Au surfaces (Supplementary Fig. 11). The adsorption of the copolymers was also tested on Au surfaces, and a similar trend to the homopolymers was seen (Fig. 2b, Supplementary Fig. 12). These results demonstrate a strong interaction between various substrates and P4HS or P5HS. The adsorption behavior of individual phenolic units (1HS to 5HS) to a Fe surface was examined by MD-simulation, which demonstrated that 4HS and 5HS had higher adsorption energies that likely accounted for their higher adhesion strengths (Supplementary Fig. 13). The homopolymers (P1HS to P5HS) were also tested for their dry adhesion with ~2.0 kDa *M*_n_ (Supplementary Fig. 14), and P4HS and P5HS had stronger adhesion strengths than the other homopolymers, confirming the importance of the non-canonical phenolic units.

To investigate the effect of the hydroxyl groups’ position, isomers of non-adjacent hydroxyl groups, 2-adjacent hydroxyl groups, and 3-adjacent hydroxyl groups of 2HS, 3HS, 4HS monomers were synthesized (Supplementary Figs. 15–23). All P4HS positions had higher adhesion strengths than all P3HS positions, which in turn had higher adhesion strengths than all P2HS positions (Fig. 2c). Among the P2HS isomers, 3,5-2HS had the highest underwater adhesion strength (1.9 MPa), while the well-known mussel-inspired catechol polymer (3,4-2HS) had slightly lower strength (1.8 MPa) and 2,3-2HS had the lowest bond strength (1.5 MPa). This indicated that the catechol unit is not necessarily the best motif choice for underwater adhesion, even when simply choosing between 2HS polymers.

The hydroxyl groups on positions 2 and 6 seemed unbeneficial for strong adhesion (Fig. 2c) as both 2,3-2HS and 3,4-2HS had *ortho*-dihydroxy styrene structures, but 3,4-2HS still had higher adhesion strength than 2,3-2HS. Likely, the interaction between hydroxyl groups at position 2 or 6 and the substrate surfaces or hydroxyl groups of other polymer chains may be interrupted by the polymer backbone. For the P3HS isomers, 2,3,6-3HS showed the weakest adhesion strength (2.2 MPa), which can be attributed to significant steric-hindrance from both the 2 and 6 positioned hydroxyl groups. Both P4HS isomers and P5HS had to have hydroxyls at the 2 and 6 positions, and this might explain why the adhesion strength did not significantly increase from 2,3,4,5-4HS to P5HS (Fig. 1). Moreover, the 2,3,5,6-4HS isomer had a weaker adhesion strength (4.3 MPa) than the other P4HS isomers, likely due to the enhanced steric hindrance occurring from the pair of adjacent hydroxyl groups next to the polymer backbone. These results collectively confirmed that the higher number of hydroxyl groups in the non-canonical phenolic polymers were superior to bio-inspired polymers for both adsorption and adhesion and that the hydroxyl groups at positions 2 and 6 were not as effective as the ones at positions 3, 4, and 5.

### Spatial confinement in non-canonical phenolic copolymer adhesives

**Fig. 3:**
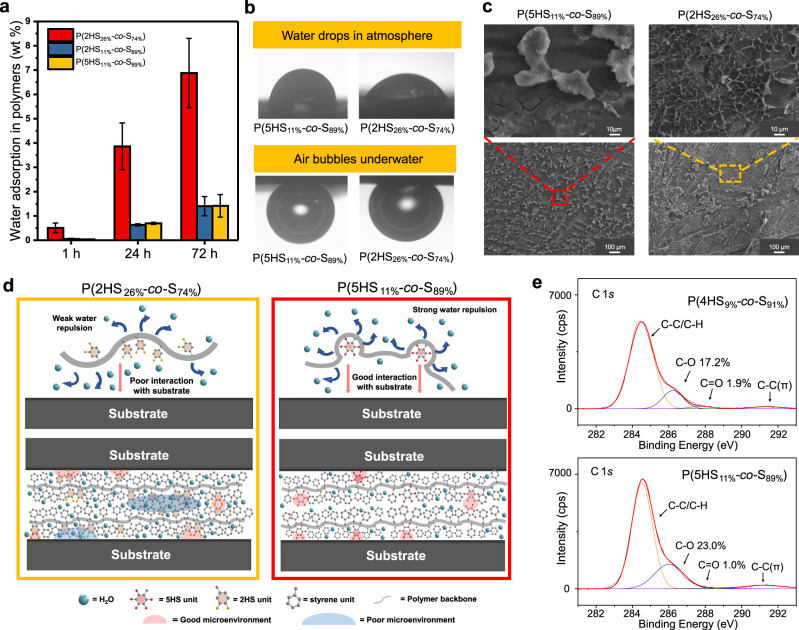
Spatially confined phenolic groups reduce water uptake. **a** Comparison between water uptake of P(2HS_26%_*-co*-S_74%_), P(2HS_11%_*-co*-S_89%_) and P(5HS_11%_*-co*-S_89%_) via QCM. The results represent the mean ±  s.d. (*n* = 3). **b** Surface contact angle images of fractured aluminum surfaces containing P(5HS_11%_*-co*-S_89%_) and P(2HS_26%_*-co*-S_74%_) in air and in water. **c** SEM images of fractured aluminum surfaces containing P(5HS_11%_*-co*-S_89%_) (left, red) and P(2HS_26%_*-co*-S_74%_) (right, yellow). **d** Proposed mechanism of water-shielding at the interface between the substrate and polymers. **e** XPS spectra of fractured aluminum surfaces containing P(4HS_9%_*-co*-S_91%_) and P(5HS_11%_*-co*-S_89%_).

The composition of the copolymers was investigated next as it has previously been reported that catechol copolymers with a composition of P(2HS_25%_*-co*-S_75%_) have the best underwater adhesion (~3.0 MPa)^14^. We also synthesized a polymer with a similar -OH composition, namely P(2HS_26%_*-co*-S_74%_) and compared it to P(5HS_11%_*-co*-S_89%_), as these two polymers have almost the same total amount of hydroxyl groups (namely, 2 × 26% and 5 × 11%). Interestingly, P(5HS_11%_*-co*-S_89%_) had even better adhesion than P(2HS_26%_*-co*-S_74%_) after 72 h (6.9 MPa vs. 3.1 MPa), which can be attributed to the arrangement of hydroxyl groups on the polymer. To elucidate whether the styrene composition played a role in underwater adhesion, the two polymers were separately coated onto Au QCM chips via dip*-*coating. Coated chips were then washed and dipped into DI-water where P(2HS_26%_*-co*-S_74%_) absorbed roughly five times more water than P(5HS_11%_*-co*-S_89%_) per μg of polymer adsorbed (i.e., 6.9% and 1.4% of water by polymer weight, Fig. 3a). Moreover, it should be noted that the amount of P(2HS_26%_*-co*-S_74%_) deposited onto the Au QCM chips was less than that of P(5HS_11%_*-co*-S_89%_) (10 and 14 μg/cm^2^), clearly confirming that P(5HS_11%_*-co*-S_89%_) adsorbed more to the substrate, was more hydrophobic, and could repel water better. Static contact angle (SCA) measurements were carried out on aluminum substrates coated by these two polymers, where P(5HS_11%_*-co*-S_89%_) was more hydrophobic than P(2HS_26%_*-co*-S_74%_) in air (Fig. 3b and Supplementary Table 2), likely due to the higher styrene content in 5HS polymer. On the other hand, the air bubble contact angles were nearly the same under water, likely because the two polymers had similar amounts of hydroxyl groups. To further support this, we also studied P(2HS_11%_*-co*-S_89%_) with a similar styrene content to P(5HS_11%_*-co*-S_89%_) (Fig. 3a, Supplementary Fig. 24, and Supplementary Table 2). The amount of water adsorbed and the hydrophobicity of a P(2HS_11%_*-co*-S_89%_)*-*coated surface were both similar to those of a P(5HS_11%_*-co*-S_89%_)*-*coated surface, indicating that the higher styrene content can help the copolymers repel water.

We also investigated the morphologies of these polymers after adhesion and fracture using scanning electron microscopy (SEM), and the surface of P(5HS_11%_*-co*-S_89%_) appeared to be more homogenous than that of P(2HS_26%_*-co*-S_74%_) (Fig. 3c). For example, the sponge-like structure seen in the fracture of P(2HS_26%_*-co*-S_74%_) is similar to what is seen for catechol adhesives after direct exposure to water and subsequent solidification^16^, indicating that P(2HS_26%_*-co*-S_74%_) had more contact with water than P(5HS_11%_*-co*-S_89%_) during the waiting period, which negatively impacts underwater adhesion. For P(2HS_11%_*-co*-S_89%_), adhesive failure was confirmed, and a sponge-like morphology was observed on the tested surfaces (Supplementary Fig. 24). Collectively, these results demonstrated the polymer composition, namely the amount of hydrophobic styrene residues and the number and positioning of the phenolic groups, plays an important role in underwater adhesion.

The comparison between P(2HS*-co*-S) and P(5HS*-co*-S) with similar total hydroxyl contents gave mechanistic insight into the microenvironment at the substrate-polymer interface with parallels to trends seen in biology (Fig. 3d). For example, adhesive mussel proteins have high proportions of hydrophobic residues (often >70%) with catechol groups are often clustered and spatially confined (2 or 3 adjacent catechol). The hydrophobic portions of the protein can thus simultaneously prevent catechol oxidation while inducing hydrophobic interactions for wet adhesion^25,26^. Similarly, the non-canonical phenolic groups in P(4HS*-co*-S) and P(5HS*-co*-S) are equivalent to two to three spatially confined catechol groups and thus can generate better hydrophobic microenvironments than natural or synthetic catechol polymers. Over 90% of the polymer can be hydrophobic residues while maintaining similar total amounts of hydroxyl groups to catechol polymers. This spatial confinement can be difficult to achieve synthetically with bio-inspired phenolic units and provides a reasonable explanation for why the underwater adhesion strength of 2HS and 3HS adhesives decreases when the phenolic content increases in bio-inspired polymers^14,20^. Excessive phenolic units in bio-inspired polymers not only break the balance of phenolic units with themselves and with substrate surfaces but also change the solubility of the polymer chains and generate hydrophilic microenvironments with more bound water molecules. These hydrophilic microenvironments not only could form at interfaces, but also in bulk, leading to the deterioration of both adhesive and cohesive interactions. Collectively, this explains the difference between the globular fracture patterns of P(5HS*-co*-S) and the spongy patterns of P(2HS*-co*-S) (Fig. 3c).

The hydroxyl groups of phenolic molecules are known to be oxidized^17,27^, which is why mussel proteins evolved such high concentrations of hydrophobic amino acids, however, no theoretical or experimental information exists on the oxidation of phenolics with 4 and 5-hydroxyl residues per aromatic ring as we are the first to successfully synthesize such polymers. Therefore, ^1^H NMR was used to monitor the oxidation of the non-canonical phenolic polymers by themselves (not as adhesives). The ∼30% of hydroxyls in 4HS units in P(4HS*-co*-S) and ∼20 % in P(5HS*-co*-S) were oxidized after exposure to air for 7 days (Supplementary Fig. 25). Interestingly, even after one month, the oxidation of P(5HS*-co*-S) was still around 20%, meaning a single hydroxyl residue oxidized per 5HS unit, while nearly 60% of the hydroxyl groups in P(4HS*-co*-S) were oxidized, or roughly 2 to 3 per 4HS unit. The oxidation rate of P(5HS*-co*-S) over 28 days matched closely to that of P(2HS*-co*-S), suggesting that the 4 position hydroxyl residue on each 5HS unit oxidized to a quinone, leaving a stable pair of catechol residues (2,3 and 5,6 positions) similar to what is reported in the literature for the oxidation of hexahydroxybenzene^28^. We further monitored the oxidation of the homopolymers in deionized water over 20 days by ultraviolet–visible (UV–Vis) spectroscopy, and over 4 weeks by Fourier-transform infrared (FT-IR) spectroscopy. In the FT-IR spectra, the –C=O peak at around 1650 cm^−1^ represented the oxidation of phenol to quinone. After 4 weeks, the –C=O peak was negligible for P3HS, but observable for P4HS and P5HS (Supplementary Fig. 26). Similarly, phenolics have specific UV–Vis adsorption spectra, and changes can suggest oxidation (Supplementary Fig. 27). Specifically, an increase in the baseline was seen during oxidation and overall the results supported our ^1^H NMR results. Further, the water-shielding hydrophobic chains of the non-canonical phenolic copolymers helped to exclude water and prevent oxidation during underwater adhesion. After waiting for 7 days underwater and then fracturing, X-ray photoelectron spectroscopy (XPS) results demonstrated that only 10.0% and 4.1% of hydroxyl groups in 4HS and 5HS polymers were oxidized, respectively in the C 1 *s* spectra (Fig. 3e). We also changed the photoelectron take-off angle from 90° to 30° to gather information on oxidation at the very surface of the fractured samples. Specifically, we found that 33.3% of the 4HS units and 22.2% of the 5HS units were oxidized to quinone, likely with a majority of that occurring post-fracture due to the discrepancy between the amount of oxidation seen at the upper and lower measurement depths (Supplementary Fig. 28). Moreover, by eye it could be seen that the nearly colorless films post fracture became pink or purple after XPS measurement, suggesting oxidation mainly occurred during the XPS preparation and measurement process. The limited oxidation during setting underwater implies that negligible cross-linking is taking place, as there is no cross-linkable space in the 5HS unit, which again implies a different adhesion and stabilization mechanism from the previously reported oxidation-mediated crosslinked catechol and gallol adhesives^14,17^. Gel permeation chromatography (GPC) was used to characterize the *M*_n_ and PDI of the polymers after adhesion, and no change was seen after 2 or 4 weeks (Supplementary Table 3). This indicated that negligible crosslinking occurred between the units of 4HS or 5HS during adhesion.

### Backbone modification to minimize steric hindrance and promote adhesion

**Fig. 4:**
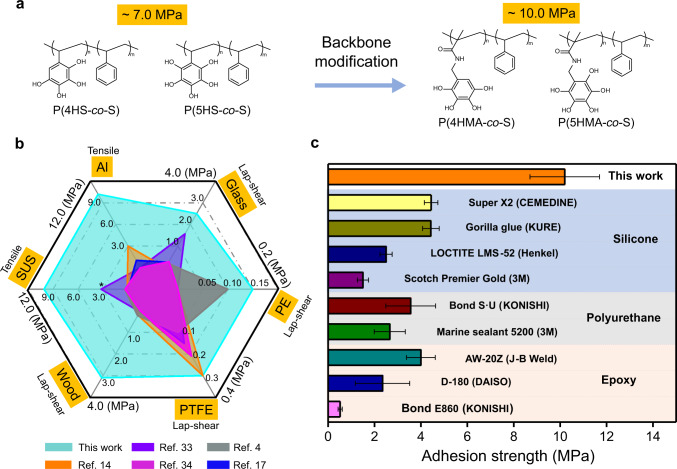
Benchmarking the multi-phenolic adhesive with commercial and literature-reported ones. **a** Change of the backbone structure to methacrylic amide further enhanced the underwater adhesion strength. **b** Comparison of P(4HMA_8%_*-co*-S_92%_) to literature-reported values on various surfaces. **c** Benchmarking of P(4HMA_8%_*-co*-S_92%_) to typical commercial glues on steel substrates underwater. The results represent the mean ± s.d. (*n* = 5). *Results of ref. ^33^ were performed by lap-shear tests.

Cohesive failures were always observed for adhesion tests of P(4HS*-co*-S) and P(5HS*-co*-S). On the other hand, cohesive failures only occurred in polymers with high *M*_n_s in the case of P(3HS*-co*-S) and P(2HS*-co*-S)^29^. This phenomenon indicated that our non-canonical 4HS and 5HS units had a better affinity to the surface of the substrates. Also, our previous results demonstrated that the 2 and 6 hydroxyl positions only minimally contributed towards adhesion. Thus, we further modified the monomer structures of TMS and PMS to enhance the cohesive interaction of polymer adhesives by increasing the spacing between the polymer backbone and the non-canoncial phenolic moieties. Specifically, methacrylamide was selected because of its length, chemical stability, and high cohesive interactions via hydrogen bonds. Two monomers, *N*-(2,3,4,5-tetramethoxybenzyl)methacrylamide (TMA) and *N*-(2,3,4,5,6-pentamethoxybenzyl)methacrylamide (PMA) were synthesized (Supplementary Fig. 29 and 30), copolymerized with styrene to get *M*_n_s of ~80 kDa (Supplementary Table 1), and finally deprotected to obtain P(4HMA_8%_*-co*-S_92%_) and P(5HMA_9%_*-co*-S_91%_) (Fig. 4a, Supplementary Figs. 31 and 32). Homogenous interconnected structures of P(5HMA_9%_*-co*-S_91%_) can be seen after adhesion and fracturing (Supplementary Fig. 33) that were thicker and larger than that of P(5HS_11%_*-co*-S_89%_), suggesting increased cohesion. The underwater adhesion strength was unprecedented at 10.3 MPa for P(4HMA_8%_*-co*-S_92%_) and 9.9 MPa for P(5HMA_9%_*-co*-S_91%_) (Supplementary Fig. 34), with a typical work of adhesion at around 11.8 kJ/m^2^ (Supplementary Fig. 35) after one week of setting time. Further adhesion tests were primarily done with 4HMA, as it has slightly higher adhesion strength. In water, it took one week for the residual solvent to completely diffuse out from the adhesive layer (Supplementary Fig. 36). The fracture of the curve shown in Supplementary Fig. 35 is sharp, indicating that the adhesive was solidified before the test, and we believe it presents the strongest value of hardened underwater adhesives ever reported. Due to the steric hinderance, both 4HMA and 5HMA had relatively low reactivity when copolymerized with styrene, and a higher concentration of monomers (~25–30%) was necessary in the feed to get ~10% composition in the resultant copolymers. The Fineman-Ross method^30^ was used to estimate the reactivity of 4HMA and styrene, and the *r*_4HMA_ was 0.19 and *r*_St_ was 3.0 (Supplementary Fig. 37), meaning that 4HMA monomers prefer to polymerize with styrene, and styrene prefers to self-polymerize.

The crosshead speed used for the tensile test did not have a measurable impact on the adhesion strengths of P(4HS*-co*-S) when measured from 1 to 100 mm/min (Supplementary Fig. 38), further suggesting solidification of the adhesives during underwater incubation. Heavier preload weights have been reported to increase the adhesion strength of phenolic polymers^31^, and increasing the preload weight from 25 g up to 300 g led to increased adhesion strengths for both P(4HS*-co*-S) (7 to ~9 MPa) and P(4HMA*-co*-S) (10 to 11 MPa) (Supplementary Fig. 39). The compression of the adhesive layer is considered to be the reason for the improvement of adhesion strength. The previous report showed that thin adhesive layers are more beneficial than thick ones^32^, and larger quantities did not increase the adhesion strength of our system (Supplementary Fig. 40) as the adhesive layer became thick.

We compared the underwater adhesion strength on different surfaces against literature reports^4,14,17,33,34^ and found that P(4HMA_8%_*-co*-S_92%_) had superior adhesion strengths on every surface (Fig. 4b). Similar to what is seen for mussel-inspired and tannic acid-inspired materials^26,35,36^ our non-canonical phenolic polymer can display multiple interactions including, chelation, electrostatics, hydrogen bonding, and hydrophobic interactions, with different surfaces. For example, they can demonstrate chelation, electrostatic interactions, and hydrogen bonding with metal surfaces, which explains their relatively higher adhesion strength for these substrates in comparison to hydrophobic polymeric surfaces like PTFE and PE where hydrophobic interactions are the primary driving force for adhesion.

The non-canonical phenolic polymers outperformed commercial underwater adhesives on steel, when using 6 mg of commercial adhesives and P(4HMA_8%_*-co*-S_92%_) (Fig. 4c). Collectively, these comparisons confirm that non-canonical phenolic polymers represent the next generation of underwater adhesives.

### Recyclability of non-canonical phenolic polymers

Unlike most commercial glues, including cyanoacrylate, epoxy, silicone, and polyurethane which are irreversible due to crosslinking during setting, our non-canonical phenolic polymers do not rely on covalent crosslinking. We found that they could be removed from substrates simply by rinsing with, or sonicating in, common organic solvents including acetone, tetrahydrofuran, dichloromethane, and ethyl acetate, suggesting that they might be applicable as reversible underwater adhesives. Note that reversible and stimuli-responsive dry adhesives have been created previously using temperature changes^15,33^, UV-irradiation, or specific supramolecular interactions^37^, however, these approaches are challenging to apply with a fully underwater process. Our non-canonical phenolic polymers could be reused simply by re-dissolving the fractured surface for 3 s in 20 μL of solvent (CHCl_3_/MeOH = 9:1), followed by underwater submersion and contact with the other fractured aluminum rod (Supplementary Fig. 41a). Specifically, the bond strength of P(4HMA_8%_*-co*-S_92%_) was still ~2 MPa after the third cycle (Supplementary Fig. 41b), where the decrease in bond strength each cycle could be attributed to the low chain mobility of the polymers during the brief (3 s) re-dissolution step and potential oxidation when the polymers were exposed to the air. Still, even after 4 cycles, adhered aluminum rods could still easily raise an 8-kg-weight (Supplementary Fig. 42). It was also possible to inject the solvent underwater into a small gap in the two separated rods for a completely submerged re-adhesion process.

In summary, we synthesized non-canonical phenolic polymers with 4 and 5 hydroxyl groups on a single phenolic monomer unit to engineer ultrastrong underwater adhesives. The number, amount, and locations of the hydroxyl groups were systematically investigated in regard to absorption to surfaces and adhesion performance, which enabled us to better formulate a mechanism for creating ultrastrong underwater adhesives. The non*-*covalent nature of our adhesives and their negligible oxidation allowed for recyclability and also indicated that their adhesive mechanism is different from bio-inspired adhesives that rely on oxidation-induced crosslinking. Moreover, by spacing the phenolic group farther from the polymer backbone, ultrastrong underwater adhesion (>10 MPa) was achieved that surpasses all commercial and literature underwater adhesives and approaches values seen for commercial dry adhesives. A current challenge in phenolic polymer synthesis, especially in regards to our newly reported non-canonical phenolic polymers, is the use of living polymerization to control the molecular weight and PDI, and this should be a key focus of future research. In our current system, organic solvents were necessary for underwater adhesion, which could limit biomedical application. Organic solvent-free underwater adhesives utilizing coacervation^38^ and emulsion are of great research interest for use with our system in the future. This study establishes a new paradigm for designing underwater adhesives and synthetic phenolic materials with potential benefits to marine and medical fields.

## Methods

### Synthesis of monomers and polymers

The synthesis of phenolic adhesives P(1HS*-co*-S) to P(5HS*-co*-S) was detailed in the supplementary information (SI).

### Characterization of monomers and polymers

The monomers and polymers were characterized by ^1^H NMR, ^13^C NMR, high-resolution mass spectrometry (HRMS), UV-Vis spectroscopy, FT-IR, viscometry, and GPC. Details of characteristic processes and results are found in the SI.

### Preparation and processes for underwater adhesion

For wet adhesion processing, 0.3 g of the phenolic polymer was dissolved in 1 mL mixed solvent (CHCl_3_/MeOH = 9:1) and sonicated for 10 min until the polymers were completely dissolved.

Tensile tests were applied to adhered aluminum (A1050) substrates or steel (SUS304) substrates. A polished aluminum rod or steel rod (JIS-K6849, *D* = 1.27 cm and *h* = 3.80 cm) was completely submerged underwater. The polymer solution (40 μL, 0.3 mg/mL) was deposited on the rod using a pipette and then uniformly painted on its surface underwater. Another piece of the rod was then joined with the polymer-painted rod. A weight (25–300 g) was then placed on the top of the second rod and the joined rods were incubated for a certain period of time for setting. After waiting, the samples were then removed from the water bath and tested on a SHIMAZU AGS-X 10 kN load cell with different crosshead speeds from 1 to 100 mm/min.

For glass, wood, PE, and PTFE surfaces, lap-shear tests were conducted. Flat plates (5 cm × 1 cm × 0.1 cm) were used instead of the rods. The polymer solution (20 μL, 0.3 mg/mL) was deposited onto the surfaces using a pipette underwater before another plate was overlapped with the coated surface. A weight (10 g) was placed on the top of the overlapped surfaces and incubated for a certain period of time for setting. For wood, PE, and PTFE, the overlapped area of the two plates was 1 cm^2^. Because glass is fragile, the overlapped area was set as 0.5 cm^2^.

### Characterization of surfaces containing the adhesives

Surfaces containing the adhesives were characterized by XPS, SCA, SEM, and QCM. Details of the characteristic process and results are found in the SI.

## Supplementary information

Supplementary Information

## Data Availability

The data generated in this study are provided in the Supplementary Information/Source Data file. Source data are provided with this paper.

## Source data

Source Data

## References

[1] An S, Jeon EJ, Jeon J, Cho S-W (2019). A serotonin-modified hyaluronic acid hydrogel for multifunctional hemostatic adhesives inspired by a platelet coagulation mediator. Mater. Horiz..

[2] Ahn BK (2017). Perspectives on Mussel-Inspired Wet Adhesion. J. Am. Chem. Soc..

[3] Stewart RJ, Ransom TC, Hlady V (2011). Natural underwater adhesives. J. Polym. Sci. Part B Polym. Phys..

[4] Cui C (2019). Water-Triggered Hyperbranched Polymer Universal Adhesives: From Strong Underwater Adhesion to Rapid Sealing Hemostasis. Adv. Mater..

[5] Li J (2017). Tough adhesives for diverse wet surfaces. Science.

[6] Lee H, Lee BP, Messersmith PB (2007). A reversible wet/dry adhesive inspired by mussels and geckos. Nature.

[7] Lee H, Scherer NF, Messersmith PB (2006). Single-molecule mechanics of mussel adhesion. Proc. Natl Acad. Sci..

[8] Yu J (2011). Mussel protein adhesion depends on interprotein thiol-mediated redox modulation. Nat. Chem. Biol..

[9] Hwang DS, Zeng H, Lu Q, Israelachvili J, Waite JH (2012). Adhesion mechanism in a DOPA-deficient foot protein from green mussels. Soft Matter.

[10] Mu Y (2017). Contribution of the polarity of mussel-inspired adhesives in the realization of strong underwater bonding. ACS Biomater. Sci. Eng..

[11] Bao Z, Gao M, Sun Y, Nian R, Xian M (2020). The recent progress of tissue adhesives in design strategies, adhesive mechanism and applications. Mater. Sci. Eng. C.

[12] Siebert HM, Wilker JJ (2019). Deriving commercial level adhesive performance from a bio-based mussel mimetic polymer. ACS Sustain. Chem. Eng..

[13] Siebert HM, Wilker JJ (2019). Improving the molecular weight and synthesis of a renewable biomimetic adhesive polymer. Eur. Polym. J..

[14] North MA, Del Grosso CA, Wilker JJ (2017). High strength underwater bonding with polymer mimics of mussel adhesive proteins. ACS Appl. Mater. Interfaces.

[15] Zhao Y (2017). Bio-inspired reversible underwater adhesive. Nat. Commun..

[16] Zhao Q (2016). Underwater contact adhesion and microarchitecture in polyelectrolyte complexes actuated by solvent exchange. Nat. Mater..

[17] Zhan K, Kim C, Sung K, Ejima H, Yoshie N (2017). Tunicate-inspired gallol polymers for underwater adhesive: a comparative study of catechol and gallol. Biomacromolecules.

[18] Shin M, Park E, Lee H (2019). Plant-inspired pyrogallol-containing functional materials. Adv. Funct. Mater..

[19] Kim K (2015). TAPE: a medical adhesive inspired by a ubiquitous compound in plants. Adv. Funct. Mater..

[20] Yu J, Cheng B, Ejima H (2020). Effect of molecular weight and polymer composition on gallol-based underwater adhesive. J. Mater. Chem. B.

[21] Orsini F, Sello G, Fumagalli T (2006). One-pot Wittig reactions in water and in the presence of a surfactant. Synlett.

[22] Cheng B, Ishihara K, Ejima H (2020). Bio-inspired immobilization of low-fouling phospholipid polymers via a simple dipping process: a comparative study of phenol, catechol and gallol as tethering groups. Polym. Chem..

[23] Zhan K, Ejima H, Yoshie N (2016). Antioxidant and adsorption properties of bioinspired phenolic polymers: a comparative study of catechol and gallol. ACS Sustain. Chem. Eng..

[24] Wool RP (1993). Polymer entanglements. Macromolecules.

[25] Cui M, Ren S, Wei S, Sun C, Zhong C (2017). Natural and bio-inspired underwater adhesives: current progress and new perspectives. APL Mater..

[26] Wei W, Yu J, Broomell C, Israelachvili JN, Waite JH (2013). Hydrophobic enhancement of dopa-mediated adhesion in a mussel foot protein. J. Am. Chem. Soc..

[27] Saiz-Poseu J, Mancebo-Aracil J, Nador F, Busqué F, Ruiz-Molina D (2019). The chemistry behind catechol-based adhesion. Angew. Chem. Int. Ed..

[28] Hettegger H, Hosoya T, Rosenau T (2016). Chemistry of the redox series from hexahydroxybenzene to cyclohexanehexaone. Curr. Org. Synth..

[29] Jenkins CL, Meredith HJ, Wilker JJ (2013). Molecular weight effects upon the adhesive bonding of a mussel mimetic polymer. ACS Appl. Mater. Interfaces.

[30] Fineman M, Ross SD (1950). Linear method for determining monomer reactivity ratios in copolymerization. J. Polym. Sci..

[31] Cudjoe E, Herbert KM, Rowan SJ (2018). Strong, rebondable, dynamic cross-linked cellulose nanocrystal polymer nanocomposite adhesives. ACS Appl. Mater. Interfaces.

[32] Rośkowicz M, Godzimirski J, Komorek A, Jasztal M (2021). The effect of adhesive layer thickness on joint static strength. Materials.

[33] Li X, Deng Y, Lai J, Zhao G, Dong S (2020). Tough, long-term, water-resistant, and underwater adhesion of low-molecular-weight supramolecular adhesives. J. Am. Chem. Soc..

[34] Wang Y (2019). A dynamic*-co*upling-reaction-based autonomous self-healing hydrogel with ultra-high stretching and adhesion properties. J. Mater. Chem. B.

[35] Waite JH (2017). Mussel adhesion – essential footwork. J. Exp. Biol..

[36] Guo Z (2021). Tannic acid-based metal phenolic networks for bio-applications: a review. J. Mater. Chem. B.

[37] Heinzmann C, Weder C, de Espinosa LM (2016). Supramolecular polymer adhesives: advanced materials inspired by nature. Chem. Soc. Rev..

[38] Ahn BK (2015). High-performance mussel-inspired adhesives of reduced complexity. Nat. Commun..

