# Supplementary material for: Ultrastrong underwater adhesion on diverse substrates using non-canonical phenolic groups
Source: Nat Commun. 2022 Apr 13;13:1892. doi: 10.1038/s41467-022-29427-w (PMC9008027; doi:10.1038/s41467-022-29427-w)
Supplement: Supplementary file 1 — Supplementary Information [file 41467_2022_29427_MOESM1_ESM.pdf]

Ultrastrong underwater adhesion on diverse substrates using non-canonical phenolic groups

Bohan Cheng, Jinhong Yu, Toma Arisawa, Koki Hayashi, Joseph J. Richardson, Yasushi Shibuta, Hirotaka Ejima

Department of Materials Engineering, School of Engineering, The University of Tokyo, 7-3-1 Hongo, Bunkyo-ku 113-8656, Japan

Correspondence to: ejima@material.t.u-tokyo.ac.jp

Table of Contents

1. Synthesis procedures 3
   1.1 Materials 3
   1.2 Synthesis of 2,3,4,5-tetramethoxystyrene (TMS) 3
   1.3 Synthesis of 2,3,4,5,6-pentamethoxystyrene (PMS) 3
   1.4 Synthesis of *N*-(2,3,4,5-tetramethoxybenzyl)methacrylamide (TMA) 4
   1.5 Synthesis of *N*-(2,3,4,5,6-pentamethoxybenzyl)methacrylamide (PMA) 4
   1.6 Synthesis of 3,5-dimethoxystyrene (isomer of 2HS) 5
   1.7 Synthesis of 2,3-dimethoxystyrene (isomer of 2HS) 5
   1.8 Synthesis of 2,4,6-tris(methoxymethoxy)styrene (isomer of 3HS) 5
   1.9 Synthesis of 2,4,5-trimethoxystyrene (isomer of 3HS) 5
   1.10 Synthesis of 2,3,4-trimethoxystyrene (isomer of 3HS) 6
   1.11 Synthesis of 2,3,5-trimethoxystyrene (isomer of 3HS) 6
   1.12 Synthesis of 2,3,6-trimethoxystyrene (isomer of 3HS) 6
   1.13 Synthesis of 2,3,5,6-tetramethoxystyrene (isomer of 4HS) 6
   1.14 Synthesis of 2,3,4,6-tetramethoxystyrene (isomer of 4HS) 7
   1.15 Synthesis of phenolic polymers 7
2. Experimental processes and characterizations 9
   2.1 Nuclear magnetic resonance (NMR) 9
   2.2 High resolution mass spectrometry (HRMS) 9
   2.3 Gel permeation chromatography (GPC) 9
   2.4 Ultraviolet-visible (UV-Vis) absorption spectroscopy 9
   2.5 Fourier-transform infrared spectroscopy (FT-IR) 9
   2.6 Preparations and processing of the underwater adhesive 9
   2.7 X-ray photoelectron spectroscopy (XPS) 9
   2.8 Static contact angle (SCA) 10
   2.9 Scanning electron microscopy (SEM) 10
   2.10 Quartz crystal microbalance (QCM) 10
   2.11 Viscosity of polymers 10
   2.12 MD-simulation 10
3. Supplementary Tables 12
4. Supplementary Figures 15
5. Supplementary References 57

1. Synthesis procedures

1.1 Materials

2,3,4,5-Tetramethoxytoluene (>97.0%), sodium azide (>98.0%) and triphenylphosphine (PPh_3, >95.0%) were purchased from Tokyo Chemical Industry and used as received. Lithium aluminum hydride ($LiAlH_4$, >95.0%), anhydrous tetrahydrofuran (THF, >99.5%), anhydrous dichloromethane (DCM, >99.5%), *N*-bromosuccinimide (NBS, >98.0%), cyclohexane (>98.0%), formaldehyde (35.0～38.0%), sodium methoxide (>95.0%), copper(I) bromide (CuBr, >95.0%), methanol (>99.5%), anisole (>99.0%), 1,4-dioxane (>99.5%), diethyl-ether (>99.0%), methacryloyl chloride (>97.0%), trimethylamine (TEA, >99.0%), 2,2'-azobis(2-methylpropionitrile) (AIBN, >97.0%), ethyl acetate (EtOAc, >99.0%), methyltriphenylphosphonium bromide (>98.0%), potassium *t*-butoxide (>95.0%), styrene (>99.0%) and other chemicals were purchased from Wako Pure Chemical Industry.

1.2 Synthesis of 2,3,4,5-tetramethoxystyrene (TMS)

A solution of 2,3,4,5-tetramethoxytoluene (12 g, 60 mmol), NBS (10.6 g, 60 mmol) and AIBN (500 mg, 2.5 mmol) in cyclohexane (20 mL) was heated to reflux. After 4.5 h, the solution was filtered to remove insoluble salts and concentrated in vacuo to get a pale yellow oil **(1)** for the next step.

The resulting oil **(1)** and PPh_3 (90 mmol, 23.5 g) were dissolved in 20 mL EtOAc and mixed with 20 mL saturated $NaHCO_3$ (aq.) and 10 mL of 36% formaldehyde solution (aq.). The mixture was stirred at 45 °C overnight. After the reaction, the organic layer was diluted with hexane, collected, dried over magnesium sulfate and concentrated in vacuo. The crude product was subjected to chromatography to get a colorless oil. (2,3,4,5-tetramethoxystyrene, R_f = 0.35, Hexane: EtOAc = 9:1). The total yield was 50.9%. ^{1}H NMR (400 MHz, DMSO-d_6, Supplementary Fig. 1 and 2): (δ, ppm) 6.92 (dd, 1H, J= 17.40 Hz, J'= 10.99 Hz), 6.83 (s, 1H), 5.79 (dd, 1H, J= 17.40 Hz, J'= 0.92 Hz), 5.25 (dd, 1H, J= 10.99 Hz, J'= 0.92 Hz), 3.80 (s, 3H), 3.79 (s, 3H), 3.75 (s, 3H), 3.68 (s, 3H). ^{13}C NMR (400 MHz, Methanol-d_4): (δ, ppm) 149.8, 147.0, 145.3, 142.9, 130.8, 126.1, 113.2, 103.2, 60.6, 60.4, 60.3, 55.4. HRMS (ESI): calculated [M+Na^+] 247.0946 measured [M+Na^+] 247.0939.

1.3 Synthesis of 2,3,4,5,6-pentamethoxystyrene (PMS)

A solution of 2,3,4,5-tetramethoxytoluene (12 g, 60 mmol) and NBS (10.6 g, 60 mmol) in THF (50 mL) was stirred at 0 °C overnight. Then, the organic layer was diluted with diethyl ether, washed with water for 3 times, dried over magnesium sulfate and concentrated in vacuo to obtain a pale yellow oil **(2)** for the next step.

A round bottom flask (500 mL) was charged with oil **(2)**, a solution of sodium methoxide in methanol (5 M, 120 mL), CuBr (1.72 g, 12 mmol) and 3 mL of EtOAc. The mixture was refluxed at 75 °C for 24 h. Then, the mixture was diluted with diethyl ether (100 mL), washed with 1 M H_2SO_4 for 3 times, water for 3 times, dried over magnesium sulfate and concentrated in vacuo to get a colorless oil, 2,3,4,5,6-pentamethoxytoluene **(3)**.

Bromination of **(3)** can be conducted in similar way to the above section, which led to a bromo-substituted 2,3,4,5,6-pentamethoxytoluene **(4)**. One-pot Wittig reaction of **(4)** can be conducted in a similar way to the above section. The crude product after reaction was subjected to chromatography to get a colorless oil. (2,3,4,5,6-pentamethoxystyrene, R_f = 0.45, Hexane: EtOAc = 9:1). The total yield was 60.2%. ^{1}H NMR (400 MHz, $CHCl_3$-d, Supplementary Fig. 1 and 3): (δ, ppm) 6.80 (dd, 1H, J= 17.40 Hz, J'= 10.99 Hz), 6.09 (dd, 1H, J= 17.40 Hz, J'= 0.92 Hz), 5.44 (dd,

1H, J= 10.99 Hz, J'= 0.92 Hz), 3.94 (s, 3H), 3.88 (s, 6H), 3.79 (s, 6H). ^{13}C NMR (400 MHz, Methanol-d$_4$): (δ, ppm) 148.0, 146.8, 143.4, 127.2, 120.2, 117.8, 60.6, 60.4, 59.8. HRMS (ESI): calculated [M+Na$^+$] 277.1051 measured [M+Na$^+$] 277.1046.

1.4 Synthesis of *N*-(2,3,4,5-tetramethoxybenzyl)methacrylamide (TMA)

A round bottom flask (100 mL) was charged with oil **(1)**, sodium azide (4.0 g, 65 mmol) and 40 mL of mix-solvent (THF: DMSO (v/v) = 5:1). The solution was stirred at 40 °C for 1 h. After the reaction, the mixture was diluted with diethyl ether (100 mL), washed with water for 3 times, dried over magnesium sulfate and concentrated in vacuo to get a colorless oil **(5)** for the next step.

At 0 °C, the crude product **(5)** was added dropwise to a suspension of $LiAlH_4$ (2.5 g, 65 mmol) in 120 mL anhydrous THF. After reacting for 2 h at room temperature, the reaction was quenched with 2.5 mL water, 2.5 mL 15% NaOH, and 7.5 mL water, and then stirred for 15 min. After that, 36 g of $MgSO_4$ was added to the mixture, and the mixture was stirred for another 15 min. Finally, the reaction mass was filtered, washed with THF, and evaporated to yield a yellow oil **(6)**.

Under nitrogen atmosphere at 0 °C, methacryloyl chloride (5.0 g, 50 mmol) and oil **(6)** were stirred in 100 mL THF for 5 min. Then, TEA (6.0 g, 60 mmol) was added to the mixture and the reaction solution was brought to room temperature for 24-h-reaction. After the reaction, the organic layer was diluted with diethyl ether, collected, dried over magnesium sulfate and concentrated in vacuo. The crude product was subjected to chromatography to get a colorless oil. (*N*-(2,3,4,5-tetramethoxybenzyl)methacrylamide, R_f = 0.30, Hexane: EtOAc = 5:5). The total yield was 40.0%. ^{1}H NMR (400 MHz, DMSO-d$_6$, Supplementary Fig. 1 and 29): (δ, ppm) 8.34 (-N**H**CO-, 1H), 6.63 (s, 1H), 5.70 (s, 1H), 5.36 (s, 1H), 4.28 (d, 2H), 3.80 (s, 3H), 3.73 (s, 3H), 3.72 (s, 3H), 3.70 (s, 3H), 1.88 (s, 3H). ^{13}C NMR (400 MHz, Methanol-d$_4$): (δ, ppm) 170.0, 149.5, 146.8, 142.2, 140.1, 126.5, 119.2, 106.9, 60.2, 60.1, 60.0, 55.4, 38.1, 17.7. HRMS (ESI): calculated [M+Na$^+$] 318.1317 measured [M+Na$^+$] 318.1317.

1.5 Synthesis of *N*-(2,3,4,5,6-pentamethoxybenzyl)methacrylamide (PMA)

A round bottom flask (100 mL) was charged with oil **(4)**, sodium azide (4.0 g, 65 mmol) and 40 mL of mix-solvent (THF: DMSO (v/v) = 5:1). The solution was stirred at 40 °C for 1 h. After the reaction, the mixture was diluted with diethyl ether (100 mL), washed with water for 3 times, dried over magnesium sulfate and concentrated in vacuo to get a colorless oil **(7)** for the next step.

At 0 °C, the crude product **(7)** was added dropwise to a suspension of $LiAlH_4$ (2.5 g, 65 mmol) in 120 mL anhydrous THF. After reacting for 2 h at room temperature, the reaction was quenched with 2.5 mL water, 2.5 mL 15% NaOH, and 7.5 mL water, and then stirred for 15 min. Then, 36 g of $MgSO_4$ was added to the mixture, and the mixture was stirred for another 15 min. Finally, the reaction mass was filtered, washed with THF, and evaporated to yield a yellow oil **(8)**.

Under nitrogen atmosphere at 0 °C, methacryloyl chloride (5.0 g, 50 mmol) and oil **(8)** were stirred in 100 mL THF for 5 min. Then, TEA (6.0 g, 60 mmol) was added to the mixture and the reaction solution was brought to room temperature for 24 h to react. After the reaction, the organic layer was diluted with diethyl ether, collected, dried over magnesium sulfate and concentrated in vacuo. The crude product was subjected to chromatography to get a colorless oil. (*N*-(2,3,4,5,6-pentamethoxybenzyl)methacrylamide, R_f = 0.45, Hexane: EtOAc = 5:5). The total yield was 52%. ^{1}H NMR (400 MHz, DMSO-d$_6$, Supplementary Fig. 1 and 30): (δ, ppm) 7.82 (-N**H**CO-, 1H), 5.64 (s, 1H), 5.29 (s, 1H), 4.26 (d, 2H), 3.83 (s, 3H), 3.78 (s, 6H), 3.76 (s, 6H), 1.85 (s, 3H). ^{13}C NMR (400 MHz, Methanol-d$_4$): (δ, ppm) 169.3, 148.2, 147.5, 143.1, 140.1, 119.5, 119.2, 60.6, 60.5, 60.3, 33.4, 17.7. HRMS (ESI): calculated [M+Na$^+$] 348.1423 measured [M+Na$^+$] 348.1420.

1.6 Synthesis of 3,5-dimethoxystyrene (isomer of 2HS)

3,5-Dimethoxystyrene was synthesized from commercially available 3,5-dimethoxybenzaldehyde by a conventional Wittig reaction[1]. Briefly, methyltriphenylphosphonium bromide (1.2 *eq.*), potassium *t*-butoxide (1.2 *eq.*) and 3,5-dimethoxybenzaldehyde were mixed in dissolved in 100 mL THF and stirred at 45 °C overnight. After the reaction, the organic layer was diluted with hexane, collected, dried over magnesium sulfate and concentrated in vacuo. The crude product was subjected to chromatography to get a colorless oil. (3,5-dimethoxystyrene, R_f = 0.65, Hexane: EtOAc = 8:2). The total yield was 98%. ^{1}H NMR (400 MHz, DMSO-d_6, Supplementary Fig. 15): (δ, ppm) 6.66 (s, 1H), 6.63 (dd, 1H, J= 17.70 Hz, J'= 10.38 Hz) 6.41 (dd, 1H, J= 17.70 Hz, J'= 0.92 Hz), 5.86 (dd, 1H, J= 10.38 Hz, J'= 0.92 Hz), 3.75 (s, 6H). ^{13}C NMR (400 MHz, Methanol-d_4): (δ, ppm) 161.05, 139.7, 136.7, 113.2, 104.3, 99.8, 54.4. HRMS (ESI): calculated [M+Ag^+] 270.9888 measured [M+Ag^+] 270.9902.

1.7 Synthesis of 2,3-dimethoxystyrene (isomer of 2HS)

2,3-Dimethoxystyrene was synthesized from commercially available 2,3-dimethoxybenzaldehyde by the same procedure mentioned in section 1.6. The crude product was subjected to chromatography to get a colorless oil. (2,3-dimethoxystyrene, R_f = 0.60, Hexane: EtOAc = 8:2). The total yield was 98%. ^{1}H NMR (400 MHz, DMSO-d_6, Supplementary Fig. 16): (δ, ppm) 7.16 (dd, 1H, J= 7.93 Hz, J'= 1.22 Hz), 7.05 (t, 1H, J= 7.93 Hz), 6.99 (dd, 1H, J= 7.93 Hz, J'= 1.22 Hz), 6.94 (dd, 1H, J= 17.70 Hz, J'= 10.99 Hz), 5.81 (dd, 1H, J= 17.70 Hz, J'= 1.22 Hz), 5.31 (dd, 1H, J= 10.99 Hz, J'= 1.22 Hz), 3.79 (s, 3H), 3.68 (s, 3H). ^{13}C NMR (400 MHz, Methanol-d_4): (δ, ppm) 153.0, 146.5, 131.4, 131.0, 124.0, 117.4, 114.0, 111.6, 59.9, 54.9. HRMS (ESI): calculated [M+Ag^+] 270.9888 measured [M+Ag^+] 270.9911.

1.8 Synthesis of 2,4,6-tris(methoxymethoxy)styrene (isomer of 3HS)

2,4,6-Tris(methoxymethoxy)styrene was synthesized from commercially available 2,4,6-trihydroxybenzaldehyde by the method[2] shown in Supplementary Fig. 1. The crude product was subjected to chromatography to get a colorless oil. (2,4,6-tris(methoxymethoxy)styrene, R_f = 0.65, Hexane: EtOAc = 8:2). The total yield was 80%.^{1}H NMR (400 MHz, DMSO-d_6, Supplementary Fig. 1 and 17): (δ, ppm) 6.80 (dd, 1H, J= 20.75 Hz, J'= 12.21 Hz), 6.46 (s, 2H), 5.65 (dd, 1H, J= 20.75 Hz, J'= 3.05 Hz), 5.29 (dd, 1H, J= 12.21 Hz, J'= 3.05 Hz), 5.20 (s, 6H), 5.16 (s, 2H), 3.38 (s, 3H), 3.35 (s, 9H). ^{13}C NMR (400 MHz, Methanol-d_4): (δ, ppm) 157.7, 156.8, 127.1, 115.8, 110.9, 96.8, 94.5, 94.1, 55.2, 55.1. HRMS (ESI): calculated [M+Na^+] 307.1157 measured [M+Na^+] 307.1152.

1.9 Synthesis of 2,4,5-trimethoxystyrene (isomer of 3HS)

2,4,5-Trimethoxystyrene was synthesized from commercially available 2,4,5-trimethoxybenzaldehyde by the same procedure mentioned in section 1.6. The crude product was subjected to chromatography to get a colorless oil. (2,4,5-trimethoxystyrene, R_f = 0.50, Hexane: EtOAc = 8:2). The total yield was 98%. ^{1}H NMR (400 MHz, DMSO-d_6, Supplementary Fig. 18): (δ, ppm) 7.07 (s, 1H), 6.88 (dd, 1H, J= 17.70 Hz, J'= 10.99 Hz), 6.66 (s, 1H), 5.65 (dd, 1H, J= 17.70 Hz, J'= 1.22 Hz), 5.10 (dd, 1H, J= 10.99 Hz, J'= 1.22 Hz), 3.79 (s, 3H), 3.78 (s, 3H), 3.72 (s, 3H). ^{13}C NMR (400 MHz, Methanol-d_4): (δ, ppm) 151.8, 150.0, 143.2, 130.8, 118.4, 110.7, 110.1, 97.7, 56.0, 55.6, 55.2. HRMS (ESI): calculated [M+Na^+] 217.0841 measured [M+Na^+] 217.0845.

1.10 Synthesis of 2,3,4-trimethoxystyrene (isomer of 3HS)

2,3,4-Trimethoxystyrene was synthesized from commercially available 2,3,4-trimethoxybenzaldehyde by the same procedure mentioned in section 1.6. The crude product was subjected to chromatography to get a colorless oil. (2,3,4-trimethoxystyrene, R_f = 0.50, Hexane: EtOAc = 8:2). The total yield was 90%. ^{1}H NMR (400 MHz, DMSO-d_6, Supplementary Fig. 19): (δ, ppm) 7.26 (d, 1H, J= 8.54 Hz), 6.81 (dd, 1H, J= 17.70 Hz, J'= 10.99 Hz), 6.80 (d, 1H, J= 8.54 Hz), 5.68 (dd, 1H, J= 17.70 Hz, J'= 1.22 Hz), 5.18 (dd, 1H, J= 10.99 Hz, J'= 1.22 Hz), 3.79 (s, 3H), 3.74 (s, 3H), 3.73 (s, 3H). ^{13}C NMR (400 MHz, Methanol-d_4): (δ, ppm) 153.4, 151.3, 142.2, 130.9, 124.6, 120.6, 112.0, 107.9, 60.3, 59.9, 55.1. HRMS (ESI): calculated [M+Ag$^+$] 300.9994 measured [M+Ag$^+$] 300.9943.

1.11 Synthesis of 2,3,5-trimethoxystyrene (isomer of 3HS)

2,3,5-Trimethoxystyrene was synthesized by the method shown in Supplementary Fig. 1. The crude product was subjected to chromatography to get a colorless oil. (2,3,5-trimethoxystyrene, R_f = 0.60, Hexane: EtOAc = 8:2). The total yield was 90%. ^{1}H NMR (400 MHz, DMSO-d_6, Supplementary Fig. 1 and 20): (δ, ppm) 6.90 (dd, 1H, J= 17.70 Hz, J'= 10.99 Hz), 6.66 (d, 1H, J= 3.05 Hz), 6.55 (d, 1H, J= 3.05 Hz), 5.84 (dd, 1H, J= 17.70 Hz, J'= 1.22 Hz), 5.31 (dd, 1H, J= 10.99 Hz, J'= 1.22 Hz), 3.78 (s, 3H), 3.75 (s, 3H), 3.61 (s, 3H). ^{13}C NMR (400 MHz, Methanol-d_4): (δ, ppm) 156.4, 153.6, 140.7, 131.2, 130.9, 114.1, 100.0, 99.7, 60.0, 54.9, 54.5. HRMS (ESI): calculated [M+Ag$^+$] 300.9994 measured [M+Ag$^+$] 300.9996.

1.12 Synthesis of 2,3,6-trimethoxystyrene (isomer of 3HS)

2,3,6-Trimethoxystyrene was synthesized by the method shown in Supplementary Fig. 1. The crude product was subjected to chromatography to get a colorless oil. (2,3,6-trimethoxystyrene, R_f = 0.60, Hexane: EtOAc = 8:2). The total yield was 90%. ^{1}H NMR (400 MHz, DMSO-d_6, Supplementary Fig. 1 and 21): (δ, ppm) 6.92 (d, 1H, J= 9.16 Hz), 6.79 (dd, 1H, J= 17.70 Hz, J'= 12.21 Hz), 6.70 (d, 1H, J= 9.16 Hz), 6.05 (dd, 1H, J= 17.70 Hz, J'= 3.05 Hz), 5.42 (dd, 1H, J= 12.21 Hz, J'= 3.05 Hz), 3.75 (s, 3H), 3.74 (s, 3H), 3.67 (s, 3H). ^{13}C NMR (400 MHz, Methanol-d_4): (δ, ppm) 152.7, 148.0, 147.1, 127.4, 120.3, 118.3, 111.6, 106.0, 59.2, 55.4, 54.9. HRMS (ESI): calculated [M+Ag$^+$] 300.9994 measured [M+Ag$^+$] 300.9977.

1.13 Synthesis of 2,3,5,6-tetramethoxystyrene (isomer of 4HS)

The synthesis of the precursor of 4HS isomer, 2,3,5,6-tetramethoxytoluene is illustrated in Supplementary Fig. 1. Briefly, to an ice-cold solution of 2-methylresorcinol (9.6 g, 78 mmol, 1.0 *eq.*) in 90% acetic acid (100 mL), bromine (25.0 g, 160 mmol, 2.0 *eq.*) was added dropwise over 15 min. After stirring at room temperature for 1 h, the reaction mixture was poured on cold H_2O (500 mL). The colorless precipitate was filtered off and washed excessively with water to provide 20.0 g (90%) of a colorless solid (4,6-dibromo-2-methylresorcinol), which was used without further purification.

A round bottom flask (250 mL) was charged with a solution of the white solid gained in the previous last step in DMF, 25 mL of CH_3I, and 20 g of K_2CO_3. The mixture was reacted at 45 °C for 24 h. Then, the mixture was diluted with diethyl ether (100 mL), washed with 1 M $NaHCO_3$ for 3 times, water for 3 times, dried over magnesium sulfate and concentrated in vacuo to get a yellow solid, 3,5-dibromo-2,6-dimethoxytoluene (24.0 g, 98%).

A round bottom flask (500 mL) was charged with 30 mmol of 3,5-dibromo-2,6-dimethoxytoluene, a solution of sodium methoxide in methanol (5 M, 120 mL), CuBr (1.72 g, 12

mmol) and 3 mL of EtOAc. The mixture was refluxed at 75 °C for 24 h. Then, the mixture was diluted with diethyl ether (100 mL), washed with 1 M H_2SO_4 for 3 times, water for 3 times, dried over magnesium sulfate and purified by chromatography (R_f = 0.30, Hexane: EtOAc = 9:1) to get a white solid, 2,3,5,6-tetramethoxytoluene.

2,3,5,6-tetramethoxytoluene was then reacted like the 4HS and 5HS monomers mentioned above. After bromination on its methyl group, one-pot Wittig was conducted, and the crude product was subjected to chromatography to get colorless oil. (2,3,5,6-tetramethoxystyrene, R_f = 0.1, Hexane: EtOAc = 20:1). The total yield was 34%. ^{1}H NMR (400 MHz, DMSO-d_6, Supplementary Fig. 1 and 22): (δ, ppm) 6.75 (dd, 1H, J= 18.31 Hz, J'= 12.22 Hz), 6.72 (s, 1H), 6.07 (dd, 1H, J= 18.31 Hz, J'= 2.44 Hz), 5.46 (dd, 1H, J= 12.22 Hz, J'= 2.44 Hz), 3.80 (s, 6H), 3.60 (s, 6H). ^{13}C NMR (400 MHz, DMSO-d_6): (δ, ppm) 149.3, 140.7, 128.4, 124.5, 120.2, 99.6 60.3, 56.6. HRMS (ESI): calculated [M+Ag^+] 331.0099 measured [M+Ag^+] 331.0099.

1.14 Synthesis of 2,3,4,6-tetramethoxystyrene (isomer of 4HS)

The synthesis of the precursor of 4HS isomer, 2,3,4,6-tetramethoxytoluene is illustrated in Supplementary Fig. 1. To an ice-cold solution of 2-methoxy-5-methylphenol (5 g, 36 mmol, 1.0 *eq.*) in 90% acetic acid (100 mL), bromine (12.0 g, 78 mmol, 2.0 *eq.*) was added dropwise over 15 min. After stirring at room temperature for 1 h, the reaction mixture was poured on cold H_2O (500 mL). The colorless precipitate was filtered off and washed excessively with water to provide 10.0 g (97%) of a pale-yellow solid (2,4-dibromo-6-methoxy-3-methylphenol), which was used without further purification.

A round bottom flask (250 mL) was charged with a solution of the white solid gained in last step in DMF, 12 mL of CH_3I, and 20 g of K_2CO_3. The mixture was reacted at 45 °C for 24 h. Then, the mixture was diluted with diethyl ether (100 mL), washed with 1 M $NaHCO_3$ for 3 times, water for 3 times, dried over magnesium sulfate and concentrated in vacuo to get a yellow solid, 2,6-dibromo-3,4-dimethoxytoluene (9.6 g, 90%).

A round bottom flask (500 mL) was charged with 30 mmol of 2,6-dibromo-3,4-dimethoxytoluene, solution of sodium methoxide in methanol (5 M, 120 mL), CuBr (1.72 g, 12 mmol) and 3 mL of EtOAc. The mixture was refluxed at 75 °C for 24 h. Then, the mixture was diluted with diethyl ether (100 mL), washed with 1 M H_2SO_4 for 3 times, water for 3 times, dried over magnesium sulfate and purified by a column (R_f = 0.40, Hexane: EtOAc = 9:1) to get a colorless oil, 2,3,4,6-tetramethoxytoluene.

2,3,4,6-tetramethoxytoluene was then reacted like 4HS and 5HS monomers mentioned above. After bromination on its methyl group, one-pot Wittig was conducted, and the crude product was subjected to chromatography to get a colorless oil. (2,3,4,6-tetramethoxystyrene, R_f = 0.15, Hexane: EtOAc = 20:1). The total yield was 40%. ^{1}H NMR (400 MHz, DMSO-d_6, Supplementary Fig. 1 and 23): (δ, ppm) 6.71 (dd, 1H, J= 18.31 Hz, J'= 12.22 Hz), 6.49 (s, 1H), 5.92 (dd, 1H, J= 18.31 Hz, J'= 3.05 Hz), 5.25 (dd, 1H, J= 12.22 Hz, J'= 3.05 Hz), 3.83 (s, 3H), 3.80 (s, 3H), 3.71 (s, 3H) 3.67 (s, 3H). ^{13}C NMR (400 MHz, DMSO-d_6): (δ, ppm) 154.8, 152.9, 152.6, 136.2, 127.2, 115.8, 112.7, 92.7, 60.1, 59.8, 55.1, 55.0. HRMS (ESI): calculated [M+Ag^+] 331.0099 measured [M+Ag^+] 331.0098.

1.15 Synthesis of phenolic polymers

In a test tube, precursor monomers (with 1~5 -OCH_3 groups), styrene, and initiator AIBN were dissolved in 2 mL degassed anisole (1,4-dioxane for the polymerization of PMA and TMA). Free radical polymerization was conducted at 95 °C for 48 h under nitrogen atmosphere. After

polymerization, the mixture was then precipitated in hexane. The powder was concentrated in vacuo at 40 °C.

Phenolic polymers were obtained from their protected forms by BBr_3 treatment. Briefly, 0.5 g polymer was dissolved in 20 mL of dichloromethane in a 100 mL round bottomed flask equipped with a magnetic stir bar. The flask was sealed after the solution was purged with nitrogen for 15 min. The solution was cooled to 0 °C and stirred for 10 min, then 1 M BBr_3 solution (15 mL, 15 mmol) was added dropwise over a period of 10 min. After 30 min, the mixture was warmed to room temperature and then stirred overnight. A large amount of 10% HCl aqueous solution was added to this reaction mixture, and after stirring for 2 h, the resulting precipitate was collected by filtration and dried in vacuo at 40 °C for 3 hours for use. (Supplementary Figs. 4–8, 31 and 32)

2. Experimental processes and characterizations

2.1 Nuclear magnetic resonance (NMR)

The polymer composition was determined by ^{1}H NMR (400 MHz) and ^{13}C NMR (400 MHz) analysis using NMR spectrometers (JEOL-ECS400, JEOL, Japan) in $CDCl_3$, Methanol-d_4, or DMSO-d_6. Each sample was scanned 32 times for ^{1}H NMR and 1024 times for ^{13}C NMR.

2.2 High resolution mass spectrometry (HRMS)

Monomers were characterized by ESI-TOF MS (micrOTOF-II, BRUKER, USA) with methanol as eluent.

2.3 Gel permeation chromatography (GPC)

The number-average molecular weight (M_n) and polydispersity index (PDI) were both measured by GPC (Prominence-i GPC System, SHIMAZU, Japan) using THF or DMF (with 10 mM of LiCl) as the eluent. The calibration curve was prepared using polystyrene as a standard.

2.4 Ultraviolet-visible (UV-Vis) absorption spectroscopy

For the oxidation study, 3 mL of polymer solution (0.003 mmol on monomer unit basis in methanol) in a quartz cuvette were subjected to UV-Vis Spectrophotometry (NanoDrop One^{C}, Thermo Scientific, USA) and the data was recorded every 5 days.

2.5 Fourier-transform infrared spectroscopy (FT-IR)

For the oxidation studies, dry polymer was mixed with KBr crystal and moulded to a thin film for FT-IR characterization (FV-6300 FT-IR Spectrophotometer, JASCO, Japan). The data was recorded every week. Polymers were stored in DI-water and freeze-dried before the measurements.

2.6 Preparations and processing of the underwater adhesive

For wet adhesion processing, 0.3 g of the phenolic polymer was dissolved in 1 mL mixed solvent ($CHCl_3$/MeOH = 9:1) and sonicated for 10 min until the polymers were completely dissolved.

Tensile tests were applied to adhered aluminum (A1050) substrates or steel (SUS304) substrates. A polished aluminum rod or steel rod (JIS-K6849, D=1.27 cm and h=3.80 cm) was completely submerged underwater. The polymer solution (40 μL, 0.3 mg/mL) was deposited on the rod using a pipette and then uniformly painted on its surface underwater. Another piece of rod was then joined with the polymer-painted rod. A weight (25–300 g) was then placed on the top of the second rod and the joined rods were incubated for a certain period of time for setting. After waiting, the samples were then removed from the water bath and tested on a SHIMAZU AGS-X 10 kN load cell with different crosshead speeds from 1 mm/min to 100 mm/min.

For glass, wood, PE, and PTFE surfaces, lap-shear tests were conducted. Flat plates (5 cm × 1 cm × 0.1 cm) were used instead of the rods. The polymer solution (20 μL, 0.3 mg/mL) was deposited onto the surfaces using a pipette underwater before another plate was overlapped with the coated surface. A weight (10 g) was placed on the top of the overlapped surfaces and incubated for a certain period of time for setting. For wood, PE and PTFE, the overlapped area of the two plates was 1 cm2. Because glass is fragile, the overlapped area was set as 0.5 cm^2.

2.7 X-ray photoelectron spectroscopy (XPS)

The elemental compositions of the fractured polymer surfaces after underwater adhesion were investigated by XPS (AXIS-His, Shimadzu/Kratos Co. Ltd., Kyoto, Japan) with a Mg Kα (12 kV) radiation source at the anode. The photoelectron take-off angle was fixed at 90°, 67.5°, 45° and 30°. All the spectra were referenced to the C 1*s* peak at 284.5 eV of the binding energy.

2.8 Static contact angle (SCA)

The contact angles with water droplets on the substrates in air and air bubbles on the substrates underwater were measured at room temperature using an optical-bench type contact-angle goniometer (Model DM300, Kyowa Interface Science Co., Ltd., Saitama, Japan). The contact angles were directly measured within 10 s. For each substrate, water droplets were put on three different points. For each substrate type, the same measurement was performed on five different specimens.

2.9 Scanning electron microscopy (SEM)

The morphologies of the fractured surfaces of aluminum substrates after adhesion tests were observed using a scanning electron microscope (SEM, SM-200, TOPCON, Tokyo, Japan). Before the observation, the surfaces were sputtered with gold (~200 nm).

2.10 Quartz crystal microbalance (QCM)

QCM sensors with Au-based surfaces (9 MHz, Seiko EG&G) were dipped into polymer solutions (0.5 wt% in $CHCl_3$/MeOH = 9:1 v/v) and incubated for 10 min, followed by 10 min drying in air. The quantities of polymers adsorbed on the surface were determined according to frequency shift recorded by quartz crystal microbalance (QCM-992A, SEIKO EG&G).

2.11 Viscosity of polymers

The melt viscosity and intrinsic viscosity of polymers were measured by microVISC Viscometer (RheoSense Inc, USA). The shear rate was set at 5000 s^{-1} and DMSO was used as solvent. The critical concentration of polymers c^* was calculated as the reciprocal of intrinsic viscosity.

2.12 MD-simulation

Molecular dynamics (MD) simulations were performed using Large-scale Atomic/Molecular Massively Parallel Simulator (LAMMPS)[3]. A ReaxFF force field parametrized by Shin et al[4] was employed as an interatomic potential for Fe/C/H/O interactions. The velocity–Verlet method was used to integrate the classical equation of motion with a time step of 0.1 fs. The Nosé–Hoover thermostat[5,6] was employed to control temperature. A base-centered-cubic (bcc) Fe crystal consisting of 6 × 6 × 3 unit cells (216 atoms) was placed at the bottom of a rectangular cell of 17.2 × 17.2 × 25.8 $Å^3$, where the (001) surface appeared on the surface perpendicular to the z-axis. Periodic boundary conditions are employed for all directions. One molecule of benzene, hydroxybenzene, 1,2-dihydroxybenzene, 1,2,3-hydroxybenzene, 1,2,3,4-tetrahydroxybenzene or 1,2,3,4,5-pendahydroxybenzene was put 5 Å above the Fe surface. Then, the prepared system was relaxed with canonical ensemble at 300 K for 5000 fs to obtain structures of the molecule adsorbed on the metal surface. The calculations were performed 5 times for each molecule with different distributions of initial velocity to gather statistics. Moreover, a system without molecules (i.e., only Fe atoms) and isolated molecules were separately relaxed at 300 K for 5000 fs as the reference states. The adsorption energy E_{ads} is defined as the difference of potential energy of the system

with molecule (E_{total}) and the sum of potential energies of Fe only system (E_{Fe}) and an isolated molecule (E_{mol}) as $E_{ads} = (E_{Fe} + E_{mol}) - E_{total}$. Average potential energy between 4000 fs and 5000 fs was employed as a representative value of the system for all cases.

3. Supplementary Tables

Supplementary Table 1 | Composition, PDI, and M_n of the synthesized polymers used in this study. The results of adhesion strength represent the mean ± s.d. (n = 5).

Phenolic unit (mol%)[a] and unit type (1HS~ 5HS, 4HMA~5HMA)	Styrene (mol%)[a]	PDI[b]	M_n (×10^4)[b]	Adhesion strength (MPa)
10 (1HS)	90	2.0	1.9	0.60 ± 0.25
*10 (1HS)	90	1.9	9.2	0.63 ± 0.23
11 (2HS)	89	2.1	2.3	1.66 ± 0.53
*11 (2HS)	89	2.4	7.5	1.83 ± 0.34
26 (2HS)	74	1.5	7.1	3.10 ± 0.35
9 (2HS, 3,5-isomer)	91	2.0	8.0	1.92 ± 0.45
10 (2HS, 2,3-isomer)	90	1.5	6.7	1.52 ± 0.28
11 (3HS)	89	2.0	2.2	2.13 ± 1.05
*10 (3HS)	90	1.9	8.2	4.25 ± 0.56
10 (3HS, 246-isomer)	90	2.0	7.9	**Not available
2.5 (3HS, 246-isomer)	97.5	1.9	8.0	2.10 ± 0.10
10 (3HS, 245-isomer)	90	2.1	7.6	3.84 ± 0.45
10 (3HS, 234-isomer)	90	1.9	8.2	**Not available
2.5 (3HS, 234-isomer)	97.5	1.9	8.2	2.34 ± 0.35
11 (3HS, 235-isomer)	89	2.0	9.2	3.22 ± 0.12
9 (3HS, 236-isomer)	91	1.7	6.7	2.22 ± 0.23
11 (4HS)	89	2.2	2.1	3.55 ± 0.98
10 (4HS)	90	1.4	4.5	6.24 ± 0.61
12 (4HS)	88	1.7	6.3	6.34 ± 0.73
*11 (4HS)	89	1.8	8.3	6.53 ± 0.90
9 (4HS)	91	2.0	9.8	7.07 ± 0.61
8 (4HS, 2356-isomer)	92	2.2	7.6	4.30 ± 0.42
9 (4HS, 2346-isomer)	91	2.3	7.0	5.12 ± 0.24
11 (5HS)	89	1.9	2.2	3.40 ± 1.00
10 (5HS)	90	2.0	4.3	6.03 ± 0.60
10 (5HS)	90	2.1	6.2	6.24 ± 1.50
*10 (5HS)	90	2.1	8.3	6.42 ± 0.61
11 (5HS)	89	2.0	11.2	6.72 ± 1.02
8 (4HMA)	92	1.8	8.0	10.31 ± 0.89
9 (5HMA)	91	2.2	9.3	9.94 ± 1.20

a: measured by ^{1}H NMR
b: measured by GPC
*: polymer with * stands for the "M_n optimized polymers" used in Fig. 2.
** : The polymer is not soluble in solvent for adhesion test.

Supplementary Table 2 | Static contact angles (SCA) of aluminum substrates covered by the polymer adhesives. The results represent the mean ± s.d. (*n* = 5).

Sample	SCA in dry condition (°)	SCA underwater (°)
P(2HS$_{26\%}$-*co*-S$_{74\%}$)	48.9 ± 6.4	29.1 ± 2.4
P(2HS$_{11\%}$-*co*-S$_{89\%}$)	62.5 ± 2.5	29.7 ± 3.0
P(5HS$_{11\%}$-*co*-S$_{89\%}$)	69.3 ± 6.3	32.5± 4.4

Supplementary Table 3 | M_n and PDI of the polymers after different setting time.

Sample (waiting time)	PDI	M_n ($\times 10^4$)
P(4HS$_{9\%}$-*co*-S$_{91\%}$) (0 week)	1.8	9.8
P(4HS$_{9\%}$-*co*-S$_{91\%}$) (2 week)	1.8	9.7
P(4HS$_{9\%}$-*co*-S$_{91\%}$) (4 week)	1.8	9.8
P(5HS$_{11\%}$-*co*-S$_{89\%}$) (0 week)	2.0	11.2
P(5HS$_{11\%}$-*co*-S$_{89\%}$) (2 week)	2.0	11.3
P(5HS$_{11\%}$-*co*-S$_{89\%}$) (4 week)	2.0	11.3

4. Supplementary Figures

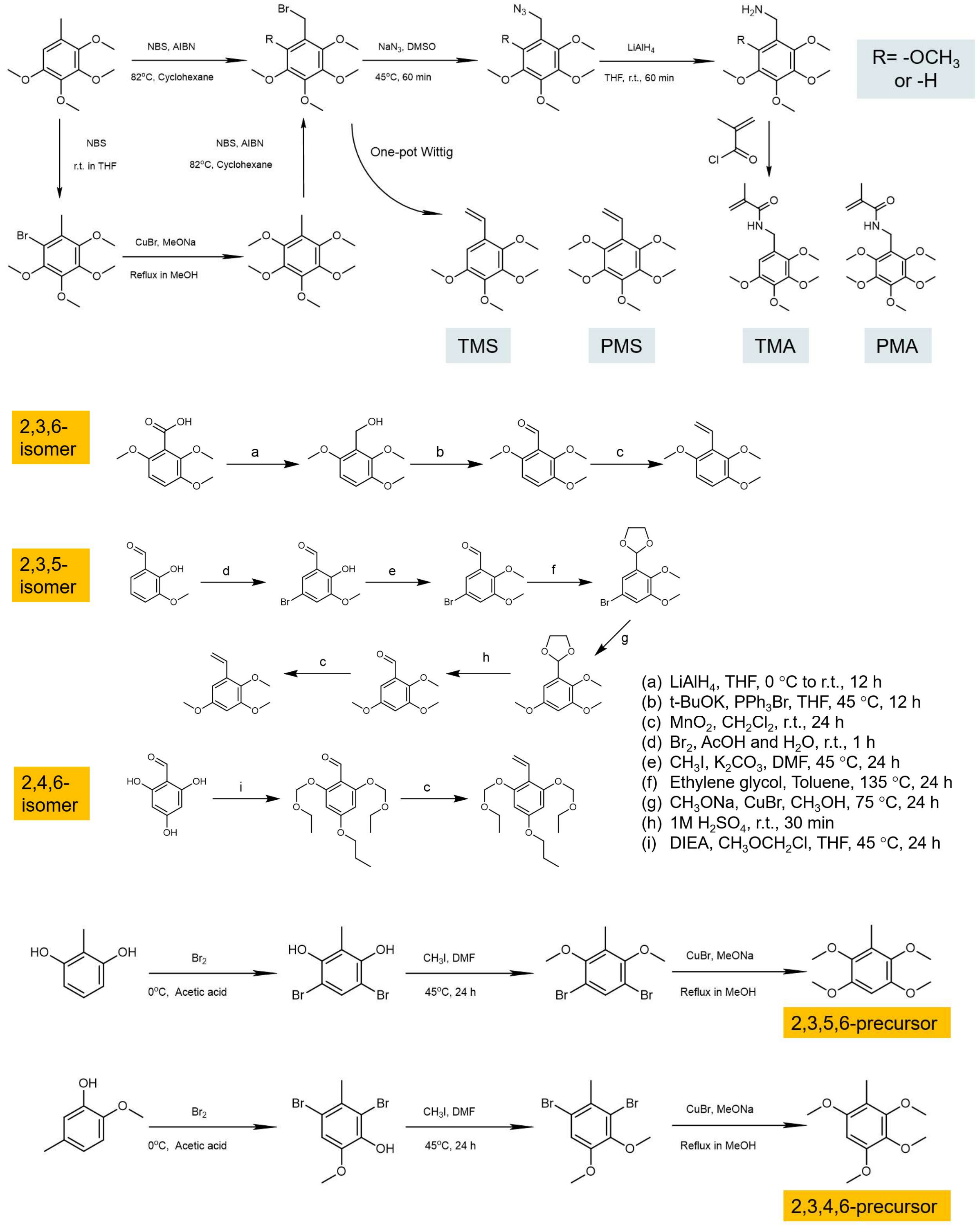

Supplementary Fig. 1 | Synthetic routes for monomers and precursors.

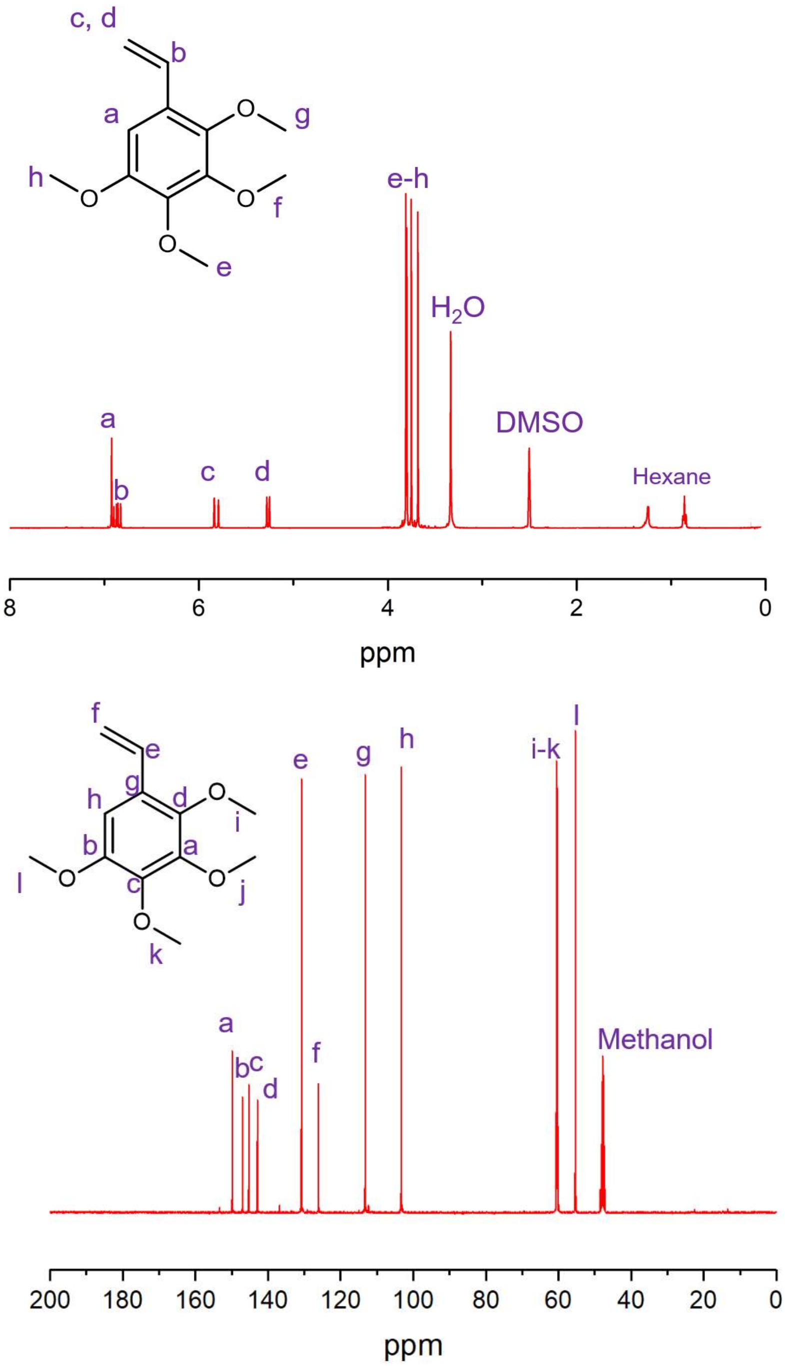

Supplementary Fig. 2 | ^{1}H NMR spectrum of TMS (400 MHz, DMSO-d$_6$) and ^{13}C NMR spectrum of TMS (400 MHz, Methanol-d$_4$).

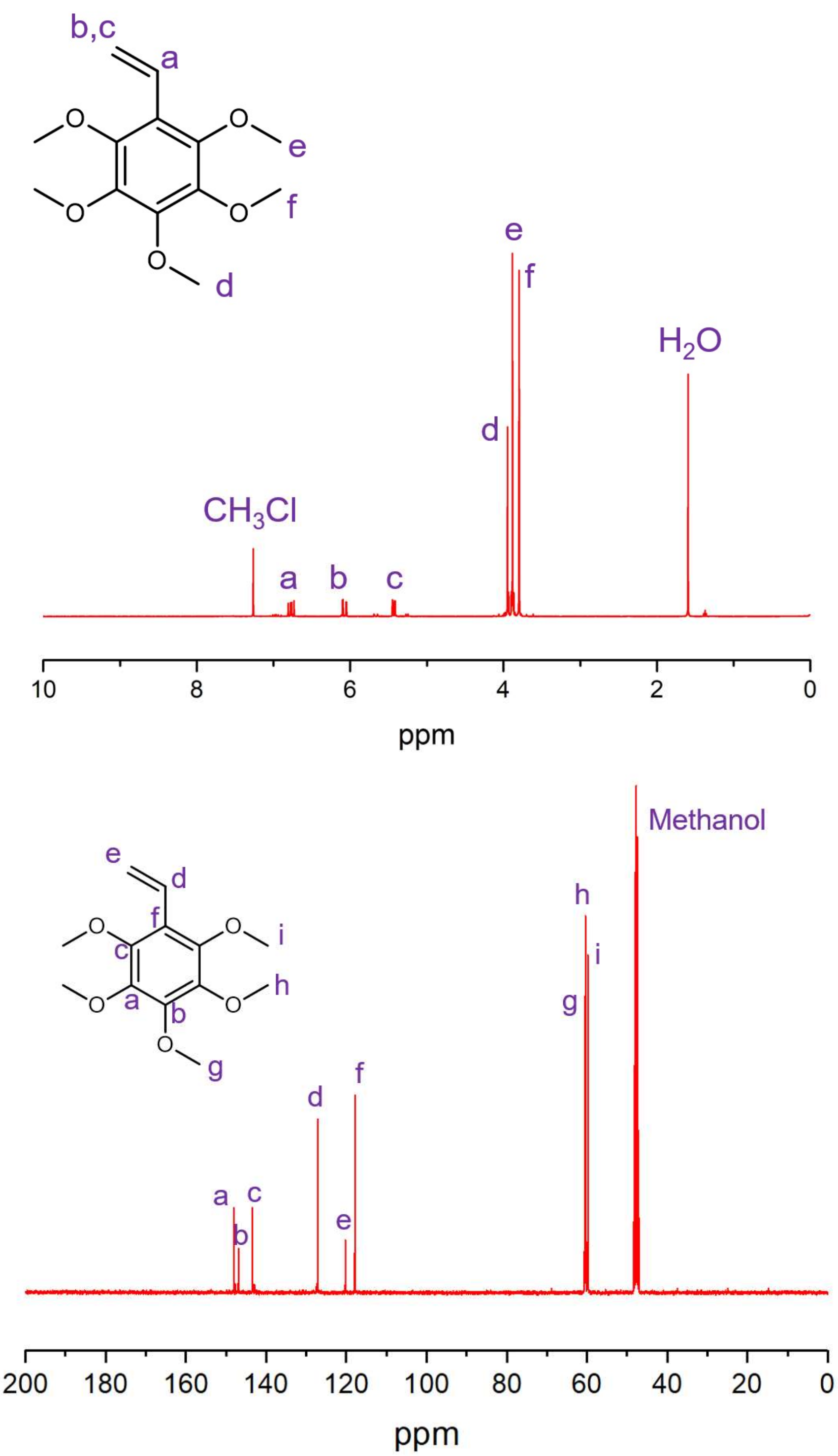

Supplementary Fig. 3 | ^{1}H NMR spectrum of PMS (400 MHz, $CDCl_3$) and ^{13}C NMR spectrum of PMS (400 MHz, Methanol-d_4).

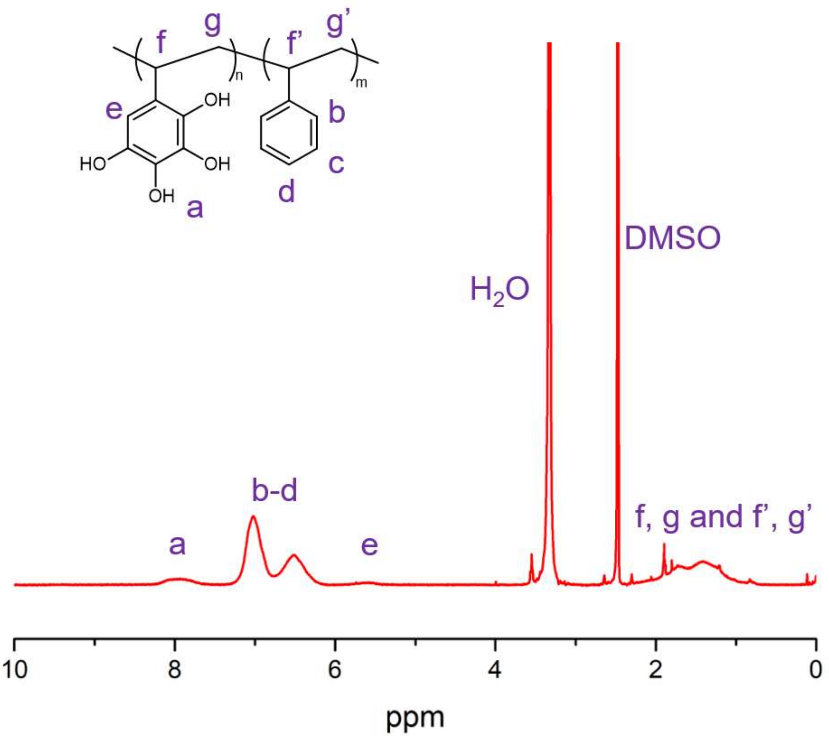

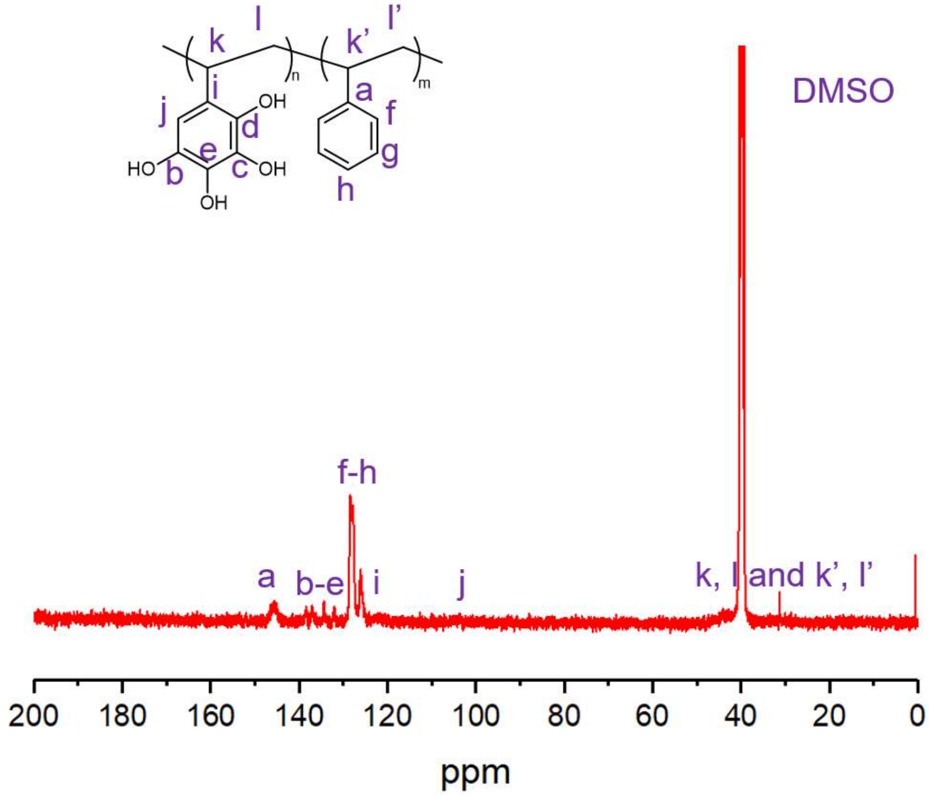

Supplementary Fig. 4 | ^{1}H NMR spectrum of P(4HS$_{9\%}$-*co*-S$_{11\%}$) (400 MHz, DMSO-d$_6$) and ^{13}C NMR spectrum of P(4HS$_{9\%}$-*co*-S$_{11\%}$) (400 MHz, DMSO-d$_6$).

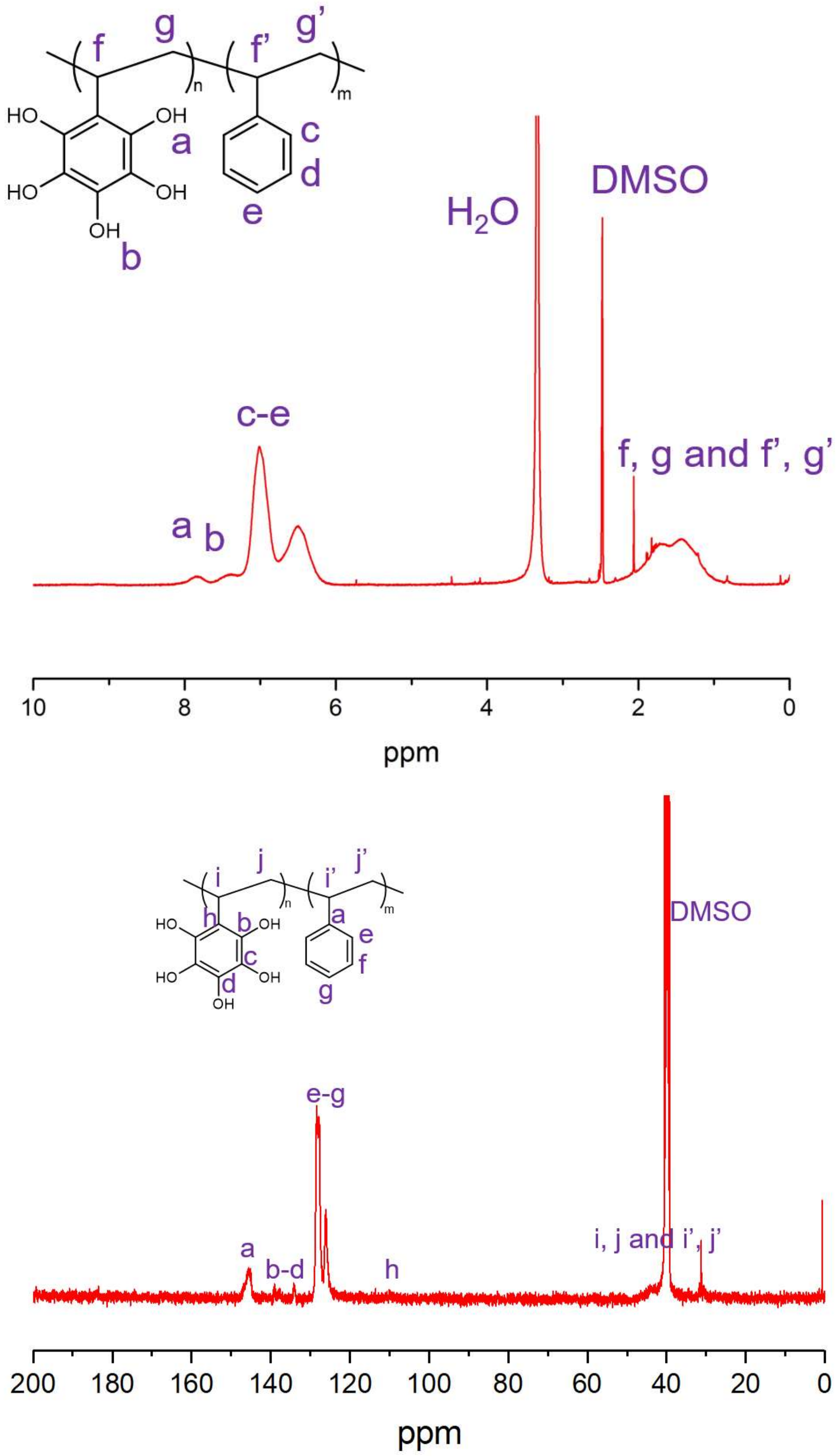

Supplementary Fig. 5 | ^{1}H NMR spectrum of P(5HS$_{11\%}$-*co*-S$_{89\%}$) (400 MHz, DMSO-d$_6$) and ^{13}C NMR spectrum of P(5HS$_{11\%}$-*co*-S$_{89\%}$) (400 MHz, DMSO-d$_6$).

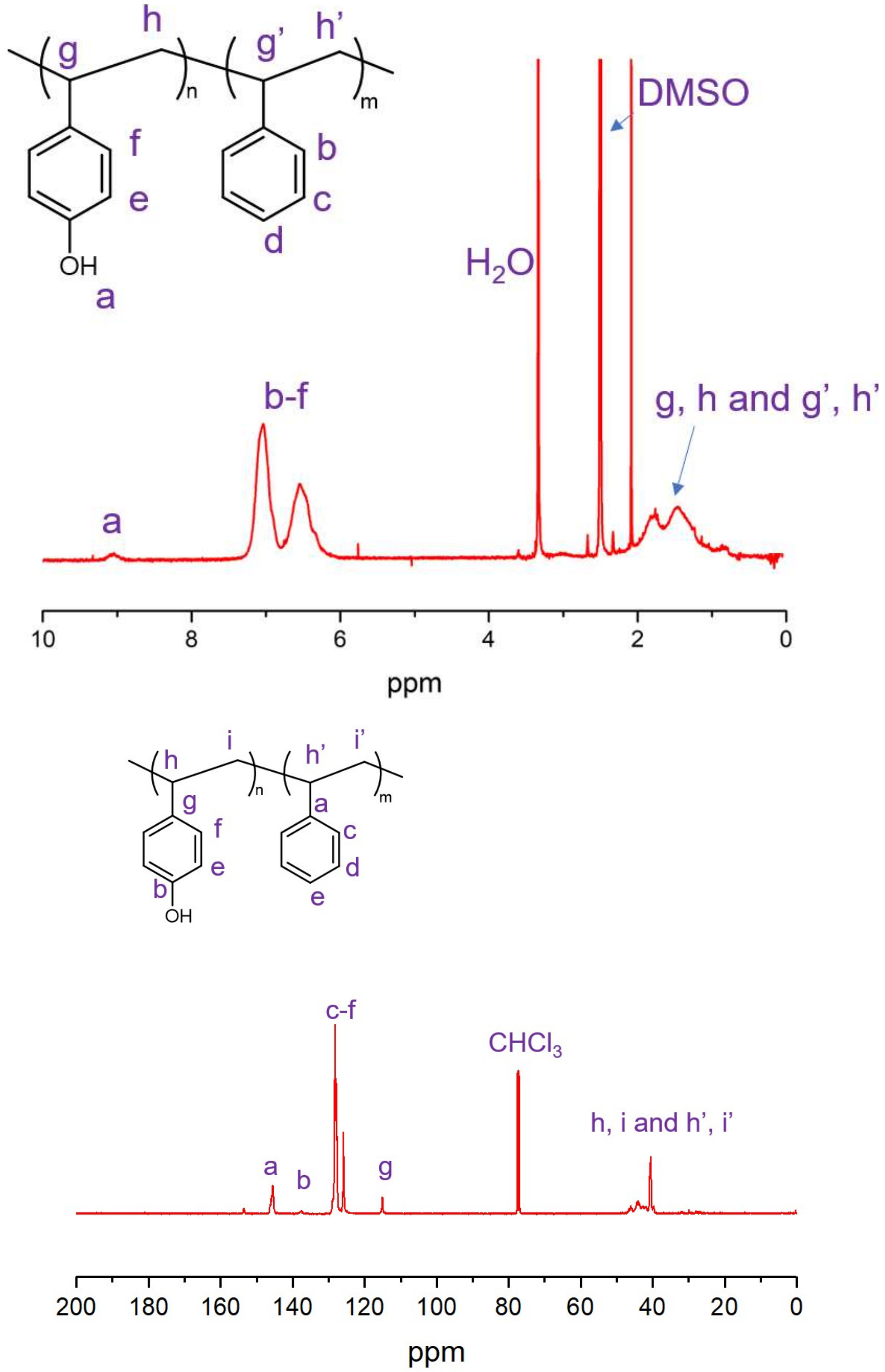

Supplementary Fig. 6 | ^{1}H NMR spectrum of P(1HS$_{10\%}$-*co*-S$_{90\%}$) (400 MHz, DMSO-d$_6$) and ^{13}C NMR spectrum of P(1HS$_{10\%}$-*co*-S$_{90\%}$) (400 MHz, $CDCl_3$).

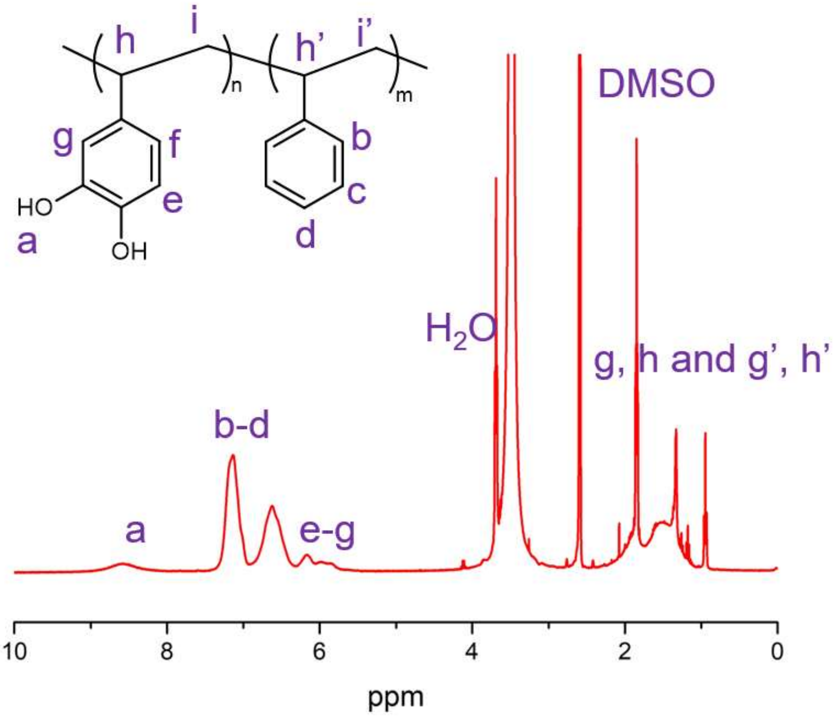

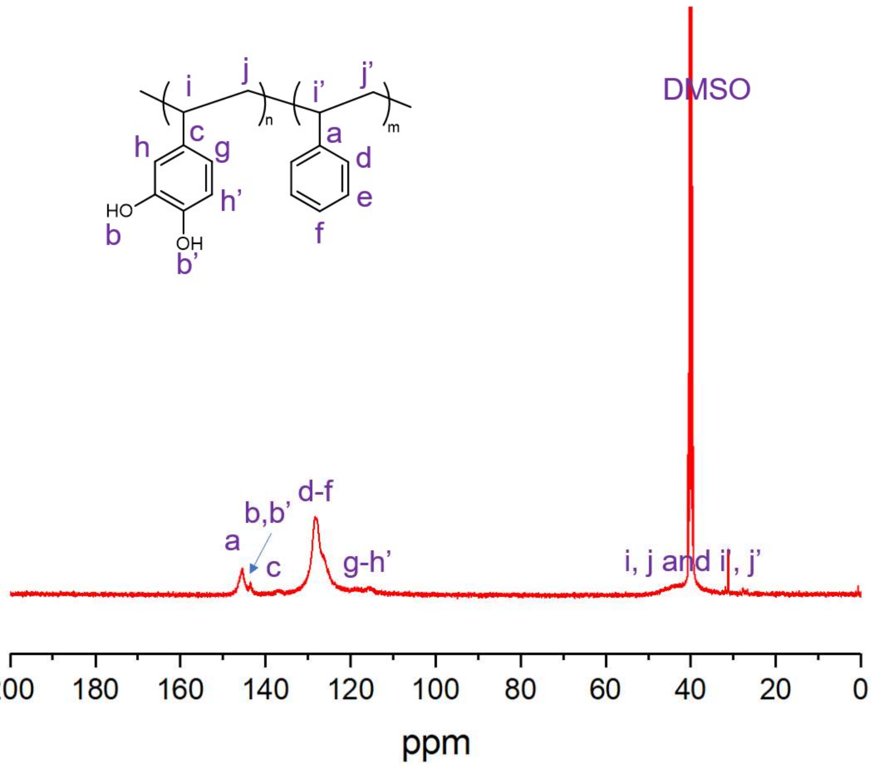

Supplementary Fig. 7 | ^{1}H NMR spectrum of P(2HS$_{11\%}$-*co*-S$_{89\%}$) (400 MHz, DMSO-d$_6$) and ^{13}C NMR spectrum of P(2HS$_{11\%}$-*co*-S$_{89\%}$) (400 MHz, DMSO-d$_6$).

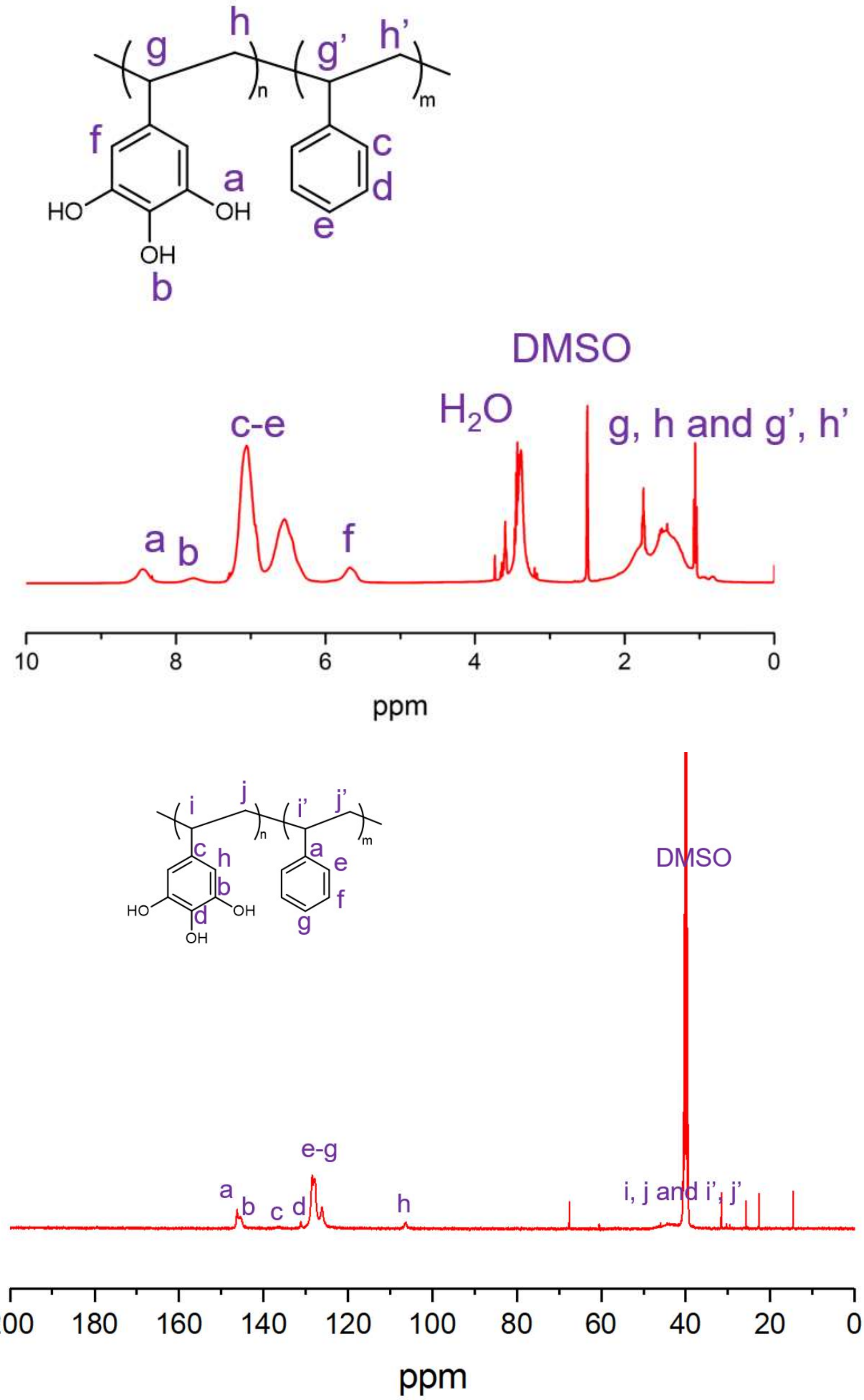

Supplementary Fig. 8 | ^{1}H NMR spectrum of P(3HS$_{11\%}$-*co*-S$_{89\%}$) (400 MHz, DMSO-d$_6$) and ^{13}C NMR spectrum of P(3HS$_{11\%}$-*co*-S$_{89\%}$) (400 MHz, DMSO-d$_6$).

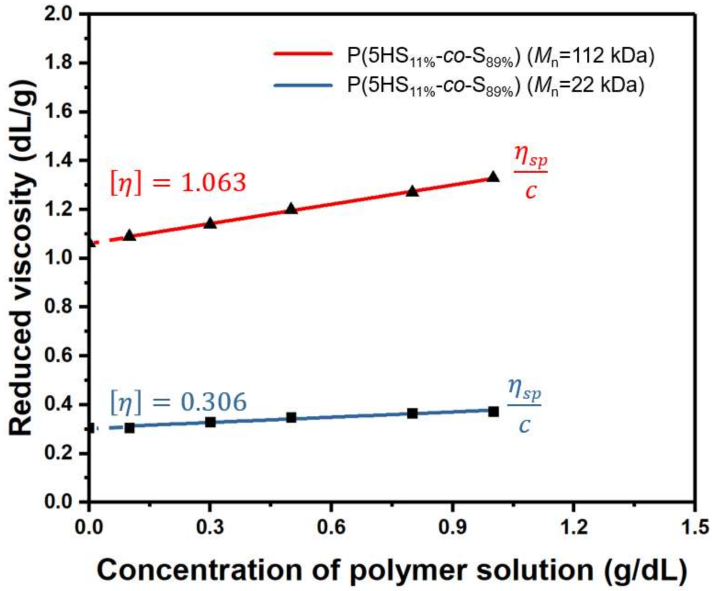

Critical concentration $c * \cong \frac{1}{[\eta]}$

$c*$ of P(5HS$_{11\%}$-*co*-S$_{89\%}$) (M_n=22 kDa) is estimated as 32.7 mg/mL.

$c*$ of P(5HS$_{11\%}$-*co*-S$_{89\%}$) (M_n=112 kDa) is estimated as 9.4 mg/mL.

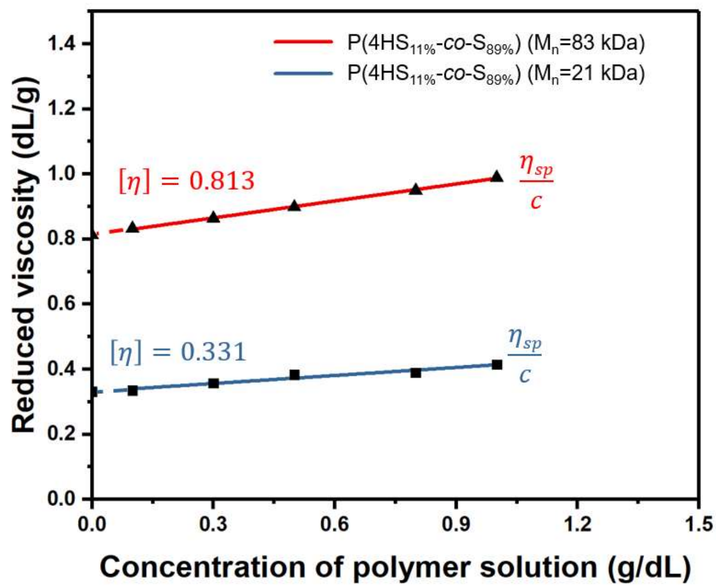

Critical concentration $c * \cong \frac{1}{[\eta]}$

$c*$ of P(4HS$_{11\%}$-*co*-S$_{89\%}$) (M_n=21 kDa) is estimated as 30.2 mg/mL.

$c*$ of P(4HS$_{11\%}$-*co*-S$_{89\%}$) (M_n=83 kDa) is estimated as 12.3 mg/mL.

Supplementary Fig. 9 | Estimation of intrinsic viscosity and critical concentration of 4HS and 5HS polymers with low and high M_n.

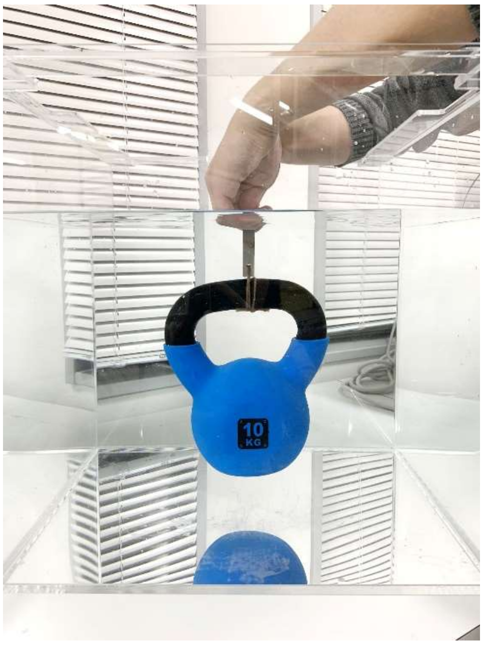

Supplementary Fig. 10 | Steel adhered with P(5HS$_{11\%}$-*co*-S$_{89\%}$) can lift 10-kg.

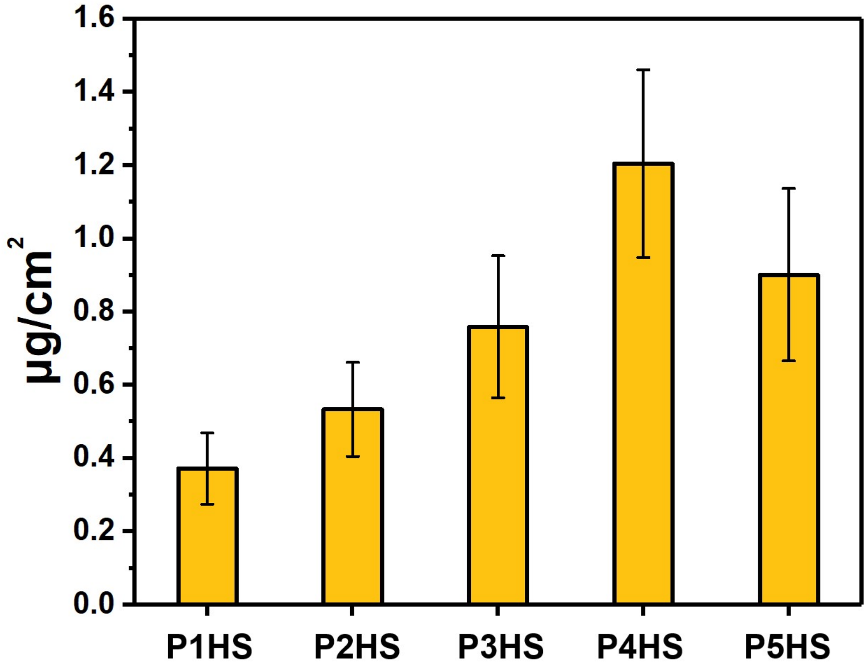

Supplementary Fig. 11 | Adsorbed quantities of phenolic homopolymers on Au QCM chips (0.05 wt% and immersed for 1 min). The results represent the mean ± s.d. (n = 3).

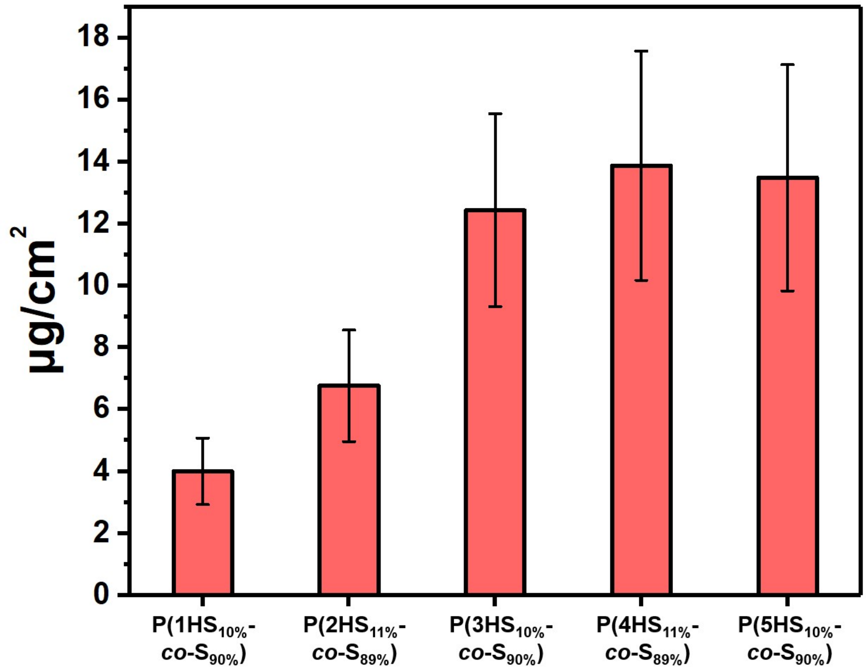

Supplementary Fig. 12 | Adsorbed quantities of phenolic copolymers on Au QCM chips. Polymers with 20 kDa of M_n were used. The results represent the mean ± s.d. (n = 3).

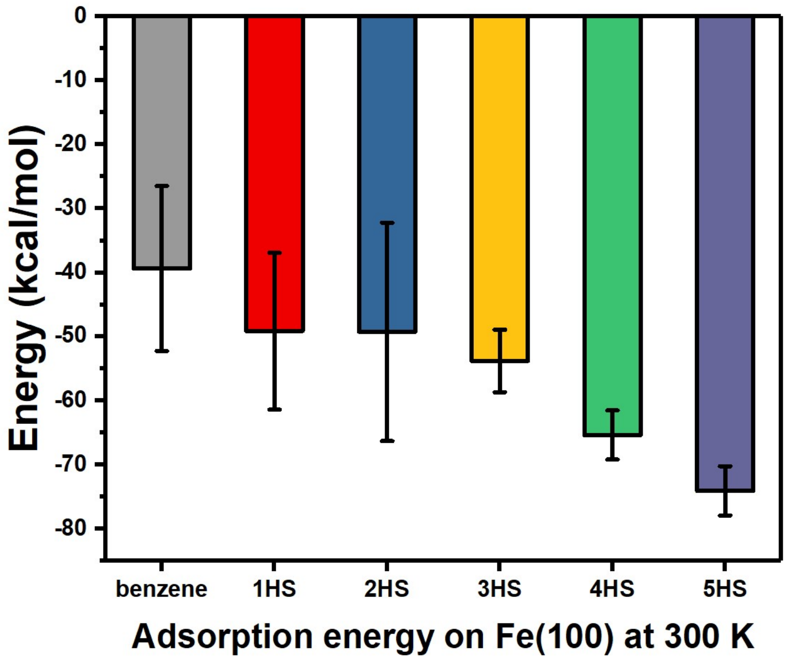

Supplementary Fig. 13 | MD-simulation of the adsorption energies of benzene, 1HS, 2HS, 3HS, 4HS, and 5HS to Fe(100) surfaces at 300 K. The results represent the mean ± s.d. (*n* = 5).

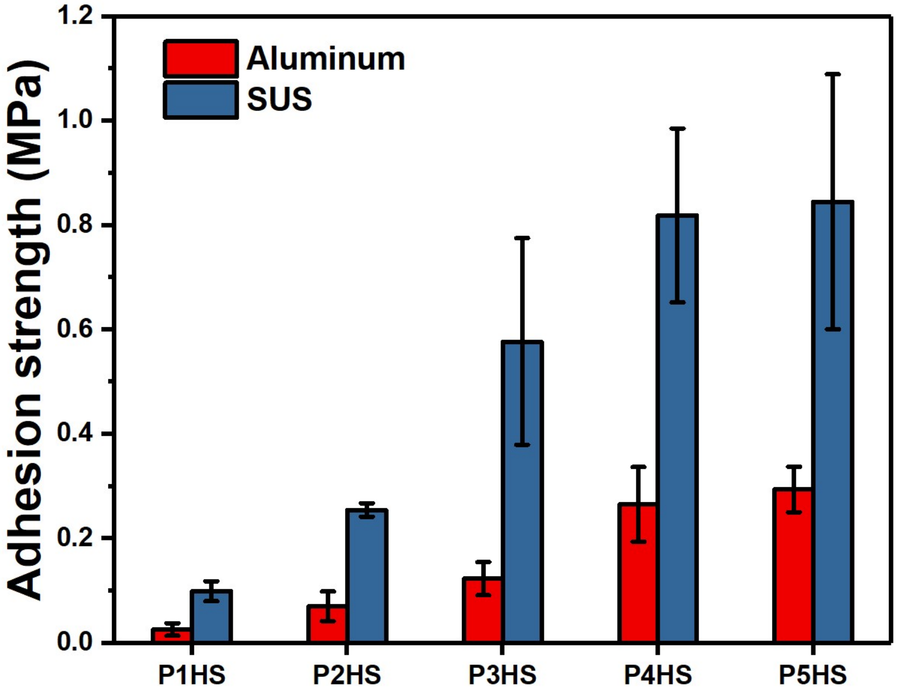

Supplementary Fig. 14 | Adhesion strength of P1HS, P2HS, P3HS, P4HS, and P5HS (homopolymers) after setting for 1 day in air. The results represent the mean ± s.d. (*n* = 5).

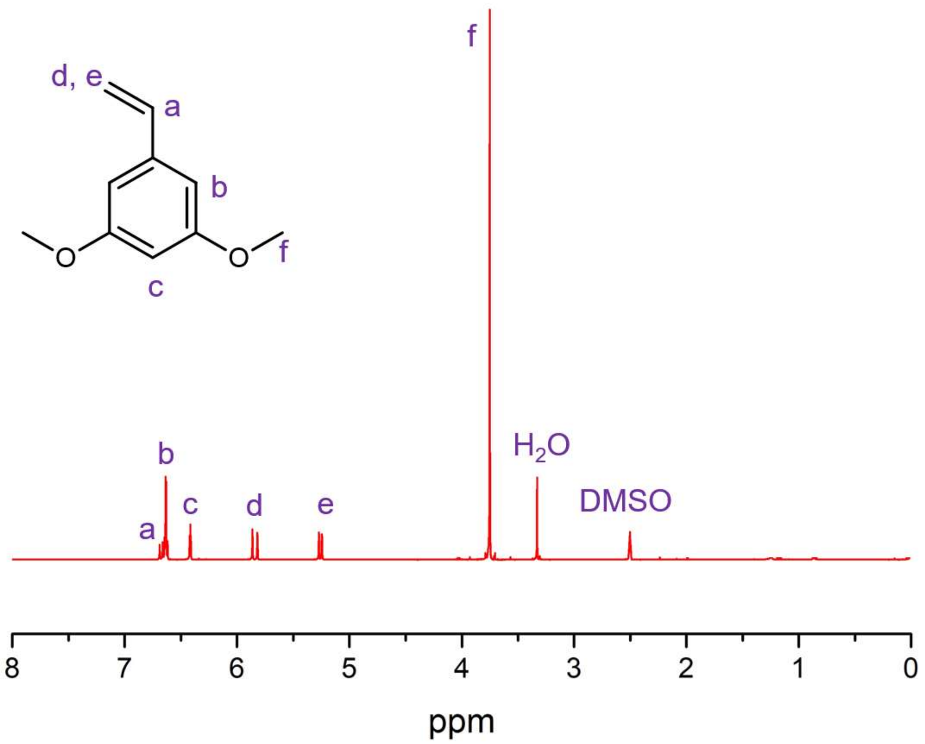

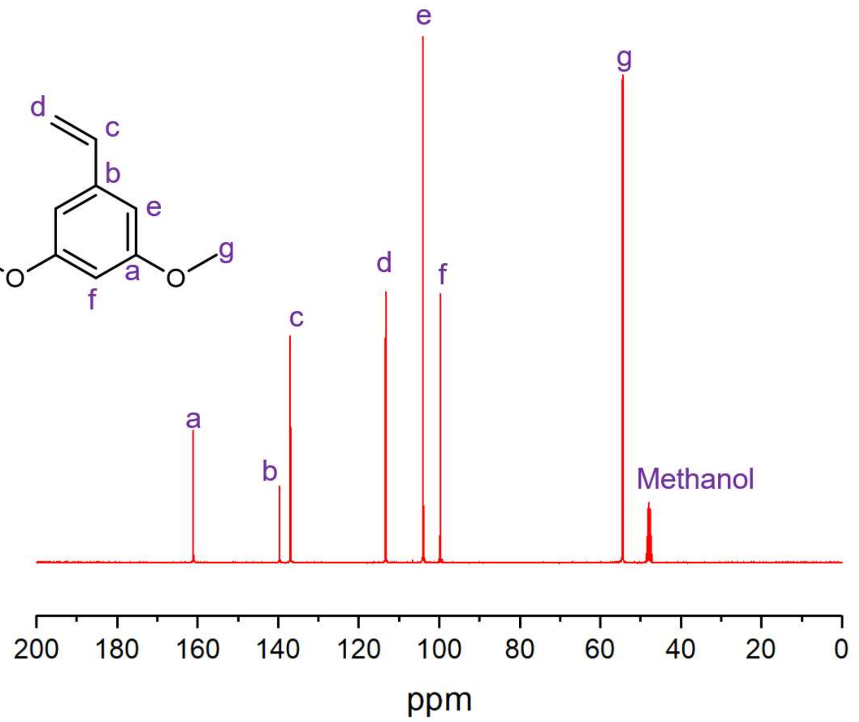

Supplementary Fig. 15 | ^{1}H NMR spectrum of 3,5-dimethoxystyrene (isomer of 2HS) (400 MHz, DMSO-d$_6$) and ^{13}C NMR spectrum of 3,5-dimethoxystyrene (400 MHz, Methanol-d$_4$).

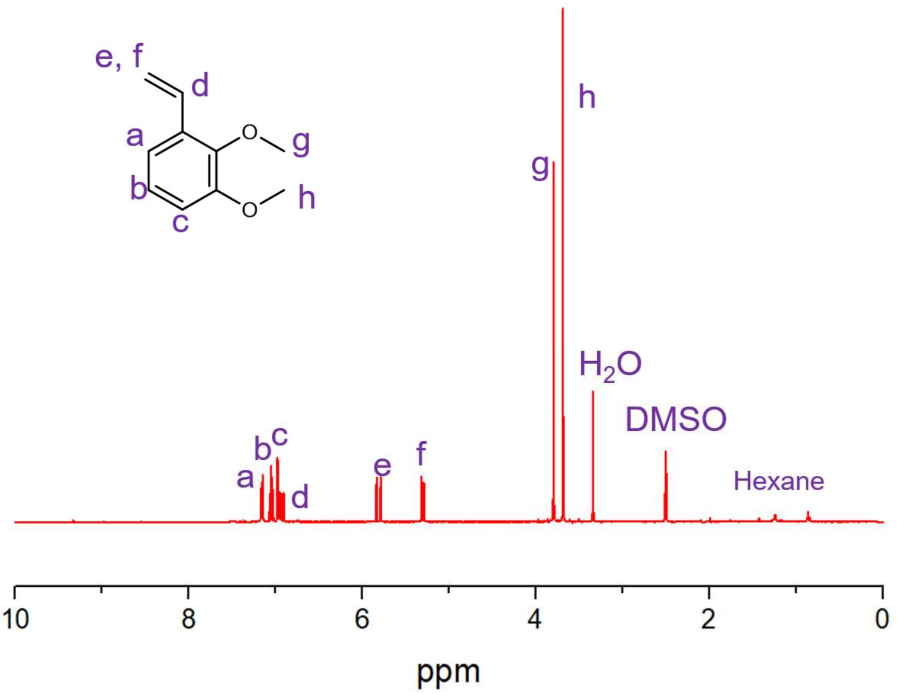

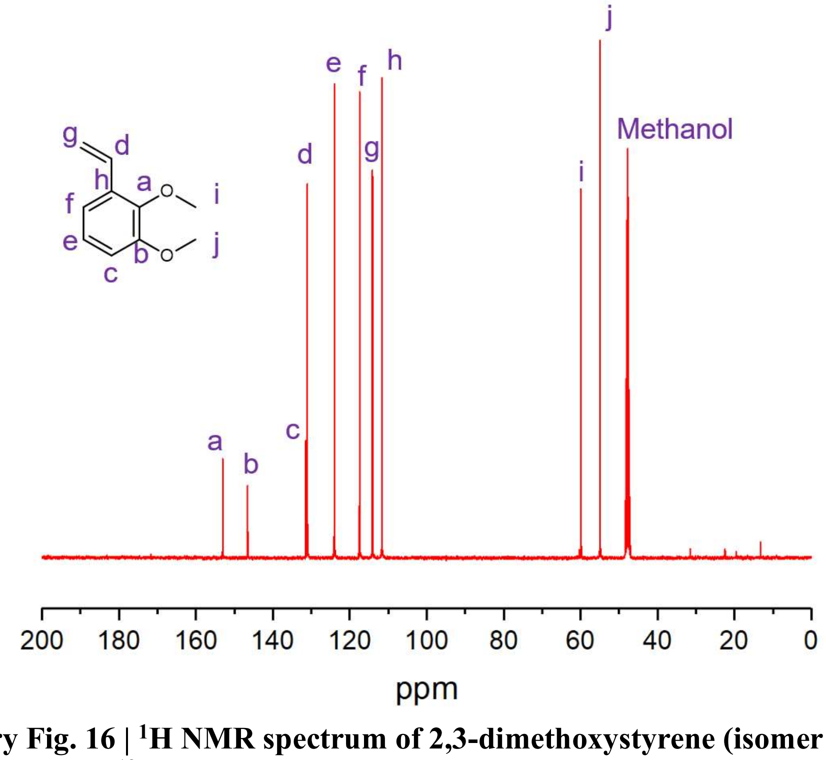

Supplementary Fig. 16 | ^{1}H NMR spectrum of 2,3-dimethoxystyrene (isomer of 2HS) (400 MHz, DMSO-d_6) and ^{13}C NMR spectrum of 2,3-dimethoxystyrene (400 MHz, Methanol Methanol-d_4).

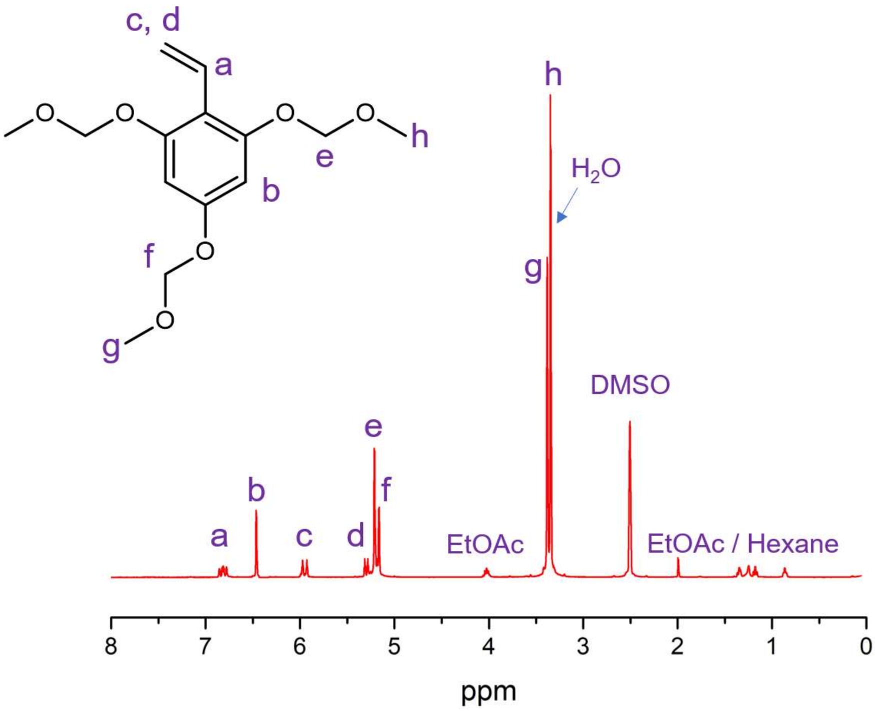

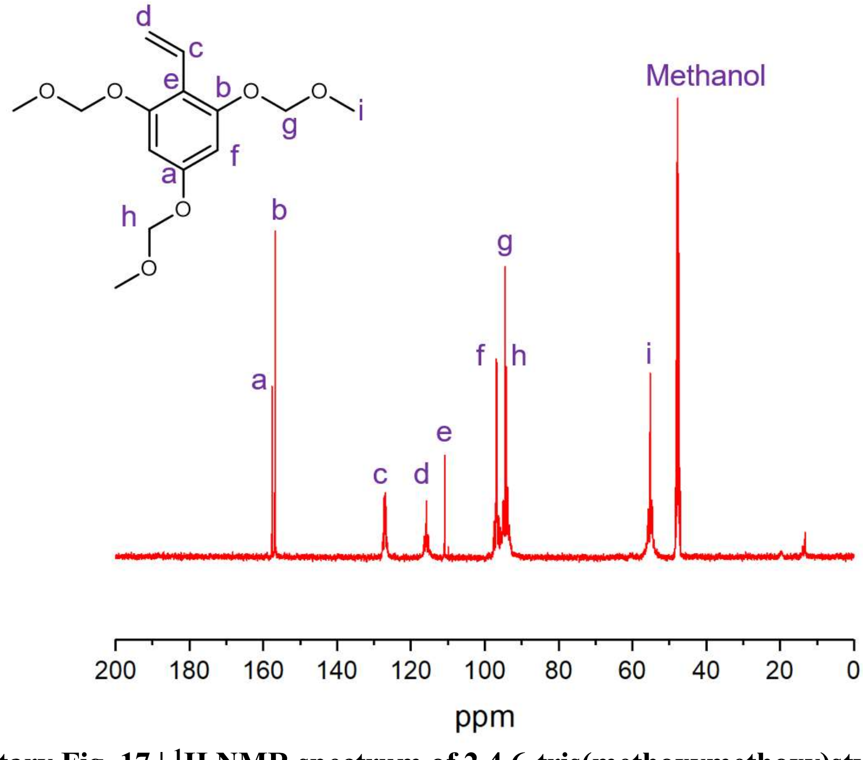

Supplementary Fig. 17 | ^{1}H NMR spectrum of 2,4,6-tris(methoxymethoxy)styrene (isomer of 3HS) (400 MHz, DMSO-d_6) and ^{13}C NMR spectrum of 2,4,6-tris(methoxymethoxy)styrene (400 MHz, Methanol-d_4).

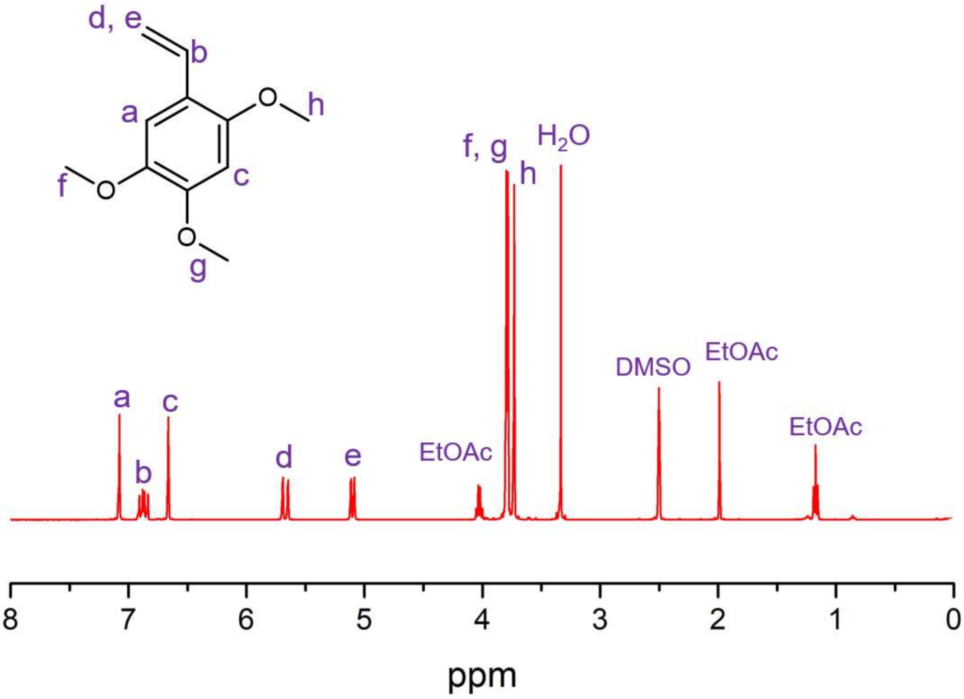

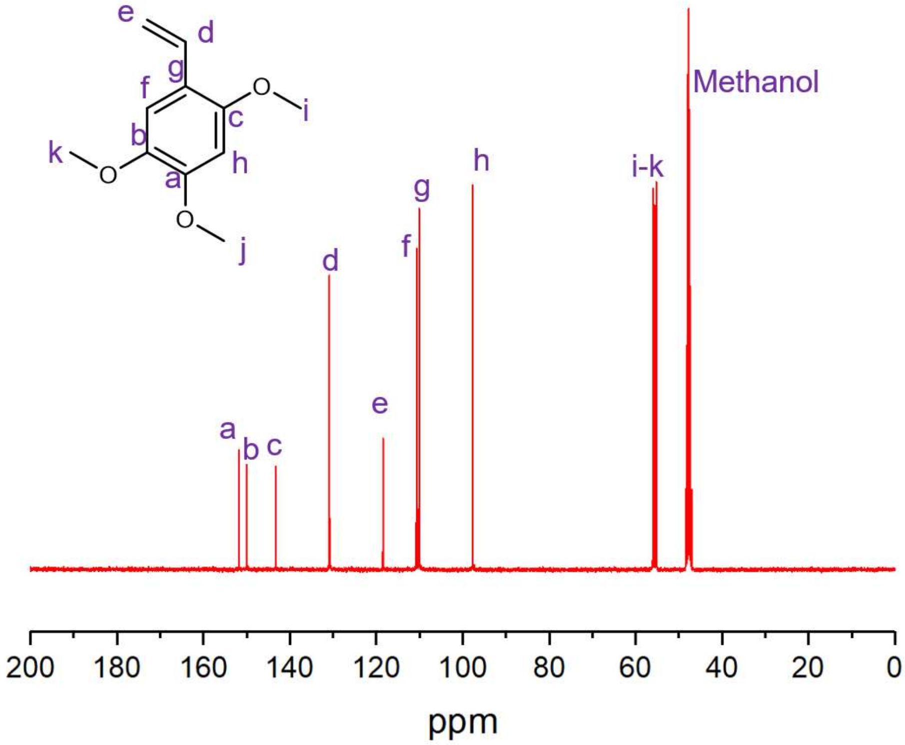

Supplementary Fig. 18 | ^{1}H NMR spectrum of 2,4,5-trimethoxystyrene (isomer of 3HS) (400 MHz, DMSO-d_6) and ^{13}C NMR spectrum of 2,4,5-trimethoxystyrene (400 MHz, Methanol-d_4).

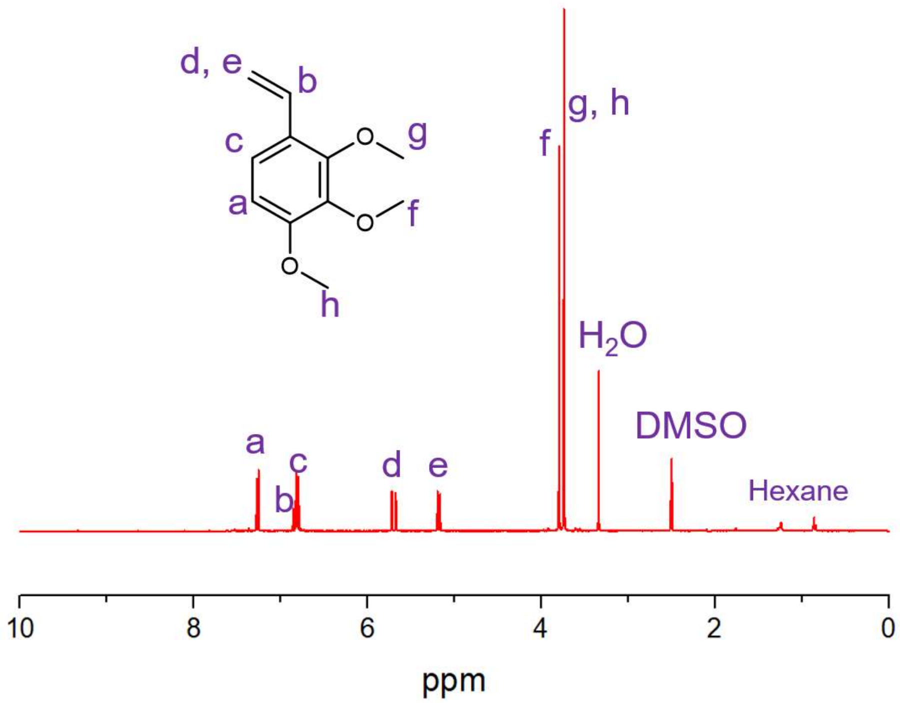

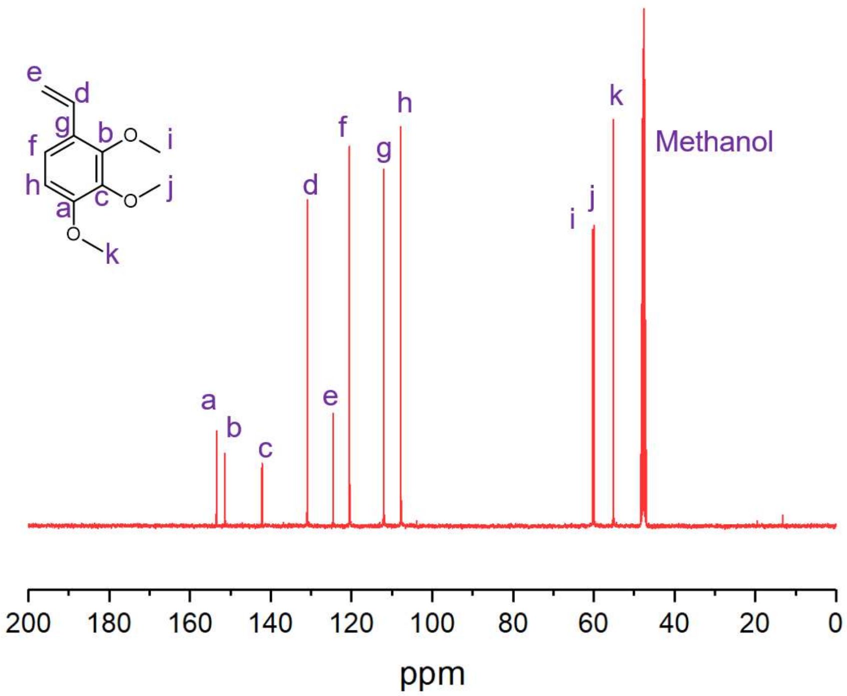

Supplementary Fig. 19 | ^{1}H NMR spectrum of 2,3,4-trimethoxystyrene (isomer of 3HS) (400 MHz, DMSO-d$_6$) and ^{13}C NMR spectrum of 2,3,4-trimethoxystyrene (400 MHz, Methanol-d$_4$).

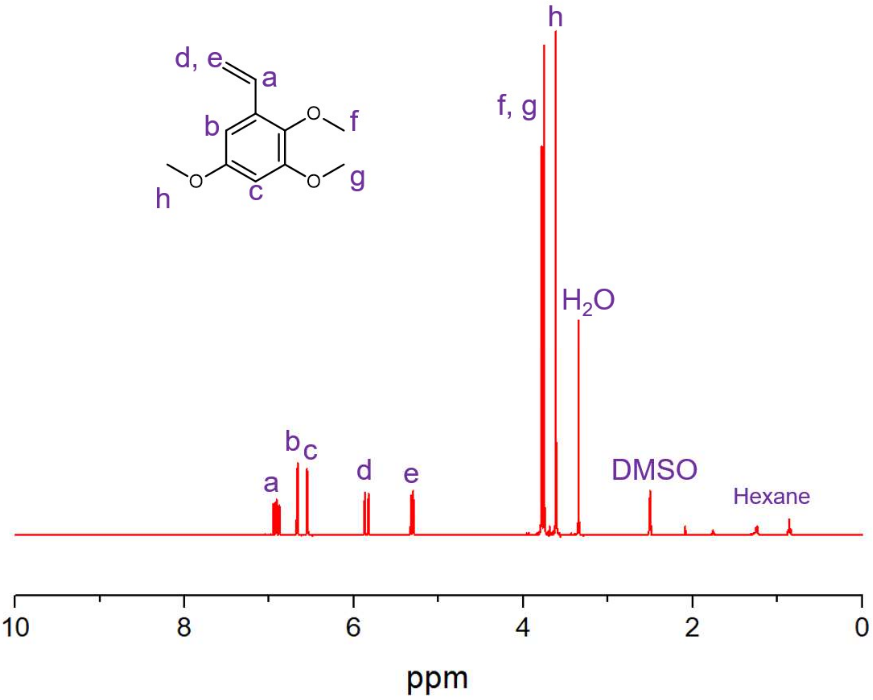

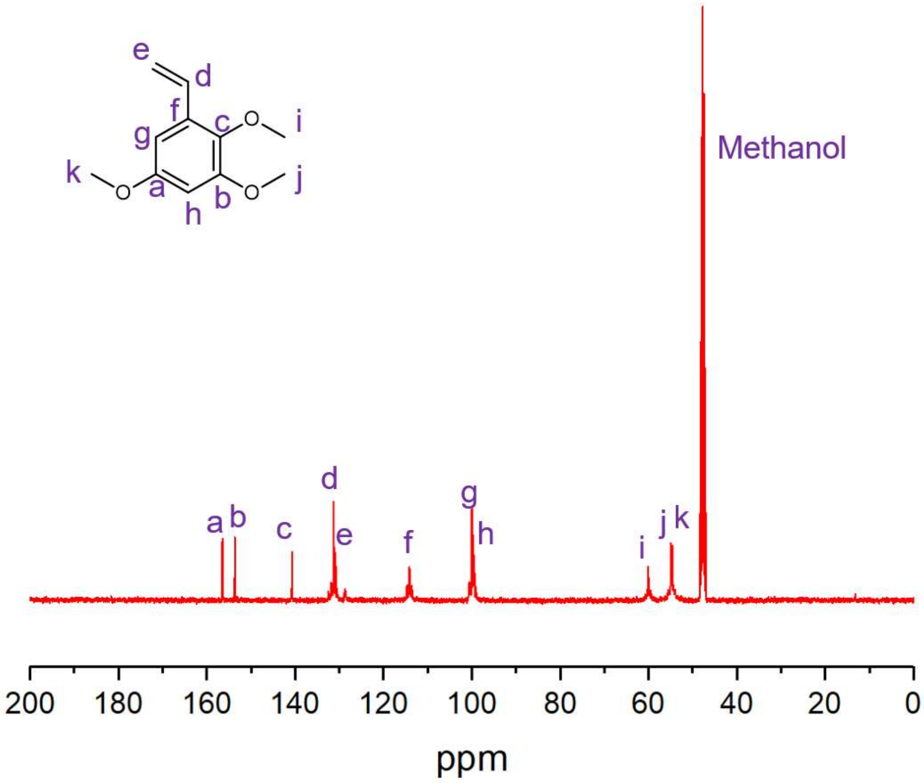

Supplementary Fig. 20 | ^{1}H NMR spectrum of 2,3,5-trimethoxystyrene (isomer of 3HS) (400 MHz, DMSO-d$_6$) and ^{13}C NMR spectrum of 2,3,5-trimethoxystyrene (400 MHz, Methanol Methanol-d$_4$).

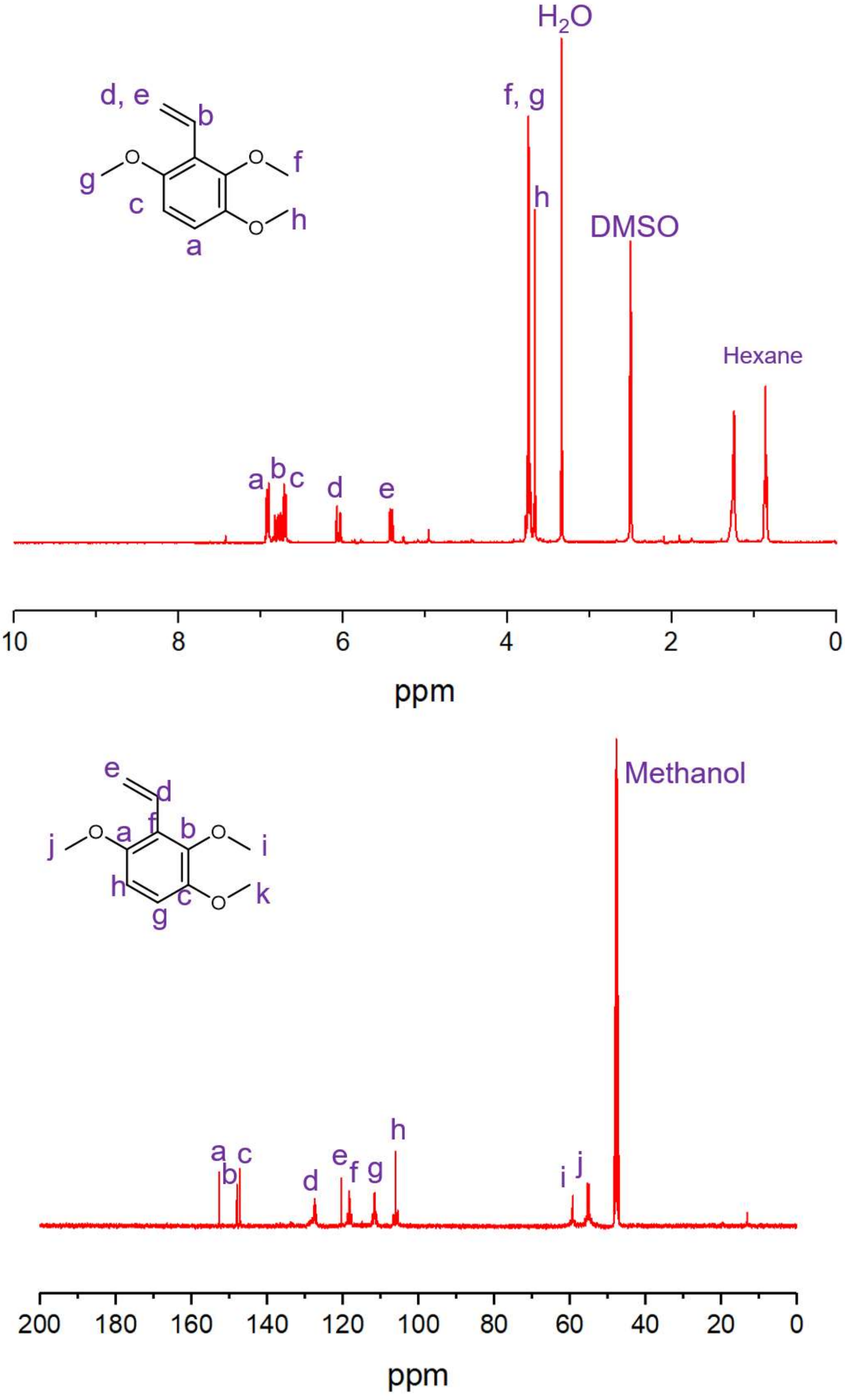

Supplementary Fig. 21 | ^{1}H NMR spectrum of 2,3,6-trimethoxystyrene (isomer of 3HS) (400 MHz, DMSO-d$_6$) and ^{13}C NMR spectrum of 2,3,6-trimethoxystyrene (400 MHz, Methanol-d$_4$).

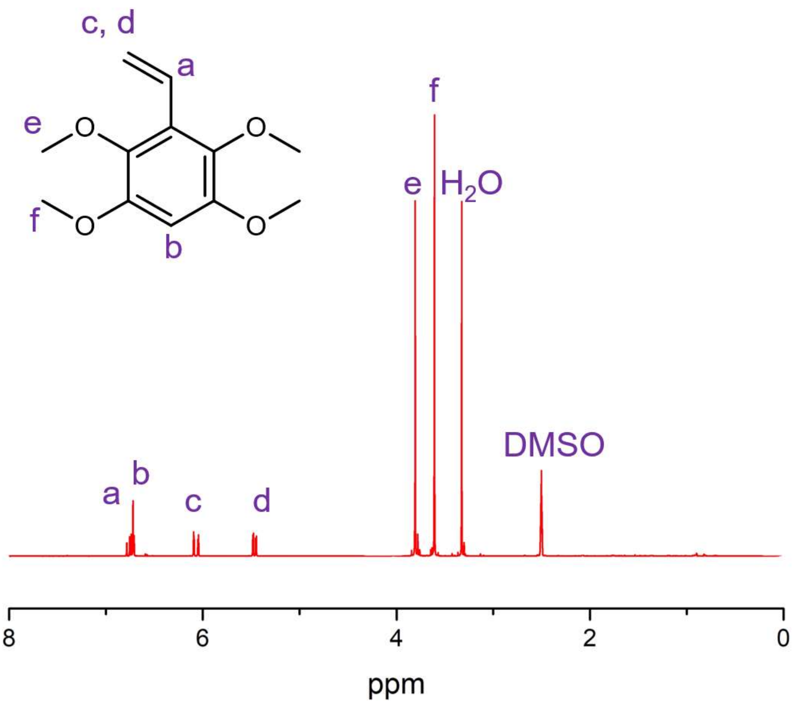

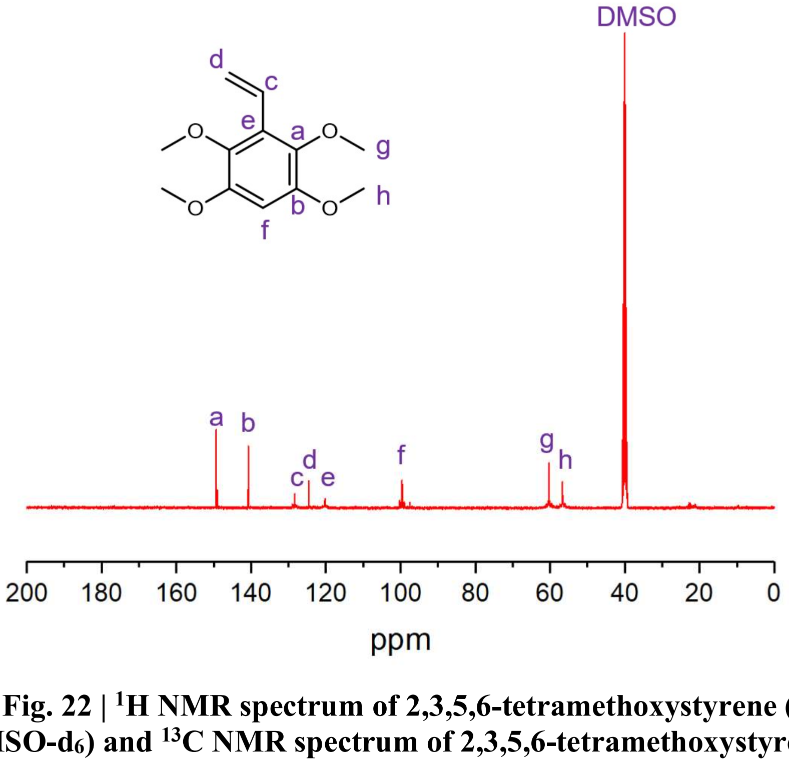

Supplementary Fig. 22 | ^{1}H NMR spectrum of 2,3,5,6-tetramethoxystyrene (isomer of 4HS) (400 MHz, DMSO-d_6) and ^{13}C NMR spectrum of 2,3,5,6-tetramethoxystyrene (400 MHz, DMSO-d_6).

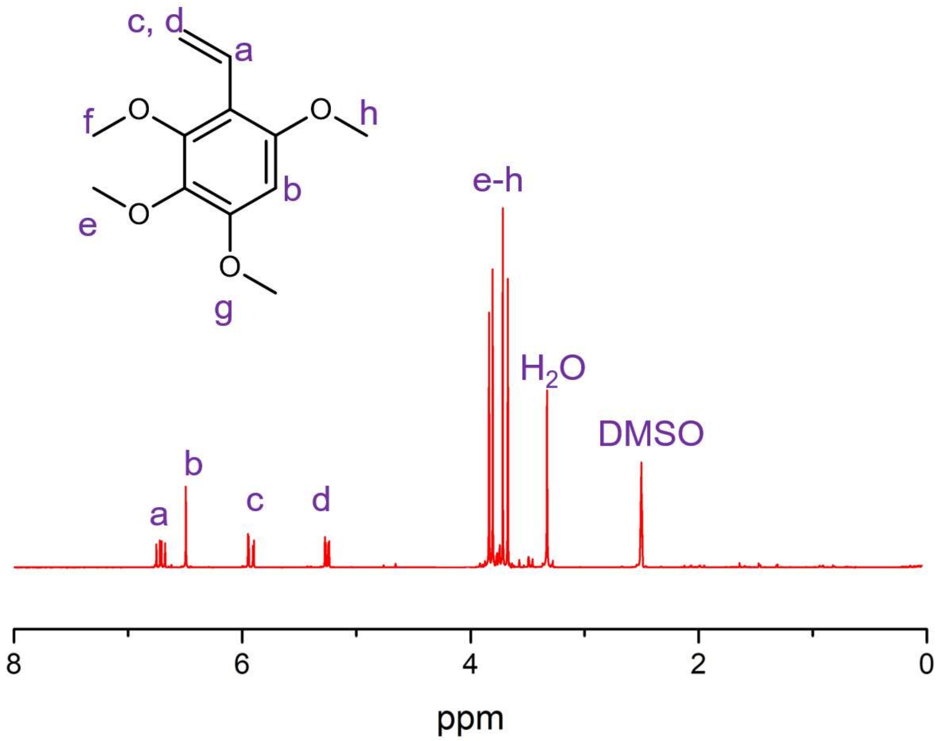

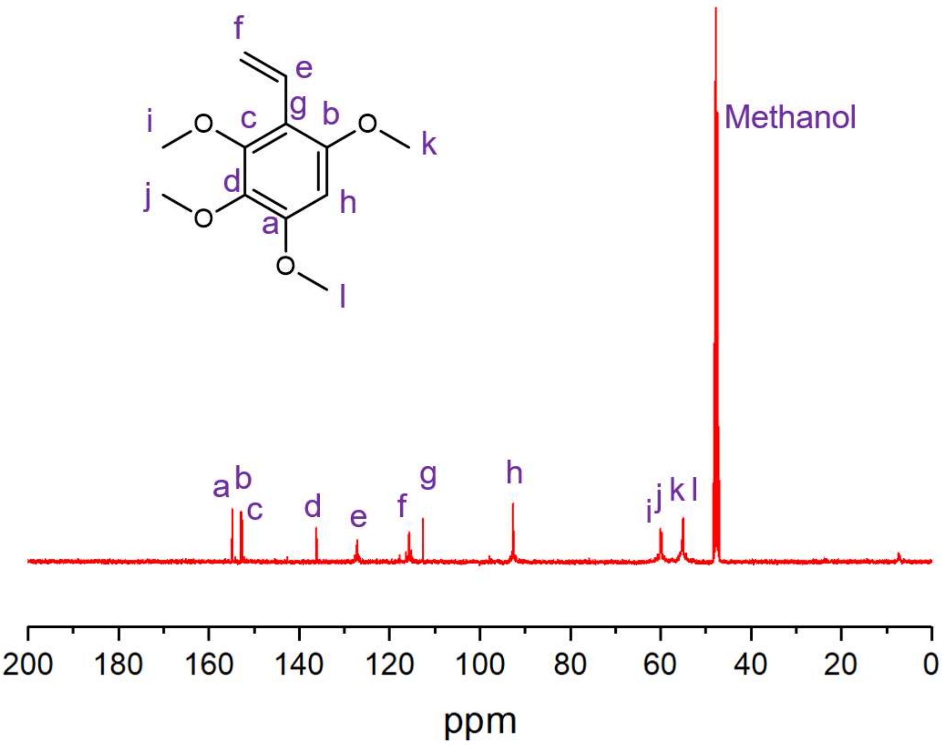

Supplementary Fig. 23 | ^{1}H NMR spectrum of 2,3,4,6-tetramethoxystyrene (isomer of 4HS) (400 MHz, DMSO-d$_6$) and ^{13}C NMR spectrum of 2,3,4,6-tetramethoxystyrene (400 MHz, Methanol-d$_4$).

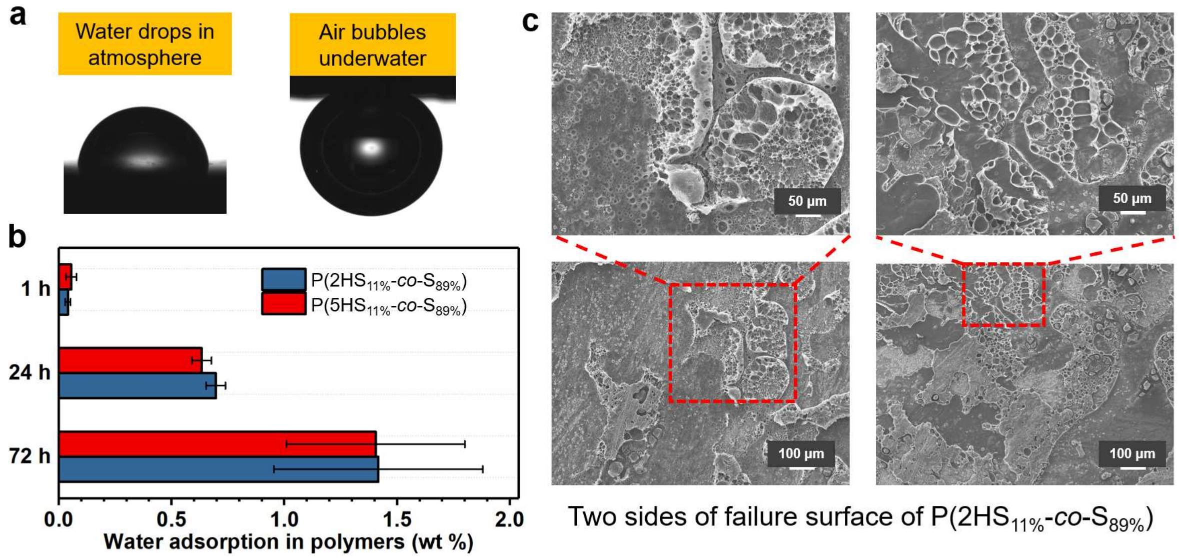

Supplementary Fig. 24 | Hydrophilicity (a), water adsorption ability (b), and SEM morphology of P(2HS$_{11\%}$-*co*-S$_{89\%}$) (c). The results in (b) represent the mean ± s.d. (*n* = 3).

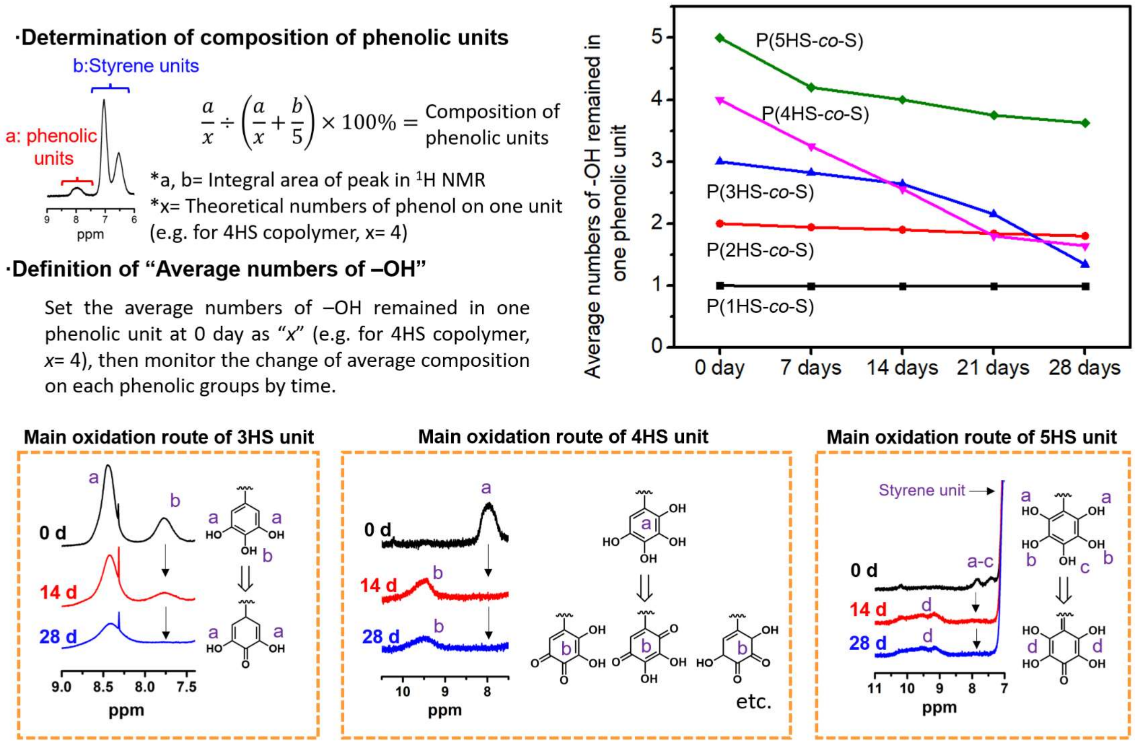

Supplementary Fig. 25 | The oxidation of phenolic polymers traced by ^{1}H NMR and possible oxidation routes of 3HS, 4HS and 5HS units.

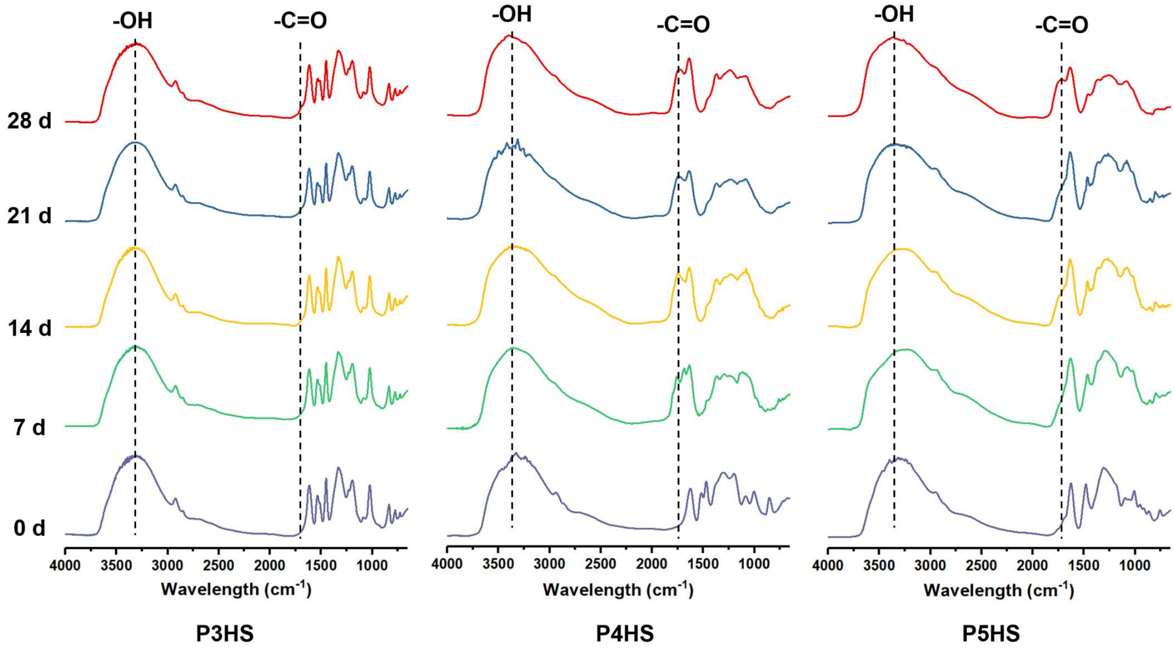

Supplementary Fig. 26 | FT-IR spectra tracking the oxidation of different phenolic polymers over time.

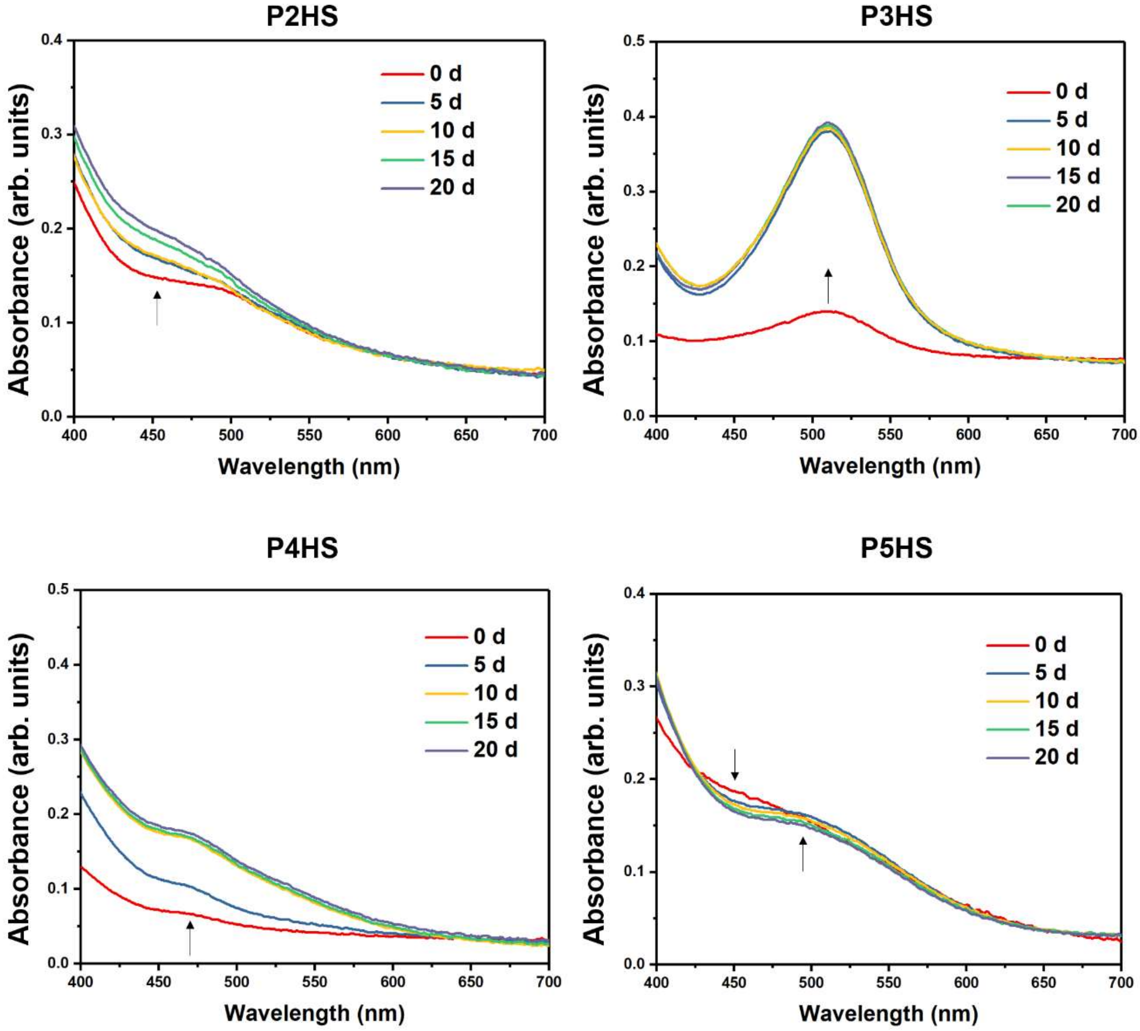

Supplementary Fig. 27 | The oxidation of phenolic polymers over time as tracked by UV-Vis.

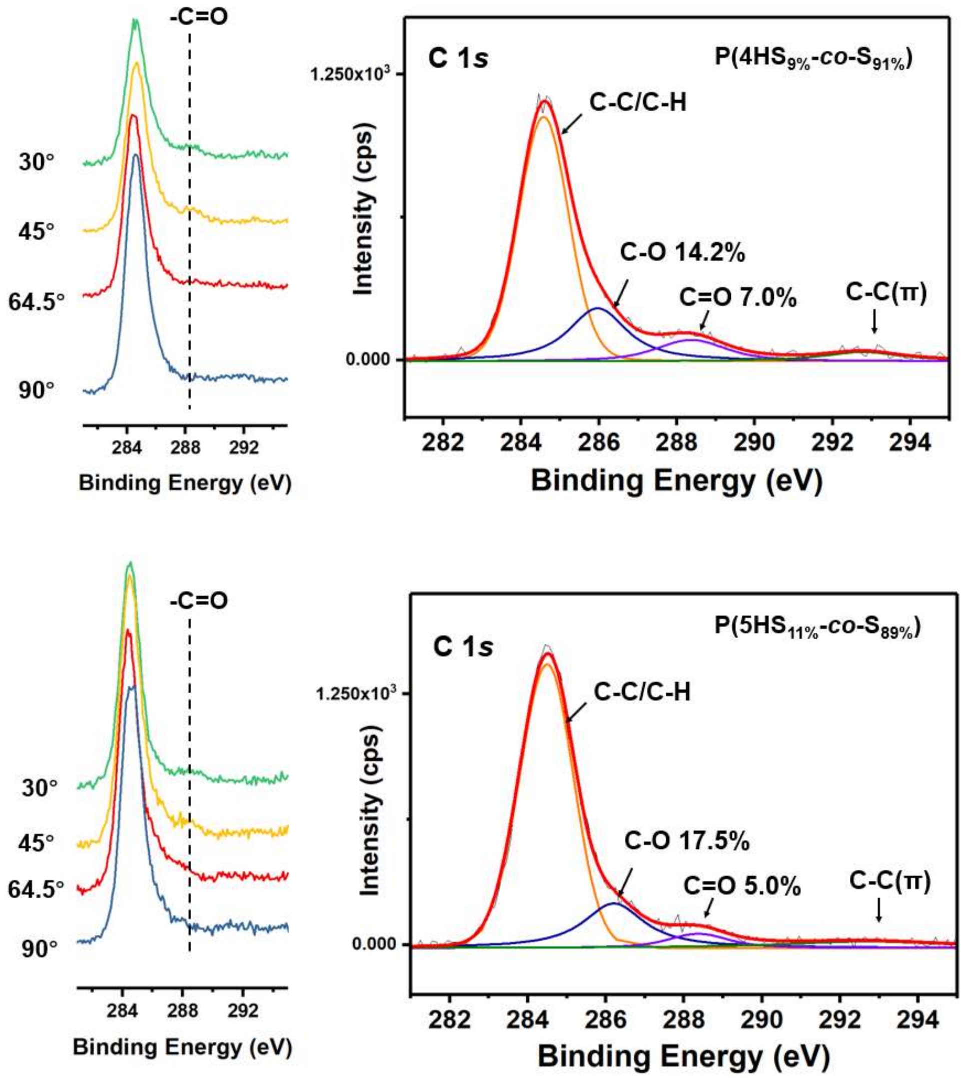

Supplementary Fig. 28 | Depth-dependent XPS of 4HS and 5HS polymers.

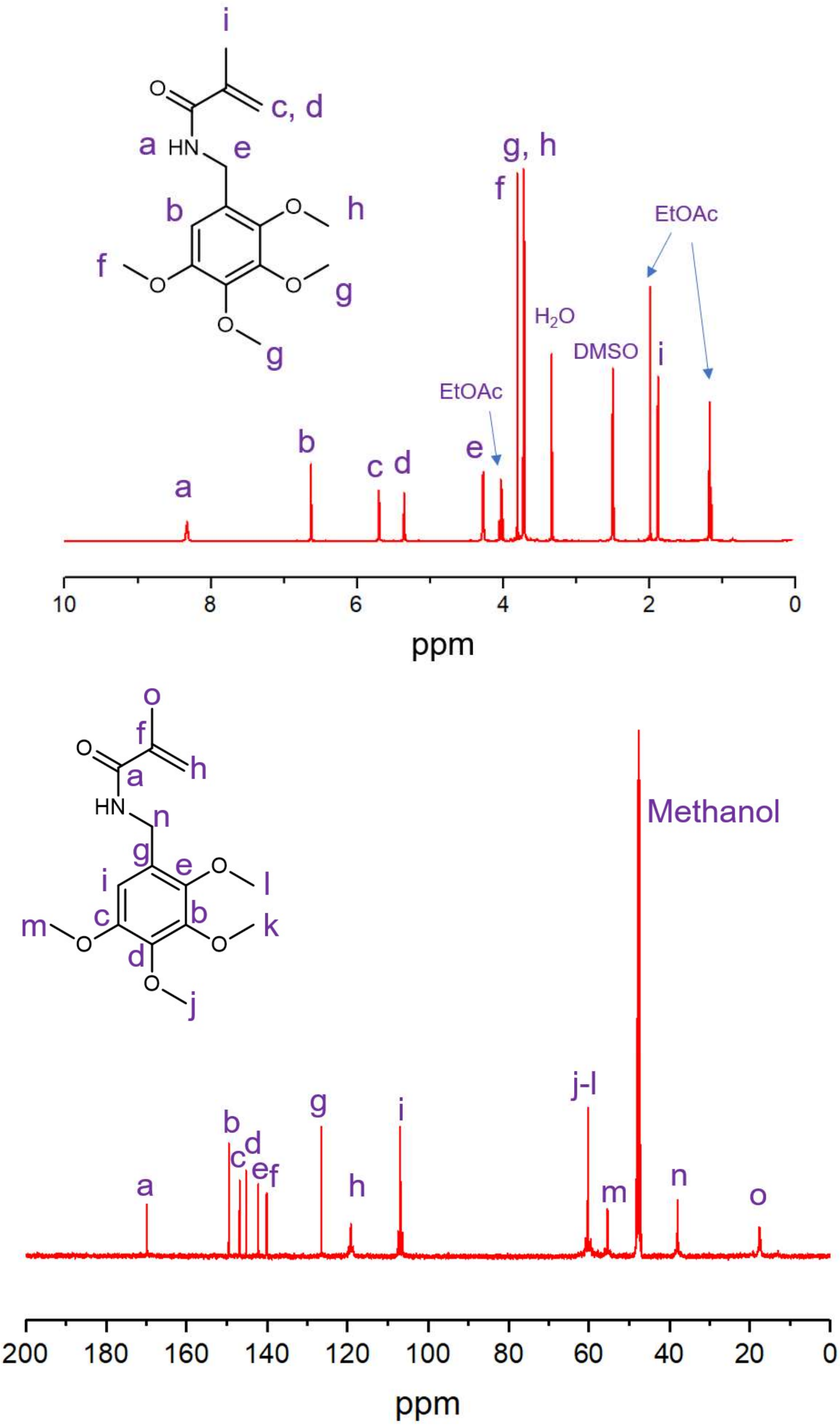

Supplementary Fig. 29 | ^{1}H NMR spectrum of TMA (400 MHz, DMSO-d$_6$) and ^{13}C NMR spectrum of TMA (400 MHz, Methanol-d$_4$).

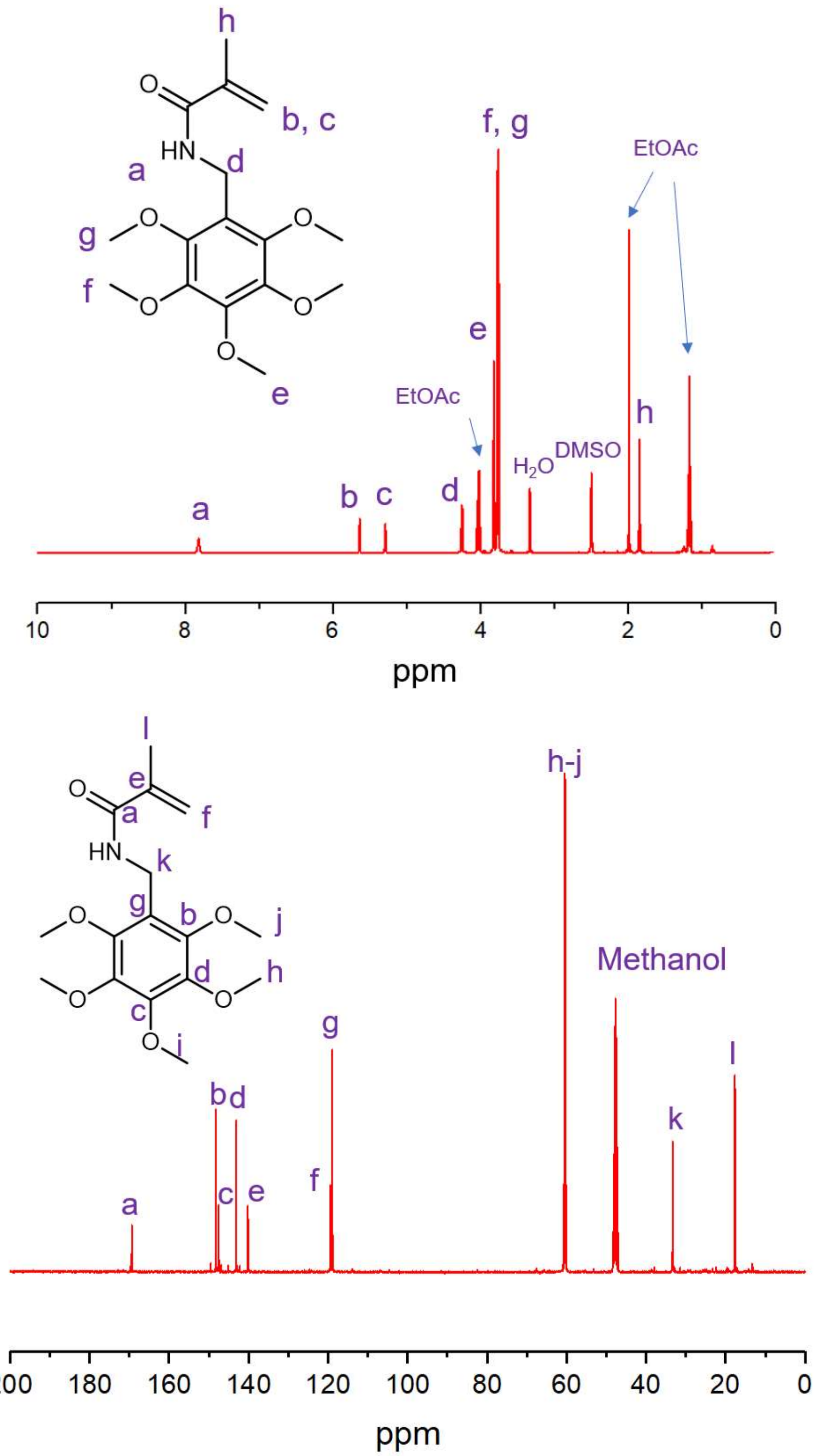

Supplementary Fig. 30 | ^{1}H NMR spectrum of PMA (400 MHz, DMSO-d_6) and ^{13}C NMR spectrum of PMA (400 MHz, Methanol-d_4).

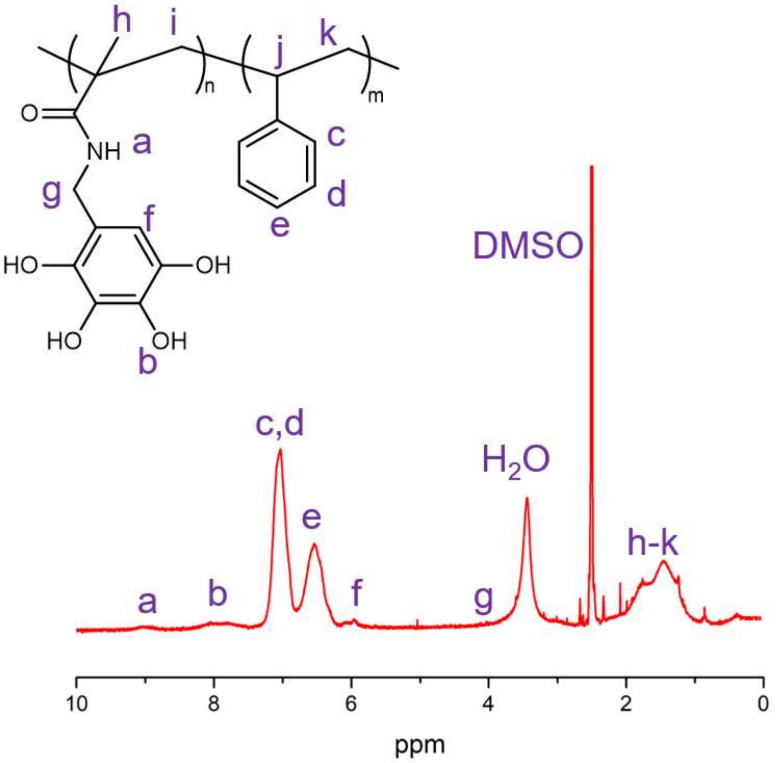

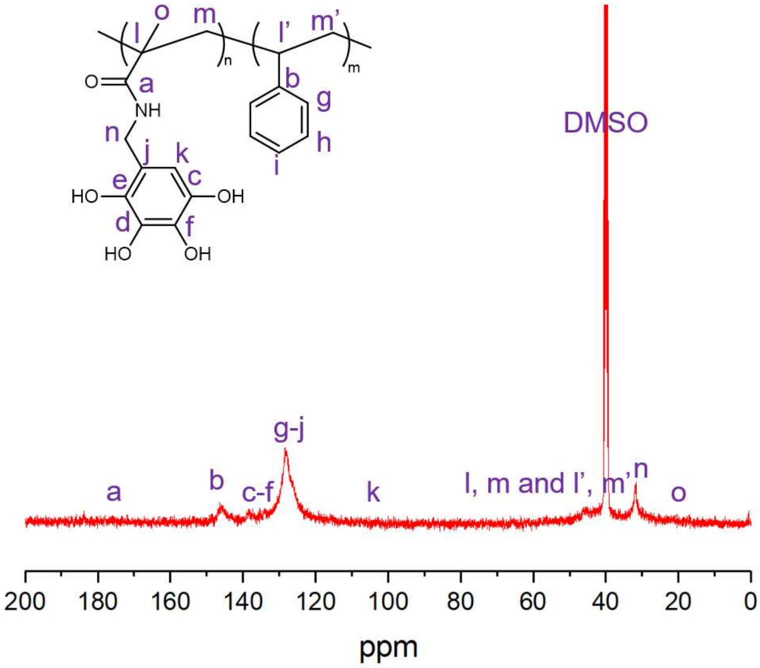

Supplementary Fig. 31 | ^{1}H NMR spectrum of P(4HMA$_{8\%}$-*co*-S$_{92\%}$) (400 MHz, DMSO-d$_6$) and ^{13}C NMR spectrum of P(4HMA$_{8\%}$-*co*-S$_{92\%}$) (400 MHz, DMSO-d$_6$).

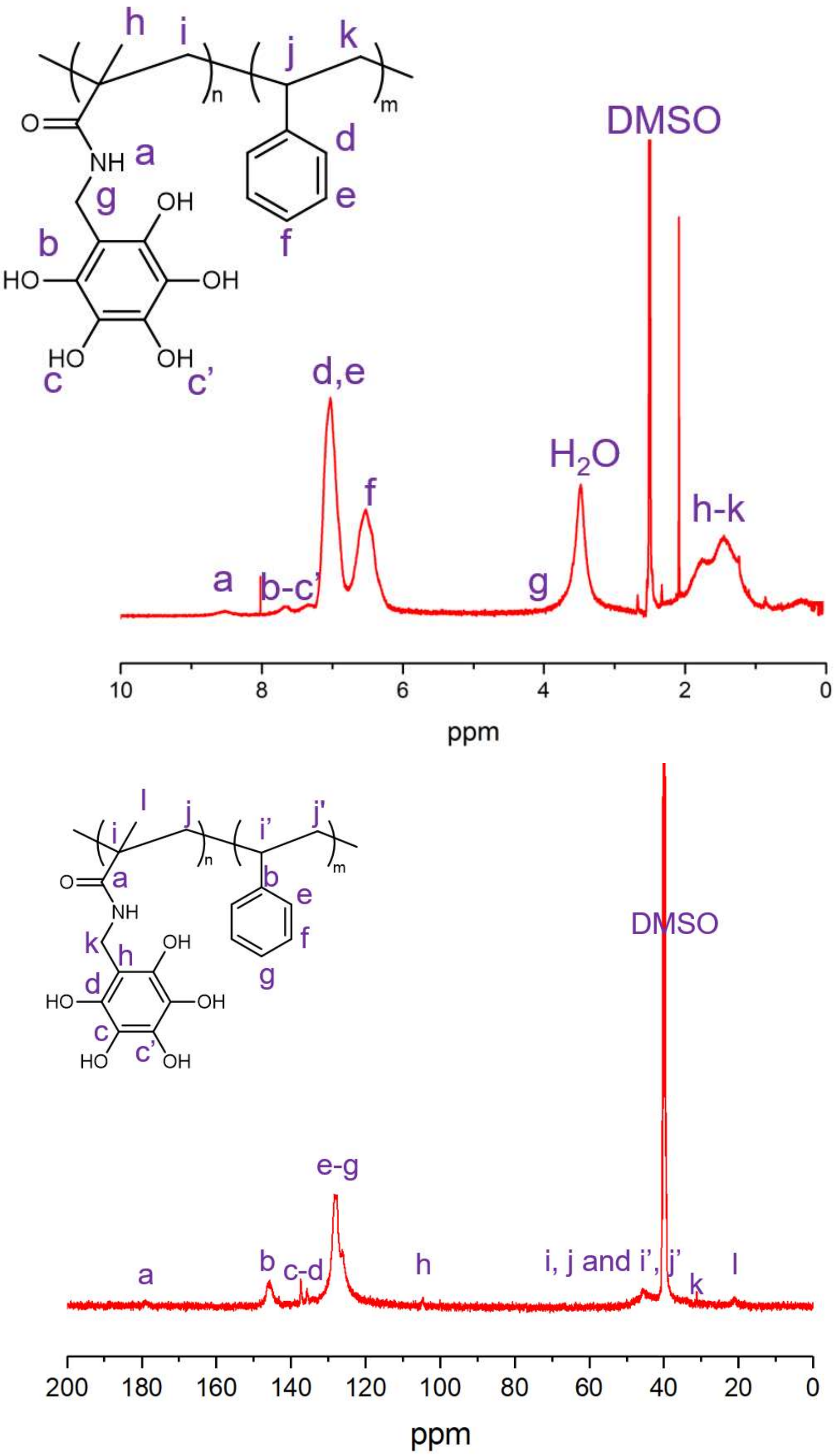

Supplementary Fig. 32 | ^{1}H NMR spectrum of P(5HMA$_{9\%}$-*co*-S$_{91\%}$) (400 MHz, DMSO-d$_6$) and ^{13}C NMR spectrum of P(5HMA$_{9\%}$-*co*-S$_{91\%}$) (400 MHz, DMSO-d$_6$).

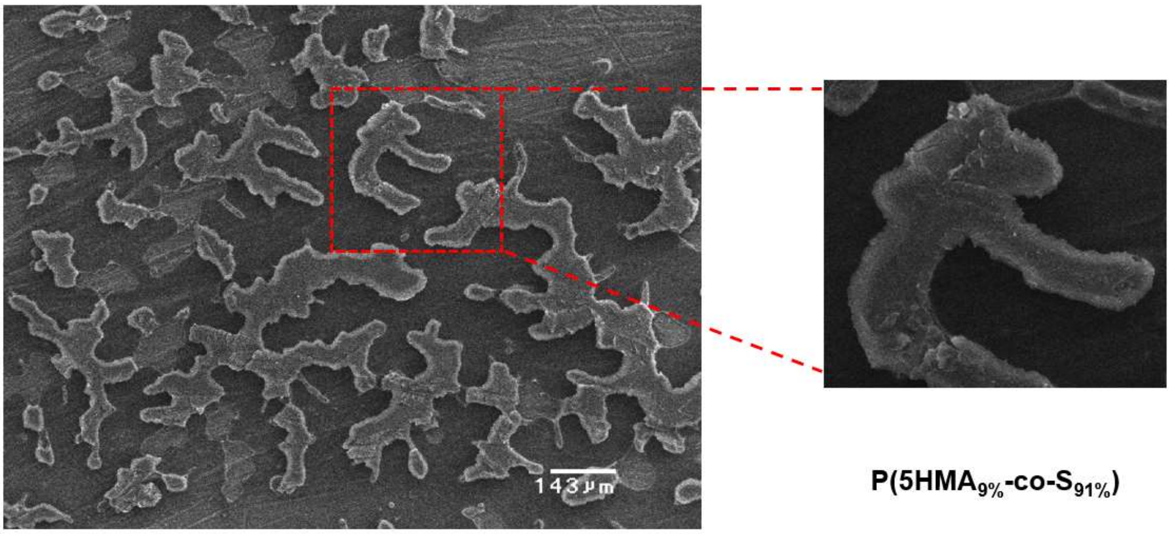

Supplementary Fig. 33 | Morphology of P(5HMA$_{9\%}$-*co*-S$_{91\%}$) by SEM.

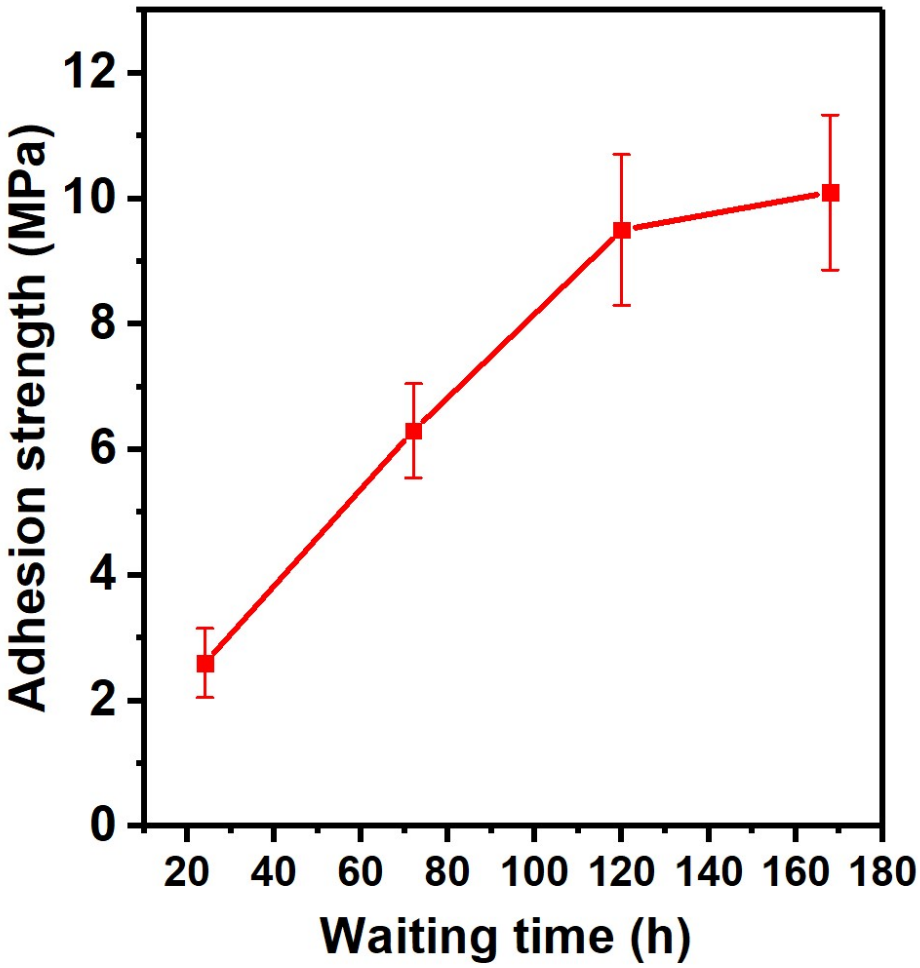

Supplementary Fig. 34 | Adhesion strength of P(4HMA$_{8\%}$-*co*-S$_{92\%}$) on aluminum surfaces with different waiting times. The results represent the mean ± s.d. (*n* = 5).

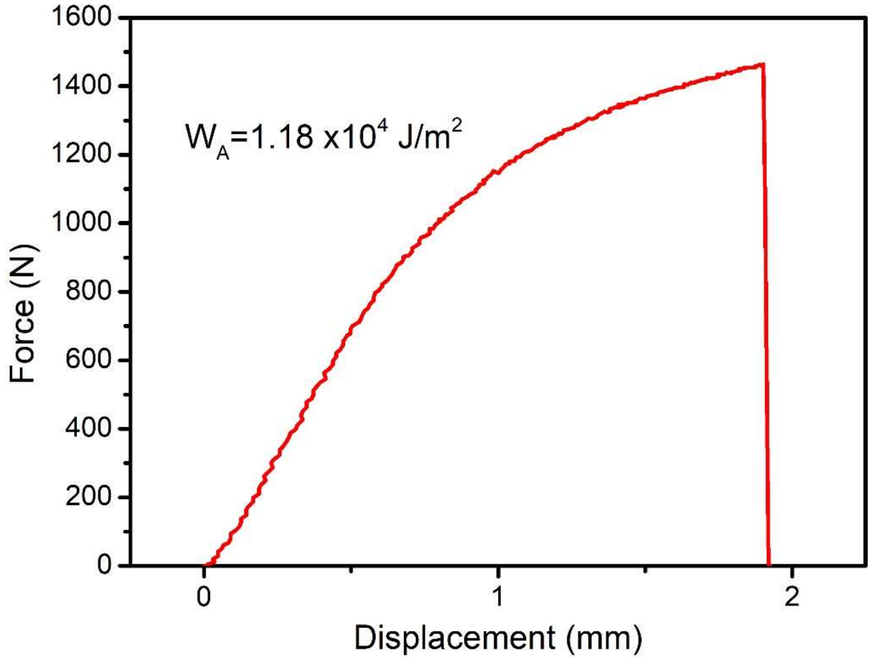

Supplementary Fig. 35 | Typical force-displacement curve during the adhesion tests. This result was presented by P(4HMA$_{8\%}$-*co*-S$_{92\%}$) on steel surfaces.

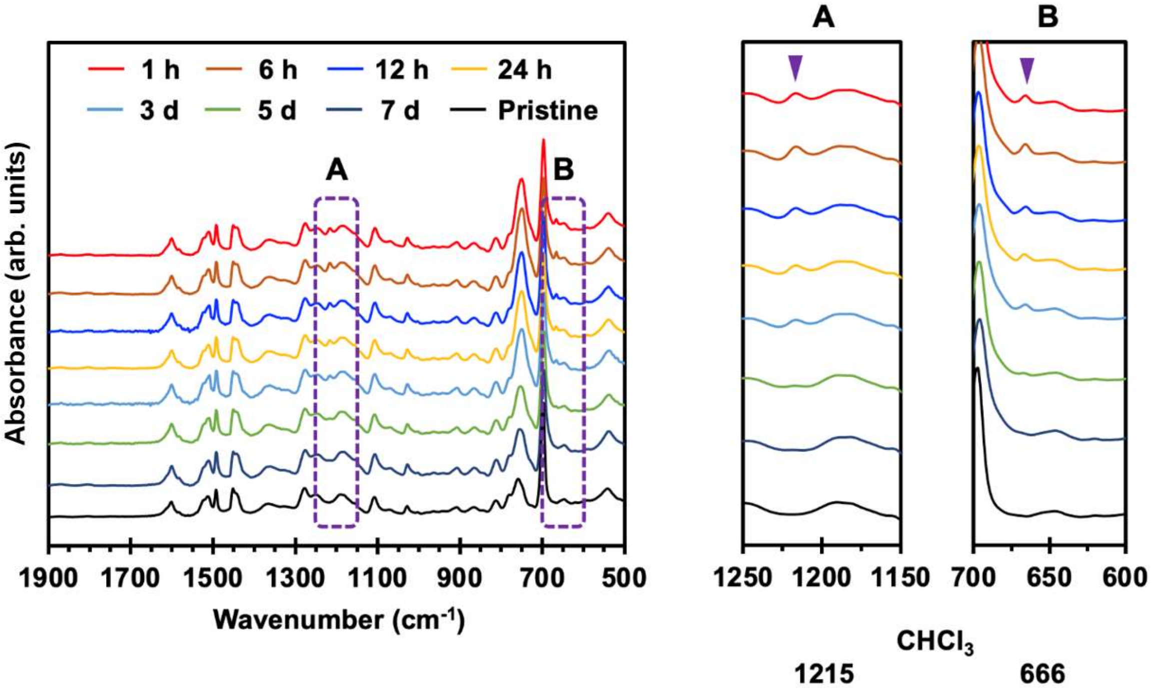

Supplementary Fig. 36 | The $CHCl_3$ remained in the P(2HS-*co*-S) adhesive layer traced by FT-IR.

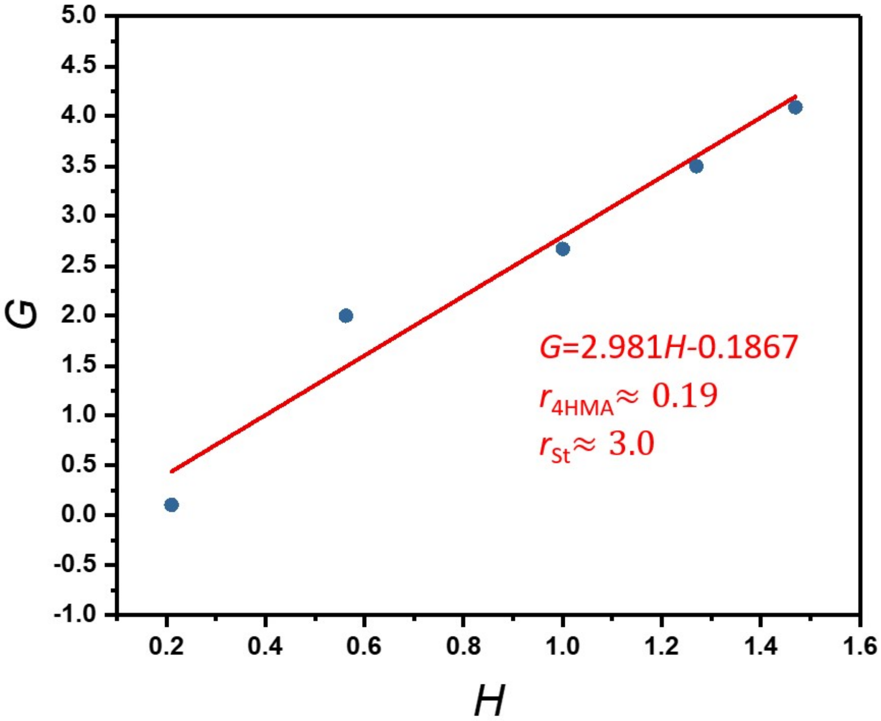

Supplementary Fig. 37 | Reactivity ratios of 4HMA and styrene as calculated by the Fineman-Ross Method.

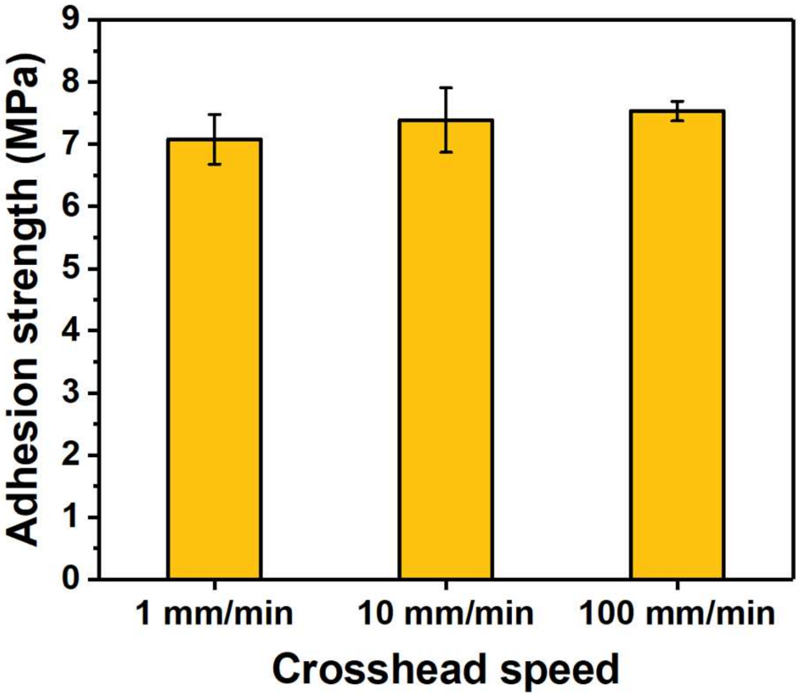

Supplementary Fig. 38 | Adhesion strength of P(4HS$_{9\%}$-*co*-S$_{91\%}$) on aluminum surfaces as measured with different crosshead speeds. The results represent the mean ± s.d. (*n* = 5).

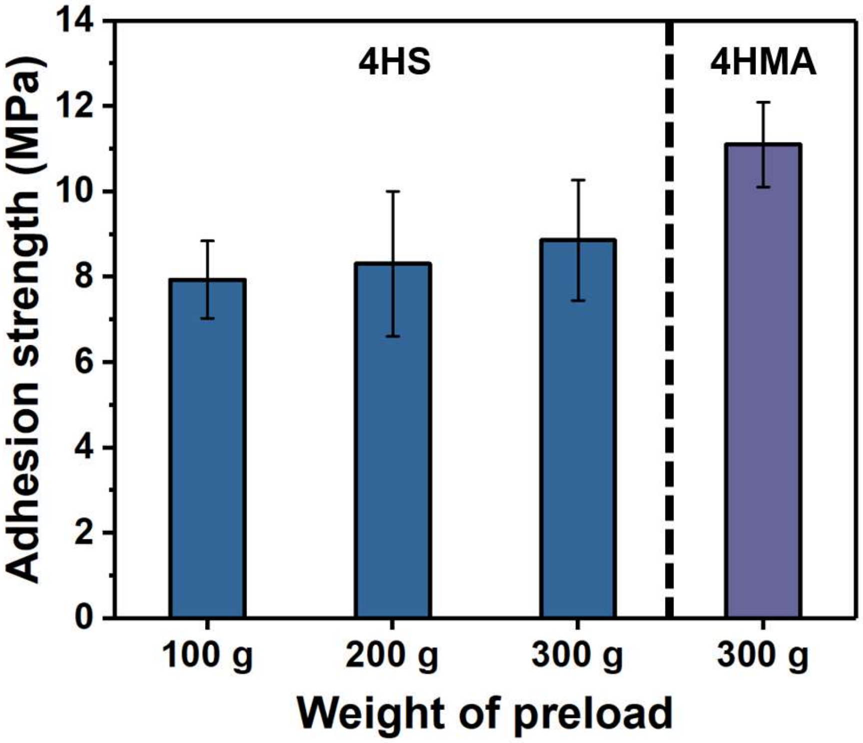

Supplementary Fig. 39 | Adhesion strength of P(4HS$_{9\%}$-*co*-S$_{91\%}$) and P(4HMA$_{8\%}$-*co*-S$_{92\%}$) on aluminum surfaces with different preload weights. The results represent the mean ± s.d. (*n* = 5).

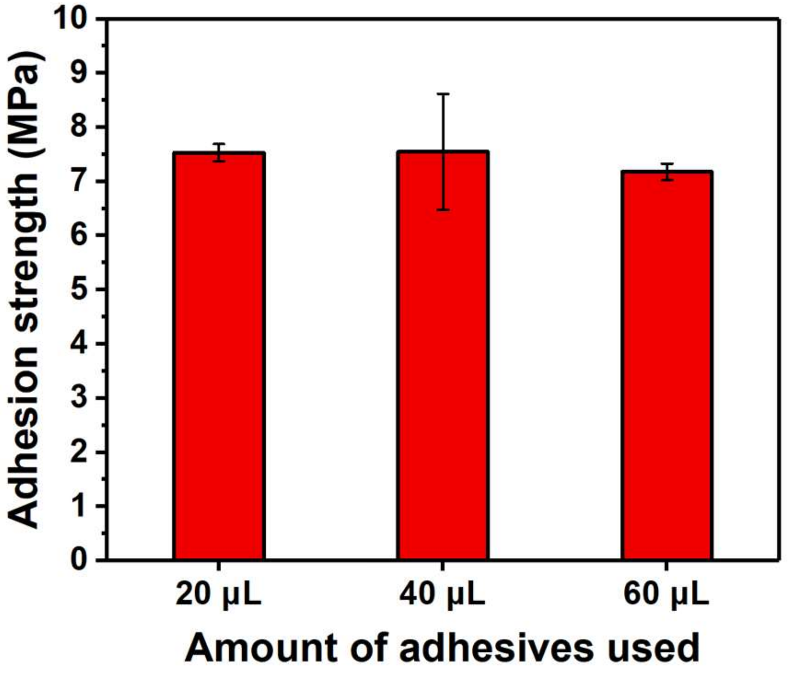

Supplementary Fig. 40 | Adhesion strength of P(4HS$_{9\%}$-*co*-S$_{91\%}$) on aluminum surfaces using different amounts of adhesives used. The results represent the mean ± s.d. (*n* = 5).

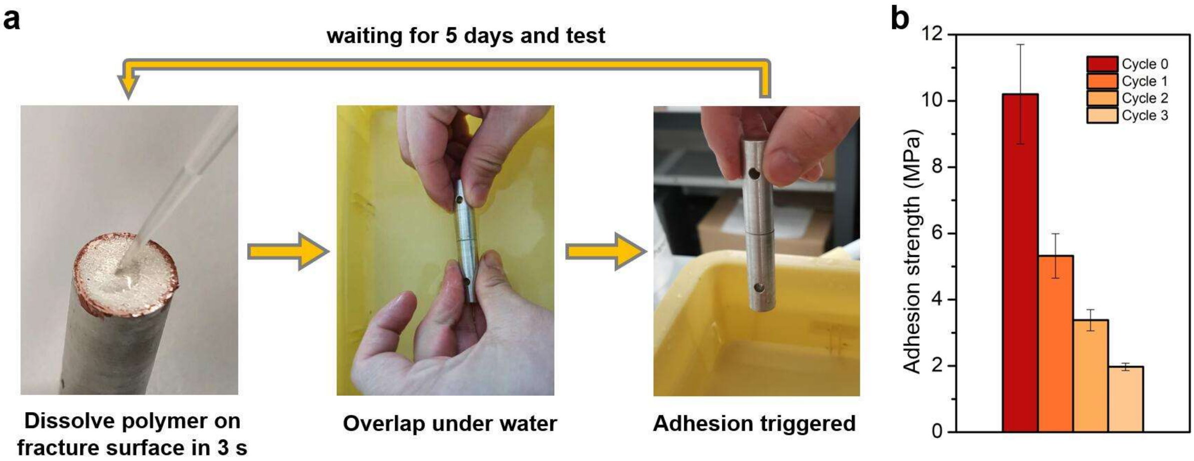

Supplementary Fig. 41 | a, Process for recycling P(4HMA$_{8\%}$-*co*-S$_{92\%}$) as an adhesive on a fractured aluminum surface. b, Adhesion strength per cycle using the process in section a. The results represent the mean ± s.d. (n = 5).

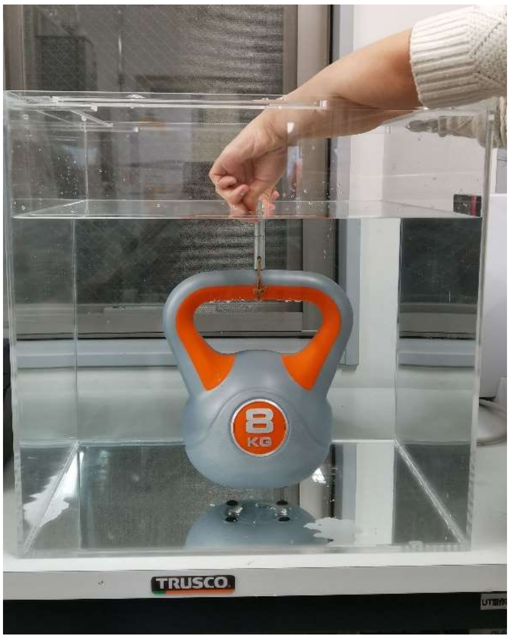

Supplementary Fig. 42 | A bonded aluminum rod raises 8-kg.

5. Supplementary References

1. Zhan, K., Ejima, H. & Yoshie, N. Antioxidant and Adsorption Properties of Bioinspired Phenolic Polymers: A Comparative Study of Catechol and Gallol. *ACS Sustain. Chem. Eng.* **4**, 3857–3863 (2016).
2. Zhan, K., Kim, C., Sung, K., Ejima, H. & Yoshie, N. Tunicate-Inspired Gallol Polymers for Underwater Adhesive: A Comparative Study of Catechol and Gallol. *Biomacromolecules* **18**, 2959–2966 (2017).
3. Plimpton, S. Fast Parallel Algorithms for Short-Range Molecular Dynamics. *J. Comput. Phys.* **117**, 1–19 (1995).
4. Shin, Y. K., Kwak, H., Vasenkov, A. V, Sengupta, D. & van Duin, A. C. T. Development of a ReaxFF Reactive Force Field for Fe/Cr/O/S and Application to Oxidation of Butane over a Pyrite-Covered Cr2O3 Catalyst. *ACS Catal.* **5**, 7226–7236 (2015).
5. Nosé, S. A unified formulation of the constant temperature molecular dynamics methods. *J. Chem. Phys.* **81**, 511–519 (1984).
6. Hoover, W. G. Canonical dynamics: Equilibrium phase-space distributions. *Phys. Rev. A* **31**, 1695–1697 (1985).
